# Synthesis of α‑Substituted β,γ-Unsaturated Cyclobutanecarboxamides via Palladium-Catalyzed Aminocarbonylation of Vinylcyclobutanols

**DOI:** 10.1021/acs.orglett.5c03781

**Published:** 2025-10-08

**Authors:** Yu-Kun Liu, Xiao-Feng Wu

**Affiliations:** † 28392 Leibniz-Institut für Katalyse e. V., Albert-Einstein-Straße 29a, 18059 Rostock, Germany; ‡ Dalian National Laboratory for Clean Energy, Dalian Institute of Chemical Physics, Chinese Academy of Sciences, Dalian, 116023, Liaoning, China

## Abstract

Vinylcyclobutanols are highly strained molecules that are prone to semipinacol rearrangement or ring-opening under catalytic conditions, challenging the synthesis of functionalized cyclobutane scaffolds. We report here a palladium-catalyzed aminocarbonylation of vinylcyclobutanols with amine hydrochlorides and CO. The reaction proceeds via in situ formation of conjugated dienes and π-allyl palladium intermediates, furnishing α-substituted β,γ-unsaturated cyclobutanecarboxamides. This method shows a broad substrate scope and excellent functional group tolerance while effectively suppressing rearrangement and ring-opening. It provides a concise, robust route to structurally diverse α-substituted β,γ-unsaturated cyclobutane derivatives bearing a quaternary carbon center.

## Graphical abstract

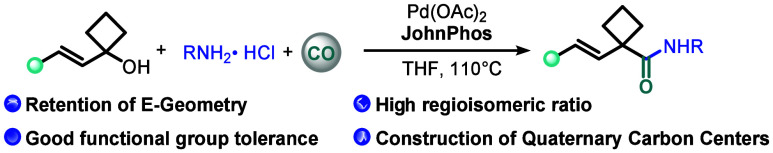

Substances incorporating cyclobutane structural motifs are frequently encountered in a variety of bioactive molecules and complex natural products (Scheme 1a),^1^ including alkaloids, steroids, and terpenoids.^2^ The inherent ring strain and conformational rigidity of a four-membered cyclobutane ring contribute to its unique chemical reactivity and structural diversity, which in turn play a crucial role in modulating the biological activity of these compounds.^3^ However, the high ring strain and the frequent presence of multiple stereocenters in the cyclobutane scaffold pose significant synthetic challenges,^4^ making the efficient and regioselective construction of such frameworks a long-standing focus in synthetic organic chemistry.^5^

**Scheme 1:**
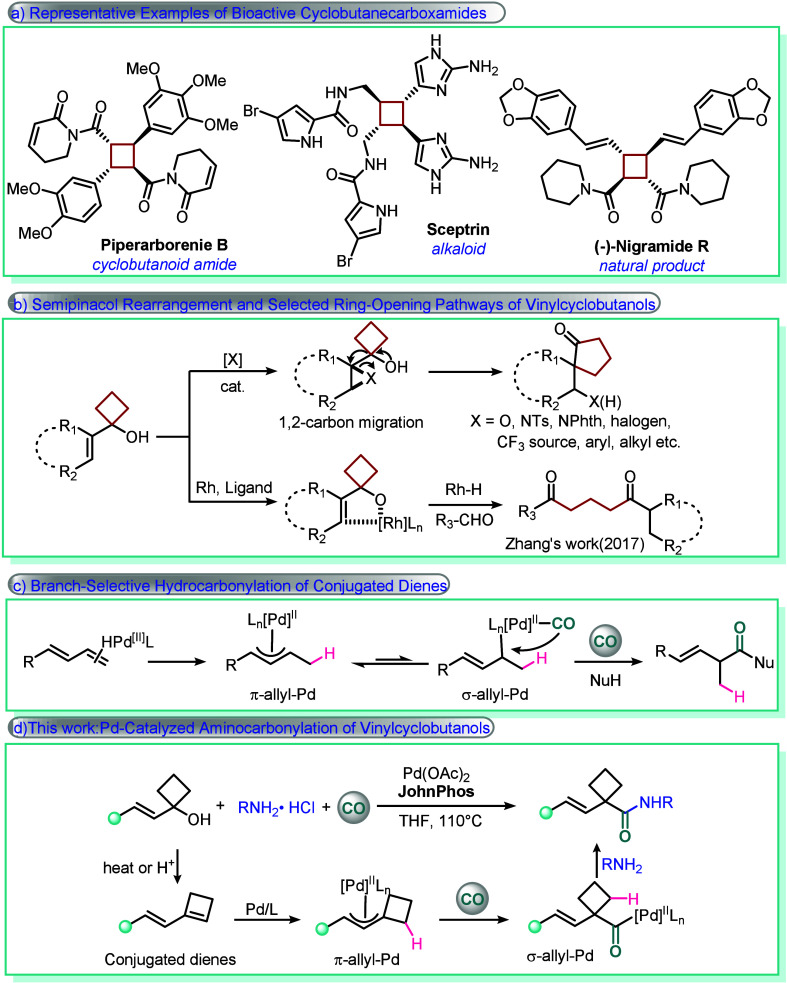
Reaction Overview of Vinyl Cyclobutanol and Palladium-Catalyzed Hydrocarbonylation of Conjugated Dienes

Vinylcyclobutanols, containing both an unsaturated bond and a strained four-membered all-carbon ring, are widely utilized in organic synthesis.^6^ The intrinsic instability of the cyclobutane ring, arising from pronounced ring strain, renders it highly susceptible to cleavage under a variety of reaction conditions.^7^ As a consequence, maintaining the integrity of the four-membered ring during synthetic operations remains a formidable challenge.^8^ In particular, these compounds tend to undergo semipinacol rearrangements, which proceed through 1,2-migration,^9^ or open to form ketones through metal-mediated oxidation^10^ (Scheme 1b) or radical-initiated pathways,^11^ making the preservation of the intact ring system highly challenging.

In recent years, considerable efforts have been devoted to the transition-metal-catalyzed carbonylation of conjugated dienes.^12^ In typical Pd-catalyzed cases, such reactions proceed via the initial formation of a π-allyl-Pd intermediate, which subsequently isomerizes to a σ-allyl-Pd species, followed by CO insertion and nucleophilic attack (Scheme 1c).^13^ However, CO insertion can generate a π-allyl-Pd­(CO) complex that is often sufficiently stable to hinder its isomerization to the corresponding σ-allyl-Pd­(CO) complex in the presence of CO.^14^ However, beyond these mechanistic constraints, reported carbonylation reactions of conjugated dienes have predominantly focused on less sterically hindered dienes, such as 1,3-butadiene,^12b,12h,12j,12k^ isoprene,^12c,12e^ and 4-phenyl-1,3-butadiene.^12g,12i^ In contrast, studies involving conjugated dienes bearing strained ring systems remain at a preliminary stage, underscoring the need for new strategies to enable the efficient carbonylation of such substrates.

Herein, we report a novel palladium-catalyzed aminocarbonylation reaction employing substituted vinylcyclobutanols, carbon monoxide, and amine hydrochlorides as starting materials to access α-substituted β,γ-unsaturated cyclobutane­carboxamides. This method exhibits high branch selectivity, broad substrate scope, and excellent functional group tolerance, while also enabling the construction of a cyclobutane core bearing quaternary carbon centers.

Initially, the model reaction was conducted using vinylcyclobutanol **1a** and aniline hydrochloride **2a** as the starting substrates (Table 1). Under the standard conditions of Pd­(OAc)_2_ as the catalyst, P­(*o*-tol)_3_ as the ligand, and THF as the solvent at 110 °C, desired product **3a** was obtained in 61% yield (Table 1, entry 1). However, replacing THF with DMF, PhCF_3_, or toluene resulted in varying degrees of yields decreasing (Table 1, entry 2). Furthermore, when palladium catalysts with different oxidation states were employed, the reaction still afforded the target product in moderate yields (Table 1, entries 3, 4). In terms of ligand effects, screening revealed that the use of the less sterically hindered ligand **L2** led to a pronounced decrease in yield (Table 1, entry 5), whereas increasing the steric bulk based on the **L2** scaffold improved the yield (Table 1, entry 6), indicating that ligands with greater steric hindrance are more effective in this reaction. Additionally, the presence of an *o*-methoxy substituent slightly enhanced the yield (Table 1, entry 7), whereas **L5** exerted a minimal influence on the reaction outcome (Table 1, entry 8). In contrast, other ligands such as PCy_3_, P­[(3,5-(CF_3_)_2_C_6_H_3_]_3_, and RuPhos delivered unsatisfactory results (Table 1, entry 9). Notably, employing ligand **L9** significantly boosted the reaction efficiency, thereby affording the corresponding product in 94% isolated yield (Table 1, entry 10). In addition, replacing aniline hydrochloride with aniline and HCl (4 M in dioxane) led to a significant yield reduction (Table 1, entry 11). Moreover, lowering the pressure, decreasing the temperature, or extending the reaction time proved detrimental to the reaction (Table 1, entries 12–14). It is worth mentioning that various regularly used bidentate phosphine ligands were also tested but led to no desired product detected.

**Table 1:**
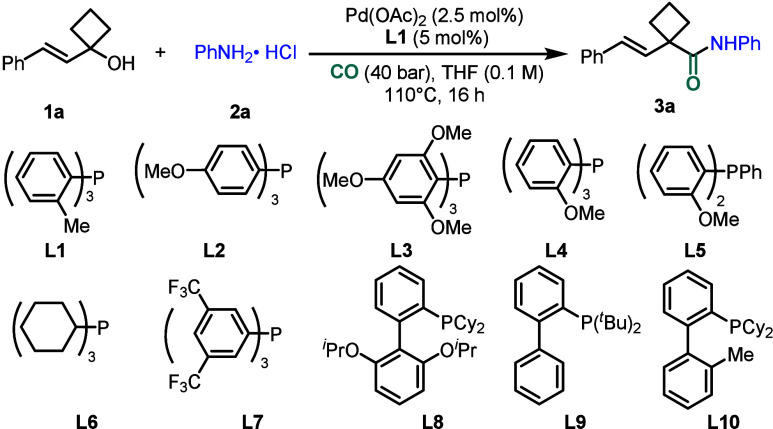
Optimization of the Reaction Conditions^a^ ^,^ ^b^

Entry	Variation from standard conditions	**3a** (%)
1	None	61
2	DMF, PhCF_3_, Toluene as solvent	45–50
3	Pd(TFA)_2_ instead of Pd(OAc)_2_	43
4	Pd(PPh_3_)_4_ instead of Pd(OAc)_2_	49
5	**L2** instead of **L1**	28
6	**L3** instead of **L1**	52
7	**L4** instead of **L1**	67
8	**L5** instead of **L1**	63
9	**L6**-**L8**, **L10** instead of **L1**	26–43
10	**L9** instead of **L1**	96(94)^c^
11^d^ ^,^ ^e^	PhNH_2_ with 1 equiv HCl	65
12^d^	30 bar CO	60
13^d^	100 °C	77
14^d^	24 h	86

a Unless otherwise noted, the reactions were performed with Pd­(OAc)_2_ (2.5 mol %), **Ligand** (5 mol %), **1a** (0.12 mmol), **2a** (0.1 mmol), CO (40 bar), and THF (1.0 mL) at 110 °C for 16 h.

b The yields were determined by GC with *n*-hexadecane as internal standard.

c Isolated yield of **3a**.

d **L9** instead of **L1**.

e 4 M HCl in dioxane was used.

With optimal conditions established, the general applicability of this method was demonstrated (Scheme 2). Single-crystal X-ray diffraction analysis unambiguously established the geometric *E* configuration of **3a**. Initially, the effect of methyl group position on the benzene ring was investigated. When the methyl group was located at different positions, the yields of the corresponding products decreased slightly (**3b**–**3d**). In contrast, the introduction of a methoxy group exerted little influence on the reaction outcome; for instance, when the methoxy group was located at the *meta*-position, the corresponding product was obtained in 84% yield (**3e**). Notably, the 4-fluoroaniline derivative afforded the product in a significantly diminished yield (**3f**), whereas other halogenated anilines (Cl, Br) exhibited good compatibility under the standard reaction conditions (**3g**,**h**). In addition, substrates bearing cyano, benzyloxy, ester, or methanesulfonyl groups, as well as naphthyl rings, were all smoothly converted into the desired products (**3i–3m**). Furthermore, fluorinated substrates such as those containing a trifluoromethyl group also reacted efficiently, affording the products in high yields (**3n**–**3q**). The reaction of the fused aromatic group (**2r**) proceeded smoothly to give the corresponding cyclobutanecarboxamide **3r**. Moreover, multisubstituted anilines proved to be well-suited for this transformation, delivering the corresponding products in good yields (**3s**,**t**). Alkylamine was also transformed successfully and afforded the desired product **3u** in 48% yield. The decreased reaction efficiency might be due to its higher nucleophilicity. Furthermore, the method displayed good compatibility with amino acid derivatives (**3v**–**3y**), providing the corresponding products in moderate yields (34–57%). We further demonstrated the utility of the protocol in the late-stage functionalization of bioactive molecules, and the results showed that selected bioactive molecules could undergo the reaction to obtain the desired products (**3z**–**3ac**). Finally, the scope of vinyl cyclobutanols was examined. Substrates bearing 4-chloro, *tert*-butyl, and cyano on the benzene ring, as well as those containing methyl groups at different positions and 3,5-dimethyl, all afforded the corresponding products in good yields (**3ad**–**3ai**). In addition, the reaction exhibited good tolerance toward the benzene-fused heteroaromatic substrate, delivering the desired products efficiently (**3aj**). However, a messy reaction mixture was obtained when alkyl-substituted vinylcyclobutanol was tested under our standard conditions.

**Scheme 2:**
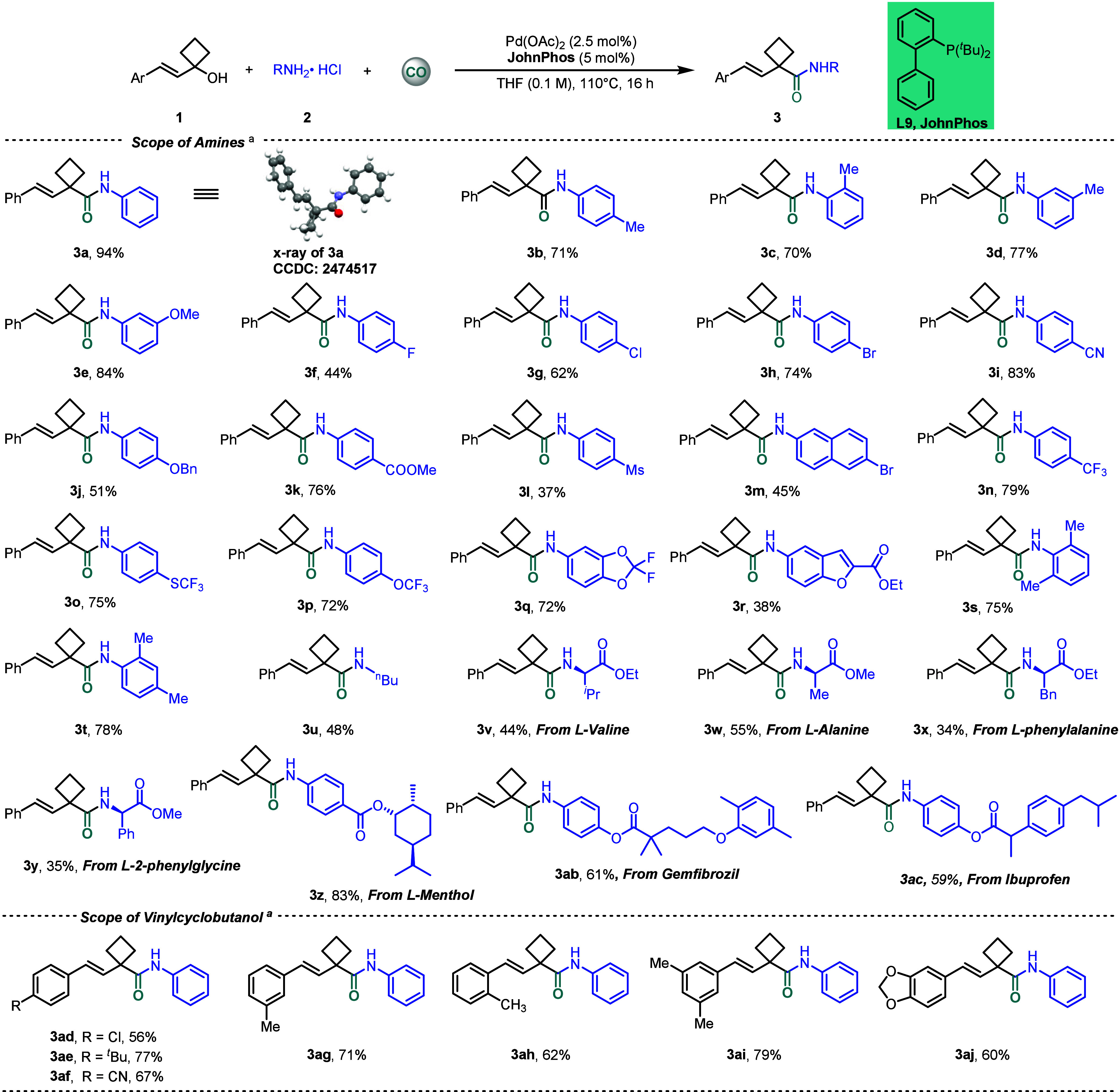
Scope of Amines and Vinylcyclobutanols

a The reactions were performed with Pd­(OAc)_2_ (2.5 mol %), JohnPhos (5 mol %), **1** (0.12 mmol), **2** (0.1 mmol), CO (40 bar), and THF (1.0 mL) at 110 °C for 16 h.

To further evaluate the synthetic utility of this methodology, transformation was carried out, as illustrated in Scheme 3. In consideration of the practicability, a gram-scale reaction was performed, affording 0.227 g of the desired product **3a** in 77% yield (Scheme 3a). Epoxidation of the α-substituted β,γ-unsaturated cyclobutanecarboxamide **3a** can be realized under mild conditions and afforded the target epoxidized cyclobutanecarboxamides **4** in 63% yield (Scheme 3b).

**Scheme 3:**
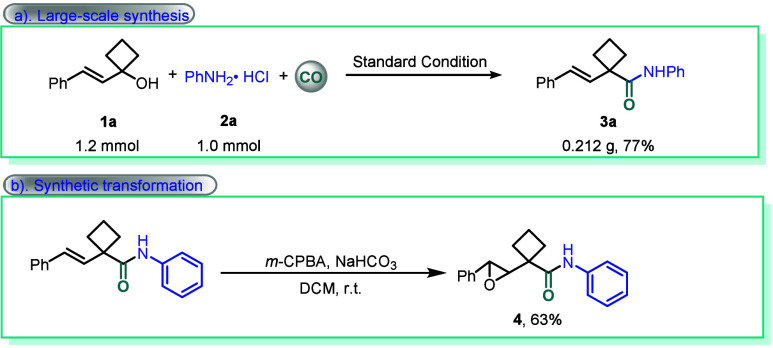
Synthetic Applicability

To further understand the mechanism, a verification experiment has confirmed that the conjugated diene is a key intermediate in this reaction (Scheme 4a). Based on the experimental results presented above and in the literature,^15^ a plausible reaction mechanism is proposed (Scheme 4b). Initially, under heating or in the presence of acid, vinylcyclobutanol was transformed into conjugated diene **5′**. Concurrently, the palladium catalyst reacted with the ligand and either H_2_O or acid to generate a Pd–H complex **A**. Subsequently, coordination of the conjugated diene **5′** to the palladium hydride species **A**, followed by migratory insertion, generates an π-allyl-palladium intermediate, existing as two regioisomeric forms **B** and **B′**. Between them, isomer **B′** is better suited for subsequent carbon monoxide coordination and insertion, furnishing the corresponding acyl-palladium complex **C**. Finally, aminolysis of intermediate **C** by the amine nucleophile delivers the desired amide product and concurrently regenerates palladium hydride catalyst **A**, thus completing the catalytic cycle.

**Scheme 4:**
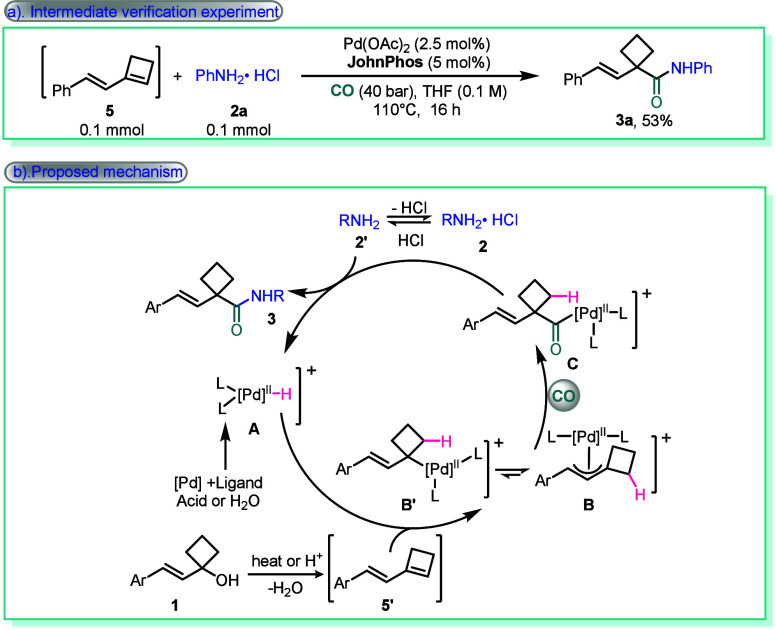
Mechanistic Studies

In summary, we have developed an unprecedented and efficient palladium-catalyzed aminocarbonylation reaction using vinylcyclobutanols as starting materials in combination with carbon monoxide and amine hydrochlorides. This transformation exhibits a broad substrate scope, excellent functional group tolerance, and good compatibility with bioactive molecule derivatives. Various α-substituted β,γ-unsaturated cyclobutanecarboxamides bearing quaternary carbon centers were synthesized in good to excellent yields under the standard conditions. Notably, the methodology effectively suppresses undesired semipinacol rearrangement and ring-opening pathways typically associated with the high strain of vinylcyclobutanols. Mechanistic studies suggest that in situ generated conjugated dienes serve as crucial intermediates, with the subsequent formation of π-allylpalladium and its isomerization to σ-allylpalladium complexes representing key steps in the catalytic cycle.

## Supplementary Material



## Data Availability

The data underlying this study are available in the published article and its Supporting Information

## Notes

Experimental details, characterization data, X-ray crystallographic data of product **3a**, and NMR spectra (PDF)
