# Supplementary material for: Synthesis of α‑Substituted β,γ-Unsaturated Cyclobutanecarboxamides via Palladium-Catalyzed Aminocarbonylation of Vinylcyclobutanols
Source: Org Lett. 2025 Oct 8;27(41):11644–9. doi: 10.1021/acs.orglett.5c03781 (PMC12538587; doi:10.1021/acs.orglett.5c03781)

Supporting Information

Synthesis of α-Substituted β,γ-Unsaturated Cyclobutanecarboxamides via Palladium-Catalyzed Aminocarbonylation of Vinylcyclobutanols

Yu-Kun Liu,[a] Xiao-Feng Wu[a,b]*

[a]Leibniz-Institut für Katalyse e. V., Albert-Einstein-Straße 29a, 18059 Rostock, Germany.
[b]Dalian National Laboratory for Clean Energy, Dalian Institute of Chemical Physics, Chinese Academy of Sciences, Dalian, 116023, Liaoning, China. E-mail: xwu2020@ dicp.ac.cn

Contents

1.General information 1
2. Optimization of Reaction Conditions 2
3. General procedure for the preparation of vinylcyclobutanols 4
3.1 List of vinylcycobutanols 4
3.2 Typical procedure 4
4.General procedure for the synthesis of Cyclobutanecarboxylates 4
5. Spectroscopic Data of Products 5
6.Synthetic transformations of 3a 14
6.1 Epoxidation of compound 3a 14
7. Synthesis of **5** and transformation 15
8. X-ray Crystallographic Data of Product 3a 17
9.References 18
10. NMR Spectra 19

1.General information

Reagents and solvents: Unless otherwise noted, reagents were ordered from *Sigma-Aldrich, TCI, ABCR, Alfa Aesar* or *BLD pharm*, and used without purification. Pure solvents was available from *Thermo Fisher*, and degassed (3 times) under argon atmosphere, then store under standard Schlenk technique (anhydrous and under inert atmosphere).

Purification: Analytical thin layer chromatography was performed using *MACHEREY-NAGEL Gmbn & Co. KG* silica gel plates (Silica gel 60 UV_{254}). Visualization was by ultraviolet fluorescence (λ = 254 nm) and/or staining with potassium permanganate ($KMnO_4$). The products were isolated from the reaction mixture by column chromatography on silica gel 60, 0.063-0.2 mm, 70-230 mesh (Merck). Gradient flash chromatography was conducted eluting with PE/EA, PE refers to pentane and EA refers to ethyl acetate, they were listed as volume/volume ratios. Important to note that due to the presence of polymer as radical inhibitor in the solvent used for flash chromatography, some signals can be found in aliphatic area in ^{1}H NMR. Hence the purity of the products is considered as 95% and considered in the yield calculation.

Data collection: GC analysis was performed on an Agilent HP-7890A instrument with FID detector and HP-5 capillary column (polydimethylsiloxane with 5% phenyl groups, 30 m, 0.32 mm i.d., 0.25 μm film thickness) using argon as carrier gas. Electron impact (EI) mass spectra were recorded on AMD 402 mass spectrometer (70 eV). The data are given as mass units per charge (m/z). High resolution mass spectra (HRMS) were recorded on Agilent 6210. NMR spectra were recorded on Bruker Avance 300 and Bruker ARX 400 spectrometers. Multiplets were assigned as s(singlet), d (doublet), t (triplet), q (quartet), dd (doublet of doublet), m (multiplet) and br (broad). s (singlet). Chemical shifts (ppm) are given relative to solvent: references for $CDCl_3$ were 7.26 ppm (^{1}H NMR) and 77.00 ppm (^{13}C NMR). All measurements were carried out at room temperature unless otherwise stated.

NOTE: Because of the high toxicity of carbon monoxide, all the reactions should be performed in an autoclave. The laboratory should be well-equipped with a CO detector and alarm system.

2. Optimization of Reaction Conditions

Table S1. Optimization of solvent

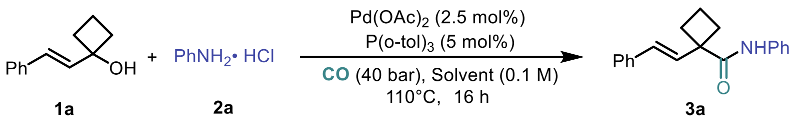

Entry	Sovlent	Yield [b]
1	THF	61%
2	Toluene	50%
3	NMP	45%
4	MeCN	Trace
5	$PhCF_3$	45%
6	DCM	20%
7	DMF	49%
8	DMSO	N.D

[a] $Pd(OAc)_2$ (2.5 mol%), ligand (5 mol%) **1a** (0.12 mmol), **2a** (0.1 mmol), CO (40 bar), Solvent (1 mL) at 110 ℃ for 16 h. [b] The yield was determined by GC with n-hexadecane as internal standard.

Table S2. Optimization of catalysis

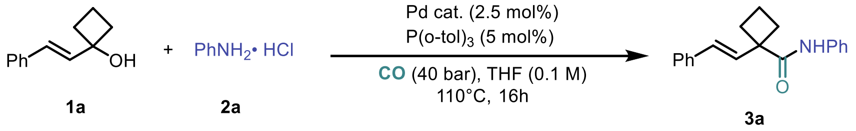

Entry	Pd cat.	Yield [b]
1	$Pd(OAc)_2$	61%
2	$Pd(TFA)_2$	43%
3	$Pd(acac)_2$	39%
4	$[Pd(\pi\text{-cinnamyl})Cl]_2$	42%
5	$Pd(CH_3CN)_2Cl_2$	37%
6	$PdCl_2$	46%
7	$Pd(PPh_3)_4$	49%

[a] Pd cat. (2.5 mol%), ligand (5 mol%), **1a** (0.12 mmol), **2a** (0.1 mmol), CO (40 bar), THF (1 mL) at 110 ℃ for 16 h. [b] The yield was determined by GC with n-hexadecane as internal standard.

Table S3. Optimization of ligand

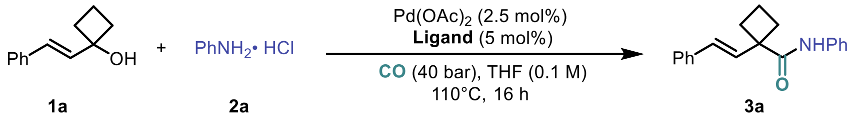

Entry	Ligand	Yield [b]
1	**L1**	61%
2	**L2**	28%
3	**L3**	52%
4	**L4**	67%
5	**L5**	63%
6	**L6**	27%
7	**L7**	26%
8	**L8**	43%
9	**L9**	96% (94%)
10	**L10**	31%

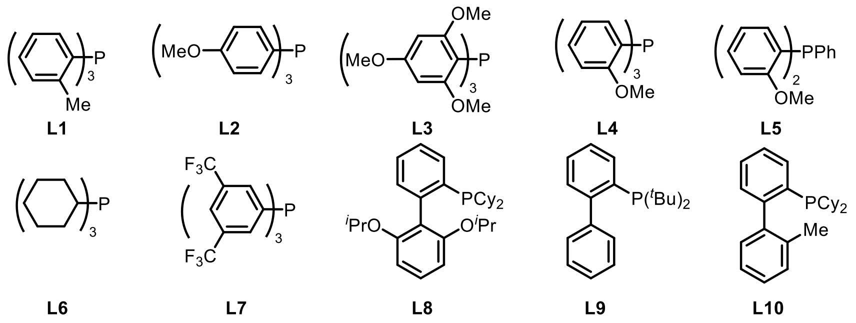

[a] $Pd(OAc)_2$ (2.5 mol%), ligand (5 mol%) **1a** (0.12 mmol), **2a** (0.1 mmol), CO (40 bar), THF (1 mL) at 110 °C for 16 h. [b] The yield was determined by GC with n-hexadecane as internal standard. Isolated yield in the parentheses.

Table S4. Optimization of the amount of acid

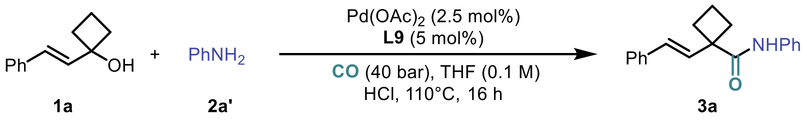

Entry	Amount of HCl	Yield [b]
1	0.5 equiv.	30%
2	1.0 equiv.	65%
3	1.5 equiv.	65%
4	2.0 equiv.	67%

[a] $Pd(OAc)_2$ (2.5 mol%), ligand (5 mol%) **1a** (0.12 mmol), **2a'** (0.1 mmol), CO (40 bar), HCl (4 M in 1,4-dioxane), THF (1 mL) at 110 °C for 16 h. [b] The yield was determined by GC with n-hexadecane as internal standard.

Table S5. Optimization of temperature, pressure, time and amount of 1a.

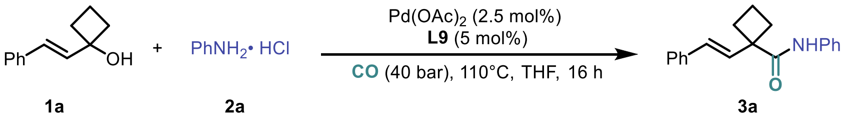

Entry[a]	Amount of **1a**	Temperature(°C)	Pressure (bar)	Time (h)	Yield[b]
1	0.1 mmol (1 equiv.)	110	40	16	58%
2	0.12 mmol (1.2 equiv.)	110	40	16	96% (94%)
2	0.15 mmol (1.5 equiv.)	110	40	16	94%
3	0.12 mmol (1.2 equiv.)	110	30	16	60%
4	0.12 mmol (1.2 equiv.)	110	20	16	33%
5	0.12 mmol (1.2 equiv.)	100	40	16	77%
6	0.12 mmol (1.2 equiv.)	110	40	24	86%
7[c]	0.12 mmol (1.2 equiv.)	110	40	16	52%
8[d]	0.12 mmol (1.2 equiv.)	110	40	16	72%

[a] $Pd(OAc)_2$ (2.5 mol%), **L9** (5 mol%), **1a**, **2a** (0.1 mmol), THF. [b] The yield was determined by GC with n-hexadecane as internal standard. Isolation yields in parentheses. [c] 0.5 mL THF. [d] 1.5 mL THF.

3. General procedure for the preparation of vinylcyclobutanols

3.1 List of vinylcycobutanols

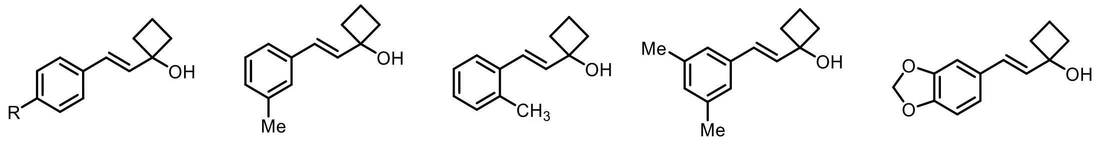

1a, R = H
1b, R = Cl
1c, R = ^{t}Bu
1d, R = CN

1e **1f** **1g** **1h**

All alkenyl cyclobutanols **1a-1h** were prepared according to the reported procedure and their characterization data match the reported data.[1]

3.2 Typical procedure

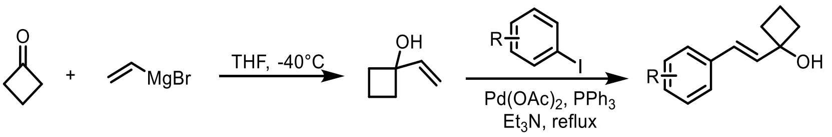

A one-pot procedure for synthesis of alkenyl cyclobutanols was realized. To a solution of cyclobutanone (0.7 g, 10 mmol) in anhydrous THF (10 mL) at -40 °C was added vinyl magnesium bromide (15 mmol., 21.4 mL, 0.7 M solution in THF) over 20 min. The mixture was stirred at room temperature for 6 h. The reaction was quenched by the addition of a saturated solution of NH_4Cl. The organic layer was extracted with diethyl ether. The combined organics were washed with brine, dried with Na_2SO_4 and concentrated in vacuo. Next, the mixture of 1-vinylcyclobutanol, substituted iodobenzene (10 mmol), $Pd(OAc)_2$ (0.1 mmol), and triphenylphosphine (0.2 mmol) in triethylamine (10 mL) was flushed with Ar and heated to reflux for 12 h. The reaction mixture was then concentrated in vacuo to remove trimethylamine and diluted with 20 mL of ethyl acetate. The resulting suspension was filtered and washed with H_2O, brine, dried (Na_2SO_4) and concentrated in vacuo. Pure configurations of alkenyl cyclobutanols were purified by silica gel column chromatography (pentane/ethyl acetate = 20/1 to 5/1, v/v).

4.General procedure for the synthesis of Cyclobutanecarboxylates

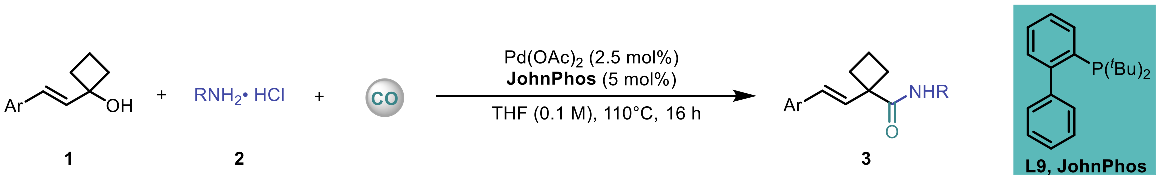

A vial (4 mL) was charged with $Pd(OAc)_2$ (2.5 mol%, 0.6 mg), JohnPhos (5 mol%, 1.5 mg), cyclobutanol (1.2 equiv., 0.12 mmol,), amine hydrochlorides (1.0 equiv., 0.1 mmol) and a stirring bar. The vial was closed by PTFE/white rubber septum (Wheaton 13 mm Septa) and phenolic cap and connected with atmosphere with a needle. The vial was evacuated under vacuum and recharged with argon for three times. After that, THF (1.0 mL) were injected under argon by using a syringe. Subsequently, the vial (or several vials) was placed in an alloy plate, which was transferred into a 300 mL autoclave of the 4560 series from Parr Instruments. After flushing the autoclave three times with CO, a pressure of 40 bar of CO was adjusted at ambient temperature. Then, the reaction was performed for 16 h at 110 °C (aluminum block). After 16 hours, the autoclave was cooled down with ice water to room temperature and the pressure was released carefully. The solution was concentrated in vacuo then purified by silica-gel column chromatography using pentane and ethyl acetate to afford the corresponding product **3**.

5. Spectroscopic Data of Products

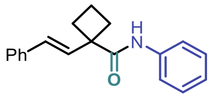

(E)-N-phenyl-1-styrylcyclobutane-1-carboxamide (3a)

25.9 mg, 94% yield, white solid. Eluent (Pentane/Ethyl Acetate) = 15:1, R_f = 0.2.

^{1}H NMR (300 MHz, CDCl$_3$) δ 7.54 – 7.48 (m, 2H), 7.48 – 7.43 (m, 2H), 7.41 – 7.27 (m, 5H), 7.22 (br, 1H), 7.09 (ddt, *J* = 7.8, 7.0, 1.2 Hz, 1H), 6.71 (d, *J* = 16.1 Hz, 1H), 6.50 (d, *J* = 16.1 Hz, 1H), 2.81 – 2.69 (m, 2H), 2.35 – 2.22 (m, 2H), 2.08 – 1.95 (m, 2H).

^{13}C NMR (75 MHz, CDCl$_3$) δ 173.3, 138.1, 136.5, 131.5, 130.7, 129.1, 128.9, 128.1, 126.6, 124.3, 119.8, 51.7, 30.5, 16.1.

HRMS (ESI) (m/z): [M + Na]$^+$ Calcd for $C_{19}H_{19}NONa$ 300.1359; Found: 300.1365.

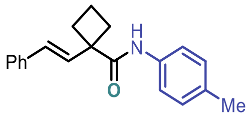

(E)-1-styryl-N-(p-tolyl)cyclobutane-1-carboxamide (3b)

21.0 mg, 71% yield, white solid. Eluent (Pentane/Ethyl Acetate) = 15:1, R_f = 0.2.

^{1}H NMR (300 MHz, CDCl$_3$) δ 7.48 – 7.42 (m, 2H), 7.41 – 7.33 (m, 4H), 7.31 – 7.27 (m, 1H), 7.15 (br, 1H), 7.12 – 7.08 (m, 2H), 6.70 (d, *J* = 16.1 Hz, 1H), 6.50 (d, *J* = 16.1 Hz, 1H), 2.80 – 2.67 (m, 2H), 2.33 – 2.22 (m, 5H), 2.08 – 1.93 (m, 2H).

^{13}C NMR (75 MHz, CDCl$_3$) δ 173.2, 136.5, 135.5, 133.9, 131.6, 130.6, 129.6, 128.9, 128.1, 126.6, 119.8, 51.7, 30.5, 21.0, 16.1.

HRMS (ESI) (m/z): [M + Na]$^+$ Calcd for $C_{20}H_{21}NONa$ 314.1515; Found: 314.1519.

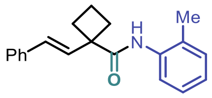

(E)-1-styryl-N-(o-tolyl)cyclobutane-1-carboxamide (3c)

20.3 mg, 70% yield, white solid. Eluent (Pentane/Ethyl Acetate) = 20:1, R_f = 0.3.

^{1}H NMR (300 MHz, CDCl$_3$) δ 7.88 (d, *J* = 8.1 Hz, 1H), 7.40 – 7.35 (m, 2H), 7.32 – 7.25 (m, 2H), 7.24 – 7.19 (m, 1H), 7.19 – 7.10 (m, 2H), 7.07 – 7.03 (m, 1H), 6.99 – 6.93 (m, 1H), 6.66 (d, *J* = 16.1 Hz, 1H), 6.46 (d, *J* = 16.1 Hz, 1H), 2.77 – 2.62 (m, 2H), 2.28 – 2.17 (m, 2H), 2.04 (s, 3H), 2.02 – 1.86 (m, 2H).

^{13}C NMR (75 MHz, CDCl$_3$) δ 173.1, 136.5, 136.0, 132.0, 130.9, 130.5, 128.9, 128.1, 127.0, 126.5, 124.8, 122.1, 51.8, 30.6, 17.7, 16.3.

HRMS (ESI) (m/z): [M + Na]$^+$ Calcd for $C_{20}H_{21}NONa$ 314.1515; Found: 314.1522.

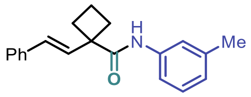

(E)-1-styryl-N-(m-tolyl)cyclobutane-1-carboxamide (3d)

22.4 mg, 77% yield, white solid. Eluent (Pentane/Ethyl Acetate) = 20:1, R_f = 0.3.

^{1}H NMR (300 MHz, CDCl$_3$) δ 7.49 – 7.43 (m, 2H), 7.40 – 7.33 (m, 3H), 7.32 – 7.27 (m, 2H), 7.21 – 7.15 (m, 2H), 6.91 (d, *J* = 7.5 Hz, 1H), 6.70 (d, *J* = 16.1 Hz, 1H), 6.50 (d, *J* = 16.1 Hz, 1H), 2.80 – 2.68 (m, 2H), 2.35 – 2.23 (m, 5H), 2.11 – 1.96 (m, 2H).

^{13}C NMR (75 MHz, CDCl$_3$) δ 173.3, 139.0, 138.0, 136.5, 131.6, 130.7, 128.9, 128.1, 126.6, 125.1, 120.4, 116.8, 51.8, 30.5, 21.6, 16.1.

HRMS (ESI) (m/z): [M + Na]$^+$ Calcd for $C_{20}H_{21}NONa$ 314.1515; Found: 314.1519.

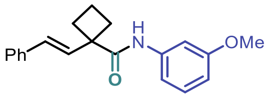

(E)-N-(3-methoxyphenyl)-1-styrylcyclobutane-1-carboxamide (3e)

25.9 mg, 84% yield, white solid. Eluent (Pentane/Ethyl Acetate) = 10:1, $R_f = 0.2$.

^{1}H NMR (400 MHz, CDCl$_3$) δ 7.42 – 7.35 (m, 2H), 7.31 – 7.26 (m, 3H), 7.23 – 7.19 (m, 1H), 7.15 – 7.08 (m, 2H), 6.84 (ddd, *J* = 8.0, 2.0, 0.9 Hz, 1H), 6.63 (d, *J* = 16.2 Hz, 1H), 6.57 (ddd, *J* = 8.3, 2.5, 0.9 Hz, 1H), 6.41 (d, *J* = 16.1 Hz, 1H), 3.72 (s, 3H), 2.70 – 2.62 (m, 2H), 2.25 – 2.17 (m, 2H), 1.99 – 1.88 (m, 2H).

^{13}C NMR (101 MHz, CDCl$_3$) δ 173.4, 160.3, 139.4, 136.4, 131.4, 130.8, 129.7, 128.9, 128.2, 126.6, 111.7, 110.5, 105.2, 55.5, 51.8, 30.5, 16.1.

HRMS (**ESI**) (m/z): $[M + H]^+$ Calcd for $C_{20}H_{22}NO_2$ 308.1645; Found: 308.1646.

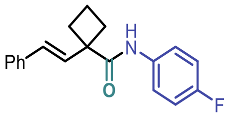

(E)-N-(4-fluorophenyl)-1-styrylcyclobutane-1-carboxamide (3f)

13.0 mg, 44% yield, white solid. Eluent (Pentane/Ethyl Acetate) = 15:1, $R_f = 0.2$.

^{1}H NMR (400 MHz, CDCl$_3$) δ 7.49 – 7.43 (m, 4H), 7.39 – 7.34 (m, 2H), 7.31 – 7.27 (m, 1H), 7.20 (br, 1H), 7.03 – 6.96 (m, 2H), 6.70 (d, *J* = 16.1 Hz, 1H), 6.49 (d, *J* = 16.1 Hz, 1H), 2.77 – 2.69 (m, 2H), 2.33 – 2.25 (m, 2H), 2.06 – 1.97 (m, 2H).

^{13}C NMR (101 MHz, CDCl$_3$) δ 173.3, 159.4 (d, *J* = 243.3 Hz), 136.4, 134.1 (d, *J* = 2.7 Hz), 131.4, 130.9, 128.9, 128.2, 126.6, 121.6 (d, *J* = 8.0 Hz), 115.7 (d, *J* = 22.5 Hz), 51.6, 30.5, 16.1.

^{19}F NMR (376 MHz, CDCl$_3$) δ -118.1 – -118.2 (m).

HRMS (**ESI**) (m/z): $[M + Na]^+$ Calcd for $C_{19}H_{18}FNONa$ 318.1264; Found: 318.1265.

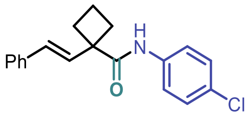

(E)-N-(4-chlorophenyl)-1-styrylcyclobutane-1-carboxamide (3g)

19.2 mg, 62% yield, white solid. Eluent (Pentane/Ethyl Acetate) = 15:1, $R_f = 0.2$.

^{1}H NMR (300 MHz, CDCl$_3$) δ 7.41 – 7.35 (m, 4H), 7.32 – 7.26 (m, 2H), 7.24 – 7.21 (m, 1H), 7.19 (d, *J* = 2.4 Hz, 1H), 7.18 – 7.13 (m, 2H), 6.62 (d, *J* = 16.1 Hz, 1H), 6.40 (d, *J* = 16.1 Hz, 1H), 2.71 – 2.58 (m, 2H), 2.27 – 2.16 (m, 2H), 2.00 – 1.87 (m, 2H).

^{13}C NMR (75 MHz, CDCl$_3$) δ 173.4, 136.7, 136.3, 131.2, 130.9, 129.2, 129.1, 128.9, 128.2, 126.6, 121.0, 51.7, 30.5, 16.1.

HRMS (**ESI**) (m/z): $[M + Na]^+$ Calcd for $C_{19}H_{18}ClNONa$ 334.0969; Found: 334.0966.

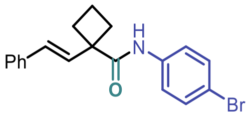

(E)-N-(4-bromophenyl)-1-styrylcyclobutane-1-carboxamide (3h)

26.1 mg, 74% yield, white solid. Eluent (Pentane/Ethyl Acetate) = 15:1, $R_f = 0.2$.

^{1}H NMR (300 MHz, CDCl$_3$) δ 7.47 – 7.42 (m, 2H), 7.40 (s, 4H), 7.37 – 7.31 (m, 2H), 7.30 – 7.26 (m, 1H), 7.24 (br, 1H), 6.70 (d, *J* = 16.1 Hz, 1H), 6.48 (d, *J* = 16.1 Hz, 1H), 2.78 – 2.67 (m, 2H), 2.34 – 2.23 (m, 2H), 2.08 – 1.94 (m, 2H).

^{13}C NMR (75 MHz, CDCl$_3$) δ 173.4, 137.2, 136.3, 132.0, 131.2, 131.0, 128.9, 128.2, 126.6, 121.4, 116.8, 51.7, 30.5, 16.1.

HRMS (**ESI**) (m/z): $[M + Na]^+$ Calcd for $C_{19}H_{18}BrNONa$ 378.0464; Found: 378.0465.

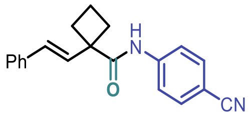

(E)-N-(4-cyanophenyl)-1-styrylcyclobutane-1-carboxamide (3i)
25.2 mg, 83% yield, white solid. Eluent (Pentane/Ethyl Acetate) = 5:1, R_f = 0.3.
^{1}H NMR (300 MHz, $CDCl_3$) δ 7.69 – 7.63 (m, 2H), 7.58 – 7.53 (m, 2H), 7.50 (br, 1H), 7.47 – 7.42 (m, 2H), 7.39 – 7.33 (m, 2H), 7.32 – 7.28 (m, 1H), 6.71 (d, *J* = 16.1 Hz, 1H), 6.48 (d, *J* = 16.1 Hz, 1H), 2.80 – 2.65 (m, 2H), 2.41 – 2.23 (m, 2H), 2.10 – 1.95 (m, 2H).
^{13}C NMR (75 MHz, $CDCl_3$) δ 173.8, 142.2, 136.1, 133.3, 131.3, 130.7, 128.9, 128.3, 126.6, 119.6, 119.0, 107.0, 51.9, 30.5, 16.0.
HRMS (**ESI**) (m/z): $[M + H]^+$ Calcd for $C_{20}H_{19}N_2O$ 303.1492; Found: 303.1493.

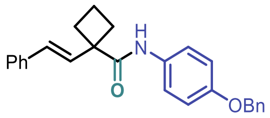

(E)-N-(4-(benzyloxy)phenyl)-1-styrylcyclobutane-1-carboxamide (3j)
19.4 mg, 51% yield, white solid. Eluent (Pentane/Ethyl Acetate) = 15:1, R_f = 0.3.
^{1}H NMR (300 MHz, $CDCl_3$) δ 7.48 – 7.38 (m, 7H), 7.38 – 7.32 (m, 4H), 7.31 – 7.28 (m, 1H), 7.12 (br, 1H), 6.96 – 6.88 (m, 2H), 6.69 (d, *J* = 16.1 Hz, 1H), 6.49 (d, *J* = 16.1 Hz, 1H), 5.04 (s, 2H), 2.81 – 2.65 (m, 2H), 2.35 – 2.22 (m, 2H), 2.09 – 1.95 (m, 2H).
^{13}C NMR (75 MHz, $CDCl_3$) δ 173.2, 155.6, 137.1, 136.5, 131.7, 131.5, 130.6, 128.9, 128.7, 128.1, 128.1, 127.6, 126.6, 121.6, 115.3, 70.4, 51.6, 30.6, 16.2.
HRMS (**ESI**) (m/z): $[M + Na]^+$ Calcd for $C_{26}H_{25}NO_2Na$ 406.1777; Found: 406.1786.

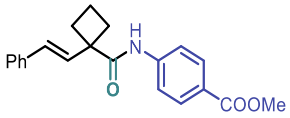

methyl (E)-4-(1-styrylcyclobutane-1-carboxamido)benzoate (3k)
25.6 mg, 76% yield, white solid. Eluent (Pentane/Ethyl Acetate) = 10:1, R_f = 0.2.
^{1}H NMR (300 MHz, $CDCl_3$) δ 7.94 – 7.87 (m, 2H), 7.56 – 7.48 (m, 2H), 7.41 – 7.33 (m, 3H), 7.31 – 7.25 (m, 2H), 7.24 – 7.19 (m, 1H), 6.63 (d, *J* = 16.1 Hz, 1H), 6.40 (d, *J* = 16.1 Hz, 1H), 3.80 (s, 3H), 2.72 – 2.59 (m, 2H), 2.27 – 2.16 (m, 2H), 2.00 – 1.89 (m, 2H).
^{13}C NMR (75 MHz, $CDCl_3$) δ 173.6, 166.7, 142.3, 136.3, 131.1, 131.0, 130.9, 128.9, 128.2, 126.6, 125.6, 118.8, 52.1, 51.9, 30.5, 16.1.
HRMS (**ESI**) (m/z): $[M + Na]^+$ Calcd for $C_{21}H_{21}NO_3Na$ 358.1414; Found: 385.1412.

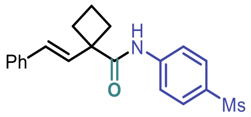

(E)-N-(4-(methylsulfonyl)phenyl)-1-styrylcyclobutane-1-carboxamide (3l)
13.3 mg, 37% yield, white solid. Eluent (Pentane/Ethyl Acetate) = 1:1, R_f = 0.2.
^{1}H NMR (300 MHz, $CDCl_3$) δ 7.89 – 7.83 (m, 2H), 7.76 – 7.70 (m, 2H), 7.53 (s, 1H), 7.48 – 7.43 (m, 2H), 7.40 – 7.33 (m, 2H), 7.33 – 7.27 (m, 1H), 6.72 (d, *J* = 16.1 Hz, 1H), 6.49 (d, *J* = 16.1 Hz, 1H), 3.01 (s, 3H), 2.81 – 2.65 (m, 2H), 2.38 – 2.25 (m, 2H), 2.10 – 1.99 (m, 2H).
^{13}C NMR (75 MHz, $CDCl_3$) δ 173.9, 143.0, 136.2, 135.3, 131.3, 130.7, 129.0, 128.8, 128.4, 126.6, 119.7, 51.9, 44.8, 30.5, 16.1.
HRMS (**ESI**) (m/z): $[M + H]^+$ Calcd for $C_{20}H_{22}NO_3S$ 356.1315; Found: 356.1316.

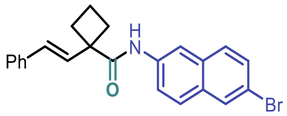

(E)-N-(6-bromonaphthalen-2-yl)-1-styrylcyclobutane-1-carboxamide (3m)

18.3 mg, 45% yield, white solid. Eluent (Pentane/Ethyl Acetate) = 10:1, $R_f = 0.3$.

^{1}H NMR (300 MHz, CDCl$_3$) δ 8.24 (d, $J = 2.1$ Hz, 1H), 7.91 (d, $J = 2.0$ Hz, 1H), 7.65 (dd, $J = 8.8, 6.4$ Hz, 2H), 7.53 – 7.45 (m, 3H), 7.44 – 7.34 (m, 4H), 7.34 – 7.28 (m, 1H), 6.75 (d, $J = 16.1$ Hz, 1H), 6.53 (d, $J = 16.1$ Hz, 1H), 2.84 – 2.72 (m, 2H), 2.38 – 2.28 (m, 2H), 2.13 – 1.99 (m, 2H).

^{13}C NMR (75 MHz, CDCl$_3$) δ 173.6, 136.4, 135.9, 132.4, 131.7, 131.3, 131.0, 130.0, 129.7, 129.4, 129.0, 128.3, 127.9, 126.6, 120.8, 118.8, 116.4, 51.8, 30.6, 16.2.

HRMS (**ESI**) (m/z): $[M + Na]^+$ Calcd for $C_{23}H_{20}BrNONa$ 428.0620; Found: 428.0628.

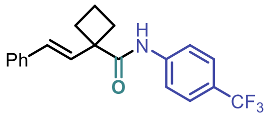

(E)-1-styryl-N-(4-(trifluoromethyl)phenyl)cyclobutane-1-carboxamide (3n)

27.4 mg, 79% yield, white solid. Eluent (Pentane/Ethyl Acetate) = 15:1, $R_f = 0.2$.

^{1}H NMR (400 MHz, CDCl$_3$) δ 7.64 (d, $J = 8.5$ Hz, 2H), 7.55 (d, $J = 8.7$ Hz, 2H), 7.47 – 7.44 (m, 2H), 7.40 – 7.35 (m, 3H), 7.32 – 7.28 (m, 1H), 6.72 (d, $J = 16.1$ Hz, 1H), 6.50 (d, $J = 16.1$ Hz, 1H), 2.78 – 2.69 (m, 2H), 2.31 (ddd, $J = 12.1, 8.4, 6.0$ Hz, 2H), 2.07 – 1.98 (m, 2H).

^{13}C NMR (101 MHz, CDCl$_3$) δ 173.7, 141.2, 136.3, 131.1, 131.0, 128.9, 128.3, 126.6, 126.3 (q, $J = 3.8$ Hz), 125.7 (d, $J = 32.4$ Hz), 122.9, 119.4, 51.8, 30.5, 16.1.

^{19}F NMR (376 MHz, CDCl$_3$) δ -62.06 (s).

HRMS (**ESI**) (m/z): $[M + H]^+$ Calcd for $C_{20}H_{19}F_3NO$ 346.1414; Found: 346.1413.

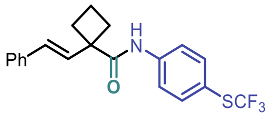

(E)-1-styryl-N-(4-((trifluoromethyl)thio)phenyl)cyclobutane-1-carboxamide (3o)

28.3 mg, 75% yield, white solid. Eluent (Pentane/Ethyl Acetate) = 15:1, $R_f = 0.3$.

^{1}H NMR (300 MHz, CDCl$_3$) δ 7.63 – 7.54 (m, 4H), 7.47 – 7.43 (m, 2H), 7.40 – 7.34 (m, 3H), 7.34 – 7.27 (m, 1H), 6.71 (d, $J = 16.1$ Hz, 1H), 6.48 (d, $J = 16.1$ Hz, 1H), 2.80 – 2.68 (m, 2H), 2.40 – 2.22 (m, 2H), 2.09 – 1.95 (m, 2H).

^{13}C NMR (75 MHz, CDCl$_3$) δ 173.6, 140.7, 137.6, 136.2, 131.1, 131.0, 129.6 (t, $J = 306.4$ Hz), 128.9, 128.3, 126.6, 120.2, 118.8 (d, $J = 2.3$ Hz), 51.8, 30.5, 16.1.

^{19}F NMR (282 MHz, CDCl$_3$) δ -43.35(s).

HRMS (**ESI**) (m/z): $[M + H]^+$ Calcd for $C_{20}H_{19}F_3NOS$ 378.1134; Found: 378.1136.

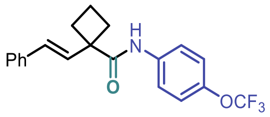

(E)-1-styryl-N-(4-(trifluoromethoxy)phenyl)cyclobutane-1-carboxamide (3p)

25.9 mg, 72% yield, white solid. Eluent (Pentane/Ethyl Acetate) = 10:1, $R_f = 0.3$.

^{1}H NMR (300 MHz, CDCl$_3$) δ 7.57 – 7.51 (m, 2H), 7.47 – 7.43 (m, 2H), 7.40 – 7.33 (m, 2H), 7.32 – 7.26 (m, 2H), 7.18 – 7.12 (m, 2H), 6.71 (d, $J = 16.1$ Hz, 1H), 6.49 (d, $J = 16.1$ Hz, 1H), 2.82 – 2.66 (m, 2H), 2.37 – 2.21 (m, 2H), 2.11 – 1.94 (m, 2H).

^{13}C NMR (75 MHz, CDCl$_3$) δ 173.4, 145.2, 136.7, 136.2, 131.1, 130.9, 128.8, 128.1, 126.5, 121.7, 120.8, 51.6, 30.4, 16.0.

^{19}F NMR (282 MHz, CDCl$_3$) δ -58.15 (s).
HRMS (ESI) (m/z): $[M + H]^+$ Calcd for $C_{20}H_{19}F_3NO_2$ 362.1363; Found: 362.1373.

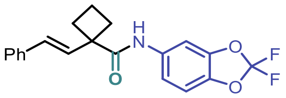

(E)-N-(2,2-difluorobenzo[d][1,3]dioxol-5-yl)-1-styrylcyclobutane-1-carboxamide (3q)
25.7 mg, 72% yield, white solid. Eluent (Pentane/Ethyl Acetate) = 10:1, R_f = 0.3.
^{1}H NMR (300 MHz, CDCl$_3$) δ 7.57 – 7.54 (m, 1H), 7.40 – 7.35 (m, 2H), 7.32 – 7.26 (m, 2H), 7.25 – 7.19 (m, 2H), 6.88 – 6.81 (m, 2H), 6.62 (d, *J* = 16.1 Hz, 1H), 6.40 (d, *J* = 16.1 Hz, 1H), 2.71 – 2.59 (m, 2H), 2.27 – 2.16 (m, 2H), 2.03 – 1.86 (m, 2H).
^{13}C NMR (75 MHz, CDCl$_3$) δ 173.5, 144.0, 140.2, 136.3, 134.3, 131.9, 131.1, 128.9, 128.3, 126.6, 114.6, 109.3, 103.2, 51.6, 30.5, 16.1.
^{19}F NMR (282 MHz, CDCl$_3$) δ -50.01 (s).
HRMS (ESI) (m/z): $[M + H]^+$ Calcd for $C_{20}H_{18}F_2NO_3$ 358.1249; Found: 358.1257.

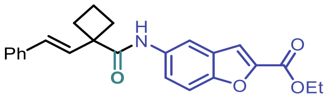

ethyl (E)-5-(1-styrylcyclobutane-1-carboxamido)benzofuran-2-carboxylate (3r)
15.0 mg, 38% yield, white solid. Eluent (Pentane/Ethyl Acetate) = 5:1, R_f = 0.3.
^{1}H NMR (300 MHz, CDCl$_3$) δ 8.09 (d, *J* = 2.1 Hz, 1H), 7.51 – 7.44 (m, 4H), 7.40 – 7.33 (m, 3H), 7.30 (td, *J* = 3.2, 2.0 Hz, 1H), 6.72 (d, *J* = 16.1 Hz, 1H), 6.52 (d, *J* = 16.1 Hz, 1H), 4.43 (q, *J* = 7.1 Hz, 2H), 2.82 – 2.69 (m, 2H), 2.37 – 2.24 (m, 2H), 2.11 – 1.99 (m, 2H), 1.42 (t, *J* = 7.1 Hz, 3H).
^{13}C NMR (75 MHz, CDCl$_3$) δ 173.4, 159.5, 152.6, 146.6, 136.3, 134.1, 131.3, 130.8, 128.8, 128.1, 127.4, 126.5, 120.8, 113.9, 113.6, 112.5, 61.6, 51.6, 30.5, 16.1, 14.3.
HRMS (ESI) (m/z): $[M + Na]^+$ Calcd for $C_{24}H_{23}NO_4Na$ 412.1519; Found: 412.1523.

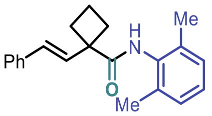

(E)-N-(2,6-dimethylphenyl)-1-styrylcyclobutane-1-carboxamide (3s)
23.0 mg, 75% yield, white solid. Eluent (Pentane/Ethyl Acetate) = 10:1, R_f = 0.2.
^{1}H NMR (400 MHz, CDCl$_3$) δ 7.48 – 7.43 (m, 2H), 7.39 – 7.34 (m, 2H), 7.31 – 7.26 (m, 1H), 7.11 – 7.03 (m, 3H), 6.83 (br, 1H), 6.75 (d, *J* = 16.1 Hz, 1H), 6.58 (d, *J* = 16.1 Hz, 1H), 2.82 – 2.72 (m, 2H), 2.38 – 2.29 (m, 2H), 2.19 (s, 6H), 2.09 – 1.98 (m, 2H).
^{13}C NMR (101 MHz, CDCl$_3$) δ 173.4, 136.6, 135.4, 133.9, 132.1, 130.5, 128.9, 128.3, 128.0, 127.3, 126.5, 51.4, 30.6, 18.5, 16.4.
HRMS (ESI) (m/z): $[M + Na]^+$ Calcd for $C_{21}H_{23}NONa$ 328.1672; Found: 328.1674.

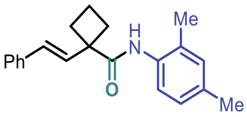

(E)-N-(2,4-dimethylphenyl)-1-styrylcyclobutane-1-carboxamide (3t)
23.7 mg, 78% yield, white solid. Eluent (Pentane/Ethyl Acetate) = 15:1, R_f = 0.3.
^{1}H NMR (400 MHz, CDCl$_3$) δ 7.76 (d, *J* = 8.2 Hz, 1H), 7.47 – 7.43 (m, 2H), 7.40 – 7.33 (m, 2H), 7.31 – 7.26 (m, 1H), 7.15 (br, 1H), 7.01 (dd, *J* = 8.1, 2.1 Hz, 1H), 6.95 (d, *J* = 2.0 Hz, 1H), 6.73 (d, *J* = 16.1 Hz, 1H), 6.54 (d, *J* = 16.1 Hz, 1H), 2.80 – 2.71 (m, 2H), 2.34 – 2.25 (m, 5H), 2.09 (s, 3H), 2.07 – 1.96 (m, 2H).

^{13}C NMR (101 MHz, CDCl$_3$) δ 173.1, 136.5, 134.6, 133.4, 132.1, 131.2, 130.8, 128.9, 128.5, 128.1, 127.5, 126.5, 122.5, 51.7, 30.6, 21.0, 17.7, 16.3.
HRMS (**ESI**) (m/z): $[M + Na]^+$ Calcd for $C_{21}H_{23}NONa$ 328.1672; Found: 328.1679.

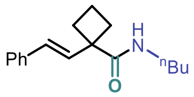

(E)-N-butyl-1-styrylcyclobutane-1-carboxamide (3u)
12.4 mg, 48% yield, white solid. Eluent (Pentane/Ethyl Acetate) = 10:1, R_f = 0.3.
^{1}H NMR (300 MHz, CDCl$_3$) δ 7.44 – 7.39 (m, 2H), 7.37 – 7.31 (m, 2H), 7.29 – 7.23 (m, 1H), 6.58 (d, *J* = 16.1 Hz, 1H), 6.40 (d, *J* = 16.1 Hz, 1H), 5.50 (br, 1H), 3.23 (td, *J* = 7.1, 5.8 Hz, 2H), 2.69 – 2.54 (m, 2H), 2.26 – 2.12 (m, 2H), 2.03 – 1.87 (m, 2H), 1.51 – 1.40 (m, 2H), 1.37 – 1.24 (m, 2H), 0.90 (t, *J* = 7.2 Hz, 3H).
^{13}C NMR (75 MHz, CDCl$_3$) δ 175.2, 136.8, 132.3, 129.8, 128.8, 127.9, 126.5, 50.8, 39.6, 31.9, 30.6, 20.2, 16.2, 13.9.
HRMS (**ESI**) (m/z): $[M + Na]^+$ Calcd for $C_{17}H_{23}NONa$ 280.1672; Found: 280.1678.

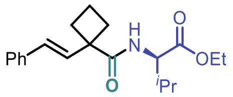

ethyl (E)-(1-styrylcyclobutane-1-carbonyl)-D-valinate (3v)
14.4 mg, 44% yield, colorless oil. Eluent (Pentane/Ethyl Acetate) = 10:1, R_f = 0.3.
^{1}H NMR (300 MHz, CDCl$_3$) δ 7.43 – 7.39 (m, 2H), 7.37 – 7.30 (m, 2H), 7.28 – 7.22 (m, 1H), 6.63 (d, *J* = 16.1 Hz, 1H), 6.43 (d, *J* = 16.1 Hz, 1H), 6.00 (d, *J* = 8.8 Hz, 1H), 4.53 (dd, *J* = 8.8, 4.8 Hz, 1H), 4.17 (qd, *J* = 7.2, 4.6 Hz, 2H), 2.68 – 2.57 (m, 2H), 2.30 – 2.10 (m, 3H), 2.03 – 1.90 (m, 2H), 1.25 (t, *J* = 7.1 Hz, 3H), 0.92 (d, *J* = 6.9 Hz, 3H), 0.85 (d, *J* = 6.9 Hz, 3H).
^{13}C NMR (75 MHz, CDCl$_3$) δ 175.3, 172.1, 136.8, 132.0, 130.1, 128.8, 127.8, 126.5, 61.3, 57.2, 51.0, 31.5, 30.8, 30.6, 19.1, 17.8, 16.2, 14.3.
HRMS (**ESI**) (m/z): $[M + Na]^+$ Calcd for $C_{20}H_{27}NO_3Na$ 352.1883; Found: 352.1881.

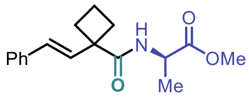

methyl (E)-(1-styrylcyclobutane-1-carbonyl)-D-alaninate (3w)
15.7 mg, 55% yield, colorless oil. Eluent (Pentane/Ethyl Acetate) = 7:1, R_f = 0.3.
^{1}H NMR (300 MHz, CDCl$_3$) δ 7.45 – 7.40 (m, 2H), 7.37 – 7.31 (m, 2H), 7.29 – 7.22 (m, 1H), 6.62 (d, *J* = 16.1 Hz, 1H), 6.41 (dd, *J* = 16.1, 13.9 Hz, 1H), 6.04 (dd, *J* = 443.9, 7.5 Hz, 1H), 4.58 (p, *J* = 7.2 Hz, 1H), 3.73 (s, 3H), 2.67 – 2.56 (m, 2H), 2.30 – 2.15 (m, 2H), 2.04 – 1.90 (m, 2H), 1.38 (d, *J* = 7.2 Hz, 3H).
^{13}C NMR (75 MHz, CDCl$_3$) δ 175.0, 173.7, 136.8, 131.7, 130.2, 128.8, 127.9, 126.5, 52.6, 50.7, 48.3, 30.6, 30.6, 18.5, 16.1.
HRMS (**ESI**) (m/z): $[M + Na]^+$ Calcd for $C_{17}H_{21}NO_3Na$ 310.1413; Found: 310.1413.

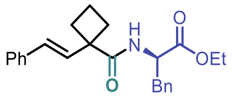

ethyl (E)-(1-styrylcyclobutane-1-carbonyl)-D-phenylalaninate (3x)
12.8 m, 34% yield, white solid. Eluent (Pentane/Ethyl Acetate) = 10:1, R_f = 0.2.
^{1}H NMR (300 MHz, CDCl$_3$) δ 7.39 – 7.31 (m, 4H), 7.29 – 7.23 (m, 1H), 7.18 – 7.08 (m, 3H), 7.06 – 7.01 (m, 2H), 6.52 (d, *J* = 16.1 Hz, 1H), 6.30 (d, *J* = 16.1 Hz, 1H), 5.94 (d, *J* = 7.9 Hz, 1H), 4.83 (dt, *J* = 7.9, 5.9 Hz, 1H), 4.16 (q, *J* = 7.2 Hz, 2H), 3.09 (qd, *J* = 13.8, 5.9 Hz, 2H), 2.64 – 2.47 (m, 2H), 2.28 – 2.06 (m, 2H), 1.98 – 1.88 (m, 2H), 1.23 (t, *J* = 7.2 Hz, 3H).
^{13}C NMR (75 MHz, CDCl$_3$) δ 174.8, 171.6, 136.7, 135.9, 131.8, 130.2, 129.4, 128.7, 128.6, 127.9, 127.2, 126.5, 61.6, 53.1, 50.7, 37.9, 30.5 (d, *J* = 2.6 Hz), 16.2, 14.2.

HRMS (**ESI**) (m/z): $[M + Na]^+$ Calcd for $C_{24}H_{27}NO_3Na$ 400.1883; Found: 400.1871.

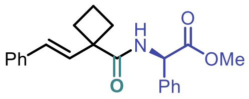

methyl (R,E)-2-phenyl-2-(1-styrylcyclobutane-1-carboxamido)acetate (3y)
12.7 mg, 35% yield, colorless oil. Eluent (Pentane/Ethyl Acetate) = 10:1, $R_f = 0.2$.
^{1}H NMR (300 MHz, CDCl$_3$) δ 7.42 – 7.37 (m, 2H), 7.37 – 7.30 (m, 7H), 7.28 – 7.22 (m, 1H), 6.60 (d, *J* = 16.1 Hz, 1H), 6.50 (d, *J* = 7.1 Hz, 1H), 6.42 (d, *J* = 16.1 Hz, 1H), 5.56 (d, *J* = 7.1 Hz, 1H), 3.71 (s, 3H), 2.71 – 2.54 (m, 2H), 2.30 – 2.15 (m, 2H), 2.01 – 1.90 (m, 2H).
^{13}C NMR (75 MHz, CDCl$_3$) δ 174.8, 171.5, 136.8, 136.7, 131.7, 130.2, 129.1, 128.8, 128.7, 127.9, 127.3, 126.5, 56.7, 52.9, 50.7, 30.7, 16.1.
HRMS (**ESI**) (m/z): $[M + Na]^+$ Calcd for $C_{22}H_{23}NO_3Na$ 372.1570; Found: 372.1575.

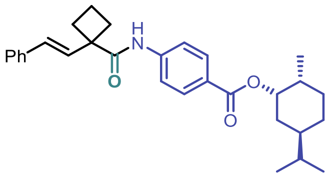

(1S,2R,5R)-5-isopropyl-2-methylcyclohexyl 4-(1-((E)-styryl)cyclobutane-1-carboxamido)benzoate (3z)
38.3 mg, 83% yield, white solid. Eluent (Pentane/Ethyl Acetate) = 15:1, $R_f = 0.2$.
^{1}H NMR (300 MHz, CDCl$_3$) δ 7.96 – 7.83 (m, 2H), 7.56 – 7.47 (m, 2H), 7.39 – 7.35 (m, 2H), 7.33 (br, 1H), 7.32 – 7.25 (m, 2H), 7.24 – 7.19 (m, 1H), 6.63 (d, *J* = 16.1 Hz, 1H), 6.41 (d, *J* = 16.1 Hz, 1H), 4.90 – 4.79 (m, 1H), 2.75 – 2.57 (m, 2H), 2.28 – 2.17 (m, 2H), 2.08 – 1.80 (m, 4H), 1.69 – 1.60 (m, 3H), 1.54 – 1.42 (m, 2H), 1.10 – 0.97 (m, 2H), 0.83 (dd, *J* = 6.8, 4.4 Hz, 6H), 0.70 (d, *J* = 6.9 Hz, 3H).
^{13}C NMR (75 MHz, CDCl$_3$) δ 173.6, 165.7, 142.1, 136.3, 131.1, 131.0, 130.9, 128.9, 128.2, 126.6, 126.3, 118.8, 74.8, 51.9, 47.4, 41.1, 34.4, 31.6, 30.5, 26.7, 23.8, 22.2, 20.9, 16.7, 16.1.
HRMS (**ESI**) (m/z): $[M + Na]^+$ Calcd for $C_{30}H_{37}NO_3Na$ 482.2665; Found: 482.2678.

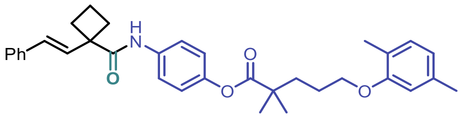

(E)-4-(1-styrylcyclobutane-1-carboxamido)phenyl 5-(2,5-dimethylphenoxy)-2,2-dimethylpentanoate (3ab)
31.9 mg, 61% yield, white solid. Eluent (Pentane/Ethyl Acetate) = 10:1, $R_f = 0.3$.
^{1}H NMR (300 MHz, CDCl$_3$) δ 7.54 – 7.44 (m, 4H), 7.40 – 7.34 (m, 2H), 7.33 – 7.28 (m, 1H), 7.25 (br, 1H), 7.01 (d, *J* = 8.0 Hz, 1H), 6.99 – 6.94 (m, 2H), 6.74 – 6.62 (m, 3H), 6.50 (d, *J* = 16.1 Hz, 1H), 3.98 (q, *J* = 3.0 Hz, 2H), 2.80 – 2.68 (m, 2H), 2.34 – 2.24 (m, 5H), 2.18 (s, 3H), 2.07 – 1.96 (m, 2H), 1.88 (d, *J* = 2.9 Hz, 4H), 1.36 (s, 6H).
^{13}C NMR (75 MHz, CDCl$_3$) δ 176.5, 173.3, 157.0, 147.2, 136.6, 136.4, 135.7, 131.4, 130.8, 130.5, 128.9, 128.2, 126.6, 123.7, 122.0, 120.9, 120.7, 112.1, 67.9, 51.7, 42.5, 37.3, 30.5, 25.4, 25.3, 21.5, 16.1, 15.9.
HRMS (**ESI**) (m/z): $[M + Na]^+$ Calcd for $C_{34}H_{39}NO_4Na$ 548.2771; Found: 548.2776.

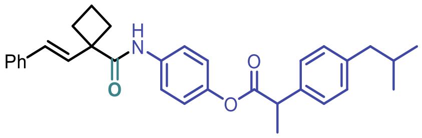

(E)-4-(1-styrylcyclobutane-1-carboxamido)phenyl 2-(4-isobutylphenyl)propanoate (3ac)
28.2 mg, 59% yield, white solid. Eluent (Pentane/Ethyl Acetate) = 10:1, $R_f = 0.2$.
^{1}H NMR (300 MHz, CDCl$_3$) δ 7.50 – 7.41 (m, 4H), 7.39 – 7.33 (m, 2H), 7.31 – 7.27 (m, 3H), 7.21 (br, 1H), 7.16 – 7.11 (m, 2H), 6.96 – 6.90 (m, 2H), 6.69 (d, *J* = 16.1 Hz, 1H), 6.48 (d, *J* = 16.1 Hz, 1H), 3.91 (q, *J* = 7.1

Hz, 1H), 2.78 – 2.67 (m, 2H), 2.47 (d, J = 7.2 Hz, 2H), 2.32 – 2.23 (m, 2H), 2.06 – 1.95 (m, 2H), 1.86 (dt, J = 13.5, 6.8 Hz, 1H), 1.59 (d, J = 7.2 Hz, 3H), 0.91 (d, J = 6.6 Hz, 6H).
^{13}C NMR (75 MHz, CDCl$_3$) δ 173.4, 173.3, 147.1, 141.0, 137.3, 136.4, 135.7, 131.4, 130.8, 129.6, 128.9, 128.2, 127.3, 126.6, 121.9, 120.6, 51.7, 45.3, 45.2, 30.5, 30.3, 22.5, 18.7, 16.1.
HRMS (**ESI**) (m/z): [M + Na]$^+$ Calcd for $C_{32}H_{35}NO_3Na$ 504.2509; Found: 504.2520.

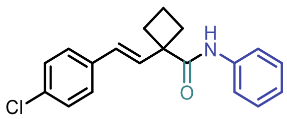

(E)-1-(4-chlorostyryl)-N-phenylcyclobutane-1-carboxamide (3ad)

17.4 mg, 56% yield, white solid. Eluent (Pentane/Ethyl Acetate) = 10:1, R_f = 0.2.

^{1}H NMR (400 MHz, CDCl$_3$) δ 7.50 (dd, J = 8.6, 1.2 Hz, 2H), 7.39 – 7.35 (m, 2H), 7.34 – 7.28 (m, 4H), 7.16 (br, 1H), 7.12 – 7.07 (m, 1H), 6.64 (d, J = 16.1 Hz, 1H), 6.47 (d, J = 16.1 Hz, 1H), 2.77 – 2.69 (m, 2H), 2.28 (ddd, J = 11.8, 8.6, 5.7 Hz, 2H), 2.07 – 1.97 (m, 2H).
^{13}C NMR (101 MHz, CDCl$_3$) δ 173.1, 138.0, 135.0, 133.8, 132.3, 129.4, 129.1, 129.0, 127.8, 124.4, 119.8, 51.8, 30.6, 16.0.
HRMS (**ESI**) (m/z): [M + Na]$^+$ Calcd for $C_{19}H_{18}ClNONa$ 334.0969; Found: 334.0975.

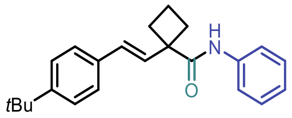

(E)-1-(4-(tert-butyl)styryl)-N-phenylcyclobutane-1-carboxamide (3ae)

25.5 mg, 77% yield, white solid. Eluent (Pentane/Ethyl Acetate) = 20:1, R_f = 0.2.

^{1}H NMR (400 MHz, CDCl$_3$) δ 7.53 – 7.47 (m, 2H), 7.40 (s, 4H), 7.33 – 7.27 (m, 2H), 7.23 (br, 1H), 7.11 – 7.05 (m, 1H), 6.70 (d, J = 16.1 Hz, 1H), 6.47 (d, J = 16.1 Hz, 1H), 2.80 – 2.68 (m, 2H), 2.27 (ddd, J = 11.9, 8.8, 5.4 Hz, 2H), 2.09 – 1.93 (m, 2H), 1.34 (s, 9H).
^{13}C NMR (101 MHz, CDCl$_3$) δ 173.4, 151.4, 138.1, 133.7, 130.7, 130.6, 129.1, 126.3, 125.8, 124.3, 119.8, 51.7, 34.8, 31.4, 30.5, 16.2.
HRMS (**ESI**) (m/z): [M + Na]$^+$ Calcd for $C_{23}H_{27}NONa$ 356.1985; Found: 356.1984.

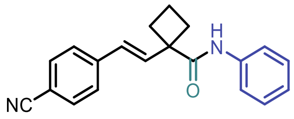

(E)-1-(4-cyanostyryl)-N-phenylcyclobutane-1-carboxamide (3af)

20.2 mg, 67% yield, white solid. Eluent (Pentane/Ethyl Acetate) = 5:1, R_f = 0.3.

^{1}H NMR (400 MHz, CDCl$_3$) δ 7.64 – 7.61 (m, 2H), 7.53 – 7.48 (m, 4H), 7.34 – 7.29 (m, 2H), 7.15 – 7.07 (m, 2H), 6.71 – 6.60 (m, 2H), 2.79 – 2.70 (m, 2H), 2.35 – 2.27 (m, 2H), 2.07 – 1.96 (m, 2H).
^{13}C NMR (101 MHz, CDCl$_3$) δ 172.6, 140.9, 137.8, 135.6, 132.6, 129.1, 128.7, 126.9, 124.5, 119.7, 118.8, 111.2, 51.9, 30.5, 15.8.
HRMS (**ESI**) (m/z): [M + H]$^+$ Calcd for $C_{20}H_{19}N_2O$ 303.1492; Found: 303.1500.

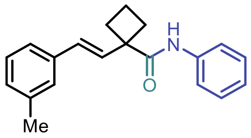

(E)-1-(3-methylstyryl)-N-phenylcyclobutane-1-carboxamide (3ag)

20.8 mg, 71% yield, white solid. Eluent (Pentane/Ethyl Acetate) = 15:1, R_f = 0.3.

^{1}H NMR (300 MHz, CDCl$_3$) δ 7.54 – 7.48 (m, 2H), 7.33 – 7.27 (m, 4H), 7.25 (d, J = 1.0 Hz, 1H), 7.22 (br, 1H), 7.13 – 7.05 (m, 2H), 6.68 (d, J = 16.1 Hz, 1H), 6.49 (d, J = 16.1 Hz, 1H), 2.78 – 2.66 (m, 2H), 2.37 (t, J = 0.6 Hz, 3H), 2.33 – 2.23 (m, 2H), 2.09 – 1.93 (m, 2H).

^{13}C NMR (75 MHz, CDCl$_3$) δ 173.4, 138.5, 138.1, 136.4, 131.3, 130.8, 129.1, 128.9, 128.8, 127.3, 124.3, 123.8, 119.8, 51.7, 30.5, 21.5, 16.1.
HRMS (**ESI**) (m/z): [M + Na]$^+$ Calcd for $C_{20}H_{21}NONa$ 314.1515; Found: 314.1522.

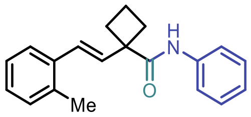

(E)-1-(2-methylstyryl)-N-phenylcyclobutane-1-carboxamide (3ah)
18.1 mg, 62% yield, white solid. Eluent (Pentane/Ethyl Acetate) = 15:1, R_f = 0.3.
^{1}H NMR (400 MHz, CDCl$_3$) δ 7.53 – 7.48 (m, 3H), 7.34 – 7.28 (m, 2H), 7.23 – 7.18 (m, 3H), 7.12 – 7.07 (m, 1H), 6.93 (d, *J* = 16.0 Hz, 1H), 6.38 (d, *J* = 16.0 Hz, 1H), 2.81 – 2.70 (m, 2H), 2.40 (s, 3H), 2.35 – 2.24 (m, 2H), 2.09 – 1.97 (m, 2H).
^{13}C NMR (101 MHz, CDCl$_3$) δ 173.3, 138.1, 135.6, 135.6, 133.2, 130.6, 129.1, 128.8, 128.0, 126.5, 125.8, 124.3, 119.7, 52.0, 30.7, 20.0, 16.2.
HRMS (**ESI**) (m/z): [M + Na]$^+$ Calcd for $C_{20}H_{21}NONa$ 314.1515; Found: 314.1511.

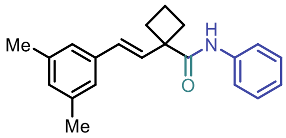

(E)-1-(3,5-dimethylstyryl)-N-phenylcyclobutane-1-carboxamide (3ai)
24.2 mg, 79% yield, white solid. Eluent (Pentane/Ethyl Acetate) = 30:1, R_f = 0.3.
^{1}H NMR (400 MHz, CDCl$_3$) δ 7.53 – 7.48 (m, 2H), 7.33 – 7.28 (m, 2H), 7.23 (br, 1H), 7.12 – 7.06 (m, 3H), 6.97 – 6.91 (m, 1H), 6.65 (d, *J* = 16.0 Hz, 1H), 6.48 (d, *J* = 16.1 Hz, 1H), 2.81 – 2.65 (m, 2H), 2.34 (d, *J* = 0.7 Hz, 6H), 2.32 – 2.23 (m, 2H), 2.08 – 1.93 (m, 2H).
^{13}C NMR (101 MHz, CDCl$_3$) δ 173.4, 138.4, 138.1, 136.4, 131.1, 130.9, 129.8, 129.1, 124.5, 124.3, 119.7, 51.7, 30.5, 21.4, 16.1.
HRMS (**ESI**) (m/z): [M + Na]$^+$ Calcd for $C_{21}H_{23}NONa$ 328.1672; Found: 328.1674.

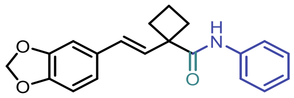

(E)-1-(2-(benzo[d][1,3]dioxol-5-yl)vinyl)-N-phenylcyclobutane-1-carboxamide (3aj)
17.7 mg, 60% yield, white solid. Eluent (Pentane/Ethyl Acetate) = 10:1, R_f = 0.3.
^{1}H NMR (400 MHz, CDCl$_3$) δ 7.52 – 7.48 (m, 2H), 7.33 – 7.27 (m, 2H), 7.21 (br, 1H), 7.11 – 7.06 (m, 1H), 6.98 (d, *J* = 1.7 Hz, 1H), 6.87 (ddd, *J* = 8.0, 1.8, 0.5 Hz, 1H), 6.81 – 6.76 (m, 1H), 6.60 (d, *J* = 16.1 Hz, 1H), 6.31 (d, *J* = 16.1 Hz, 1H), 5.97 (s, 2H), 2.78 – 2.67 (m, 2H), 2.29 – 2.22 (m, 2H), 2.06 – 1.94 (m, 2H).
^{13}C NMR (101 MHz, CDCl$_3$) δ 173.5, 148.3, 147.7, 138.1, 130.9, 130.3, 129.7, 129.1, 124.3, 121.4, 119.7, 108.5, 105.8, 101.3, 51.6, 30.6, 16.1.
HRMS (**ESI**) (m/z): [M + Na]$^+$ Calcd for $C_{20}H_{19}NO_3Na$ 344.1257; Found: 344.1262.

6.Synthetic transformations of 3a

6.1 Epoxidation of compound 3a

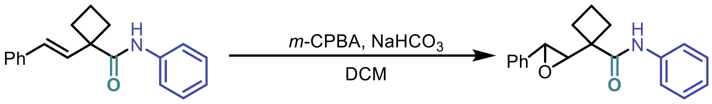

3a **4**, 63%

To a stirred solution of alkene **3a** (0.1 mmol, 1.0 equiv.) in DCM was added *m*-CPBA (0.15 mmol, 1.5 equiv.) and $NaHCO_3$ (0.12 mmol, 1.2 equiv.) at room temperature. The reaction mixture was stirred overnight before a saturated aqueous solution of Na_2SO_3 was added. The aqueous phase was extracted with ethyl acetate (3 × 5 mL). The combined organic layers were dried over Na_2SO_4, filtered, and concentrated under reduced pressure. The crude product was purified by column chromatography (Pentane/Ethyl Acetate = 8:1 R_f = 0.2) to afford the corresponding product **N-phenyl-1-(3-phenyloxiran-2-yl)cyclobutane-1-carboxamide (4)** (18.4 mg, 63%, white solid).

^{1}H NMR (300 MHz, $CDCl_3$) δ 7.70 (br, 1H), 7.59 – 7.53 (m, 2H), 7.40 – 7.35 (m, 3H), 7.35 – 7.29 (m, 4H), 7.15 – 7.06 (m, 1H), 4.01 (d, *J* = 2.2 Hz, 1H), 3.37 (d, *J* = 2.2 Hz, 1H), 2.74 – 2.63 (m, 1H), 2.62 – 2.53 (m, 1H), 2.23 – 2.14 (m, 1H), 2.12 – 1.98 (m, 3H).
^{13}C NMR (75 MHz, $CDCl_3$) δ 171.9, 138.1, 136.2, 129.1, 128.8, 128.8, 125.8, 124.4, 120.0, 65.0, 57.2, 48.2, 27.2, 25.9, 16.1.
HRMS (**ESI**) (m/z): $[M + Na]^+$ Calcd for $C_{19}H_{19}NO_2Na$ 316.1308; Found: 316.1315.

7. Synthesis of 5 and transformation

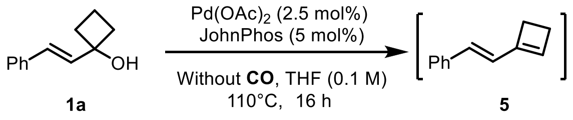

A vial (4 mL) was charged with $Pd(OAc)_2$ (2.5 mol%, 0.6 mg), JohnPhos (5 mol%, 1.5 mg), cyclobutanol (1.0 equiv., 0.1 mmol,), aniline hydrochloride (1.0 equiv., 0.1 mmol) and a stirring bar. The vial was closed by PTFE/white rubber septum (Wheaton 13 mm Septa) and phenolic cap and connected with atmosphere with a needle. The vial was evacuated under vacuum and recharged with argon for three times. After that, THF (1.0 mL) were injected under argon by using a syringe. Subsequently, the vial (or several vials) was placed in an alloy plate, which was transferred into a 300 mL autoclave of the 4560 series from Parr Instruments. Then, the reaction was performed for 16 h at 110 °C (oil bath). After reaction, cooling to room temperature. The crude product filtrated and evaporated to afford the conjugated diene **5** (8.3 mg, 53%, colorless oil).

This intermediate was not stable, and we judge it based on GC-MS.

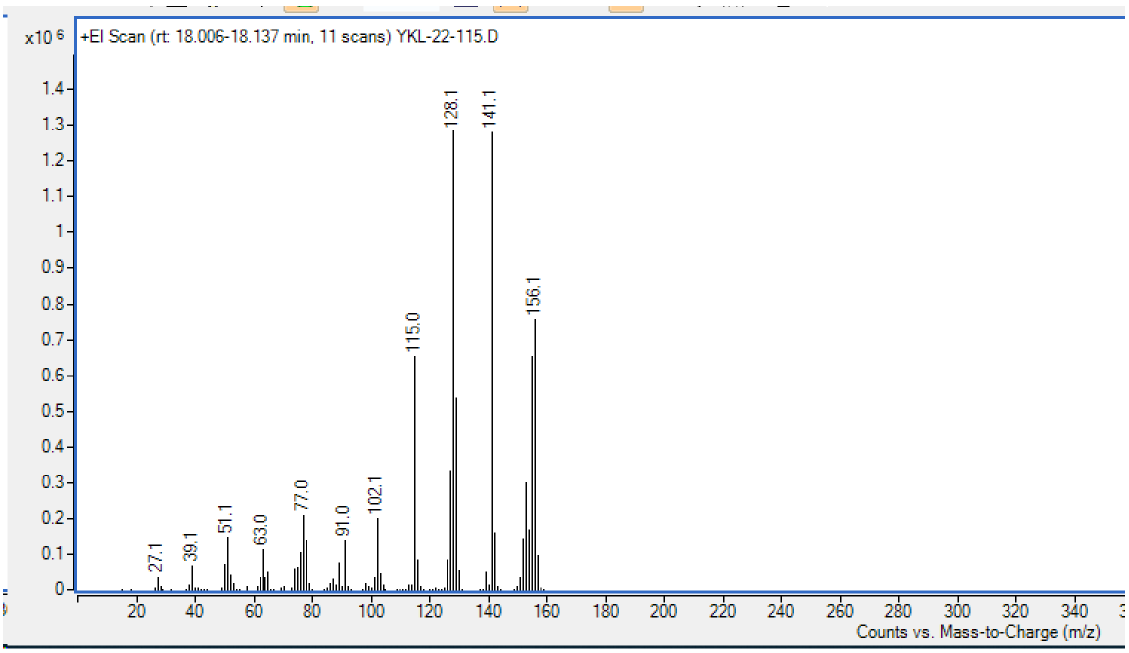

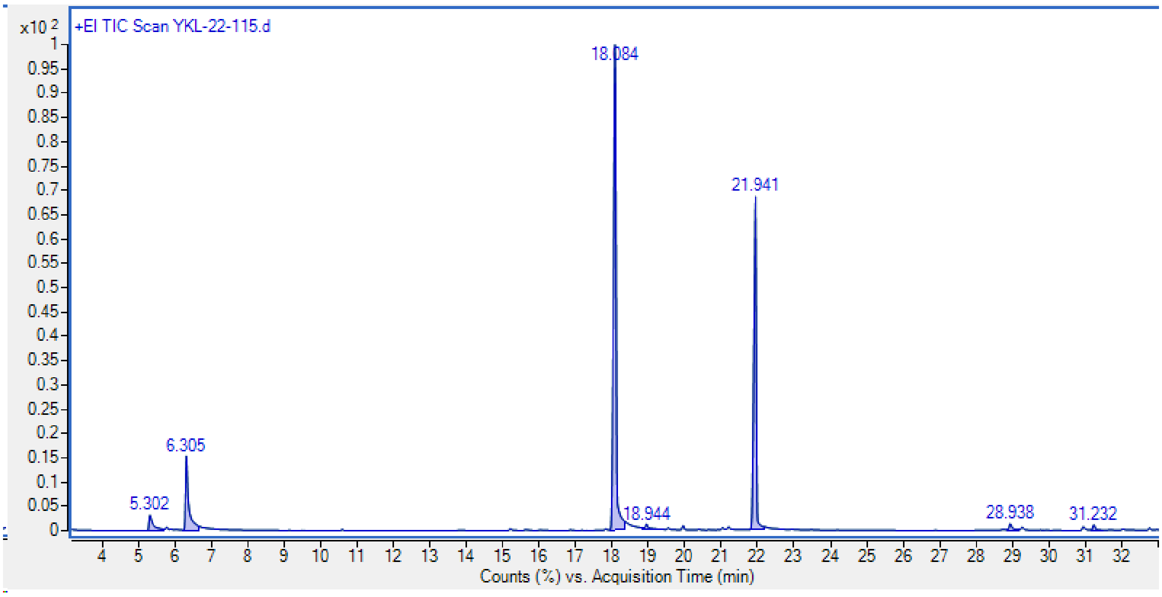

Intermediate verification experiment

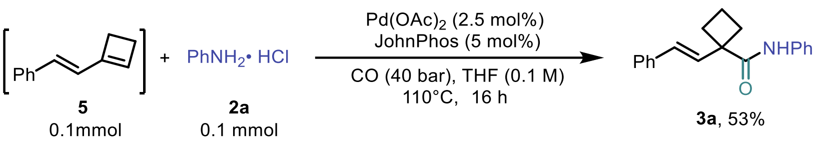

A vial (4 mL) was charged with $Pd(OAc)_2$ (2.5 mol%, 0.6 mg), JohnPhos (5 mol%, 1.5 mg), conjugated diene (1.0 equiv., 0.1 mmol, 15.6 mg), amine hydrochloride (1.0 equiv., 0.1 mmol, 12.9 mg) and a stirring bar. The vial was closed by PTFE/white rubber septum (Wheaton 13 mm Septa) and phenolic cap and connected with atmosphere with a needle. The vial was evacuated under vacuum and recharged with argon for three times. After that, THF (1.0 mL) were injected under argon by using a syringe. Subsequently, the vial (or several vials) was placed in an alloy plate, which was transferred into a 300 mL autoclave of the 4560 series from Parr Instruments. After flushing the autoclave three times with CO, a pressure of 40 bar of CO was adjusted at ambient temperature. Then, the reaction was performed for 16 h at 110 °C (aluminum block). After 16 hours, the autoclave was cooled down with ice water to room temperature and the pressure was released carefully. The solution was concentrated in vacuo then purified by silica-gel column chromatography using pentane and ethyl acetate to afford the corresponding product **3a** (14.7 mg, 53%, white solid).

8. X-ray Crystallographic Data of Product 3a (ellipsoid contours of probability levels are 50%) prepared from EA at room temperature

Data were collected on a Bruker Kappa APEX II Duo diffractometer. The structure was solved by direct methods (SHELXS-97: Sheldrick, G. M. Acta Cryst. 2008, A64, 112.) and refined by full-matrix least-squares procedures on F 2 (SHELXL-2019: Sheldrick, G. M. Acta Cryst. 2015, C71, 3.). XP (Bruker AXS) was used for graphical representations.

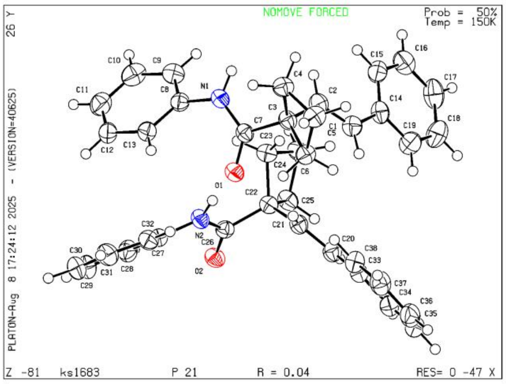

Figure 1. ORTEP drawing of product **3a** (CCDC 2474517)

Bond precision:	C-C = 0.0035 A		Wavelength=0.71073
Cell:	a=8.4693(2)	b=10.9659(2)	c=16.6100(5)
	alpha=90	beta=91.779(2)	gamma=90
Temperature:	150 K		

	Calculated	Reported
Volume	1541.89(7)	1541.89(7)
Space group	P 21	P 21
Hall group	P 2yb	P 2yb
Moiety formula	C19 H19 N O	?
Sum formula	C19 H19 N O	C19 H19 N O
Mr	277.35	277.35
Dx,g cm-3	1.195	1.195
Z	4	4
Mu (mm-1)	0.073	0.073
F000	592.0	592.0
F000'	592.23	
h,k,lmax	11,14,21	11,14,21
Nref	7442[3913]	7441
Tmin,Tmax	0.987,0.997	0.980,0.990
Tmin'	0.983	

Correction method= # Reported T Limits: Tmin=0.980 Tmax=0.990
AbsCorr = MULTI-SCAN

Data completeness= 1.90/1.00 Theta(max)= 27.994

R(reflections)= 0.0441(6648) wR2(reflections)= 0.1186(7441)

S = 1.069 Npar= 387

9.References

(1) Zhang, R.-Y.; Xi, L.-Y.; Shi, L.; Zhang, X.-Z.; Chen, S.-Y. and Yu, X.-Q. Metal-Free Oxidative Radical Alkynylation/Ring Expansion Rearrangement of Alkenyl Cyclobutanols with Ethynylbenziodoxolones. *Org. Lett.* **2016**, 18, 4024-4027.

(2) Qin, Y.; Qi, L.; Zhen, X.; Wang, X.; Chai, H.; Ma, X.; Jiang, X.; Cai, X. and Zhu, W. Different Performances of BF_3, BCl_3, and BBr_3 in Hypervalent Iodine-Catalyzed Halogenations. *J. Org. Chem.* **2023**, 88, 4359-4371.

10. NMR Spectra

^{1}H NMR spectrum of **3a** (300 MHz, $CDCl_3$)

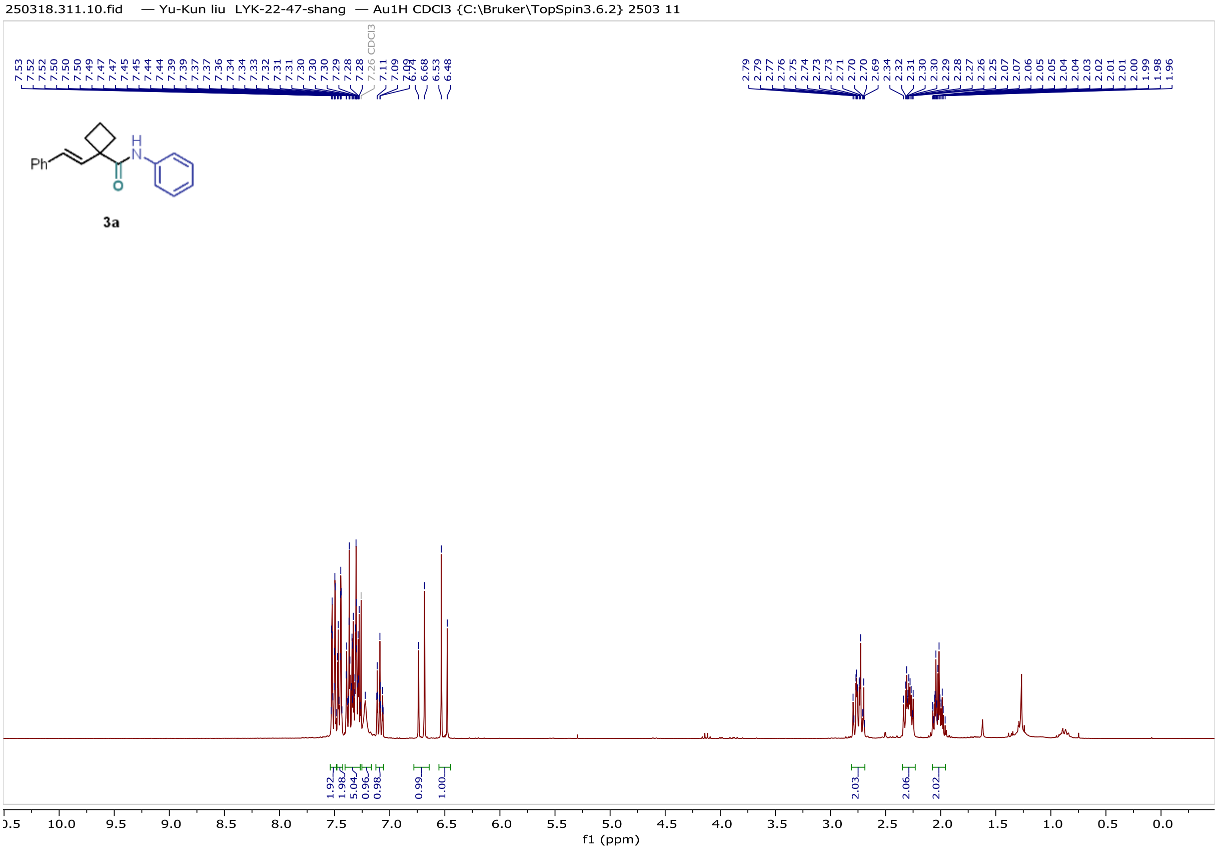

^{13}C NMR spectrum of **3a** (75 MHz, $CDCl_3$)

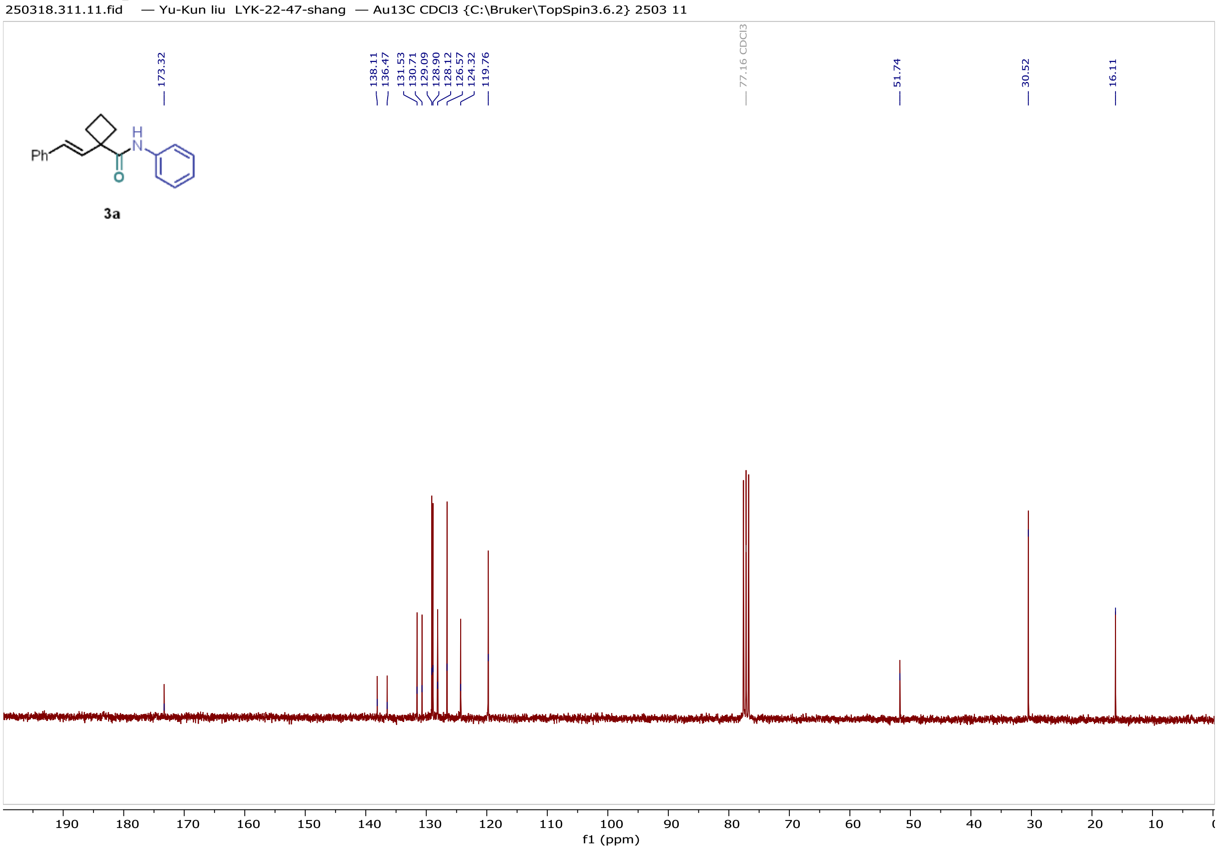

^{1}H NMR spectrum of **3b** (300 MHz, $CDCl_3$)

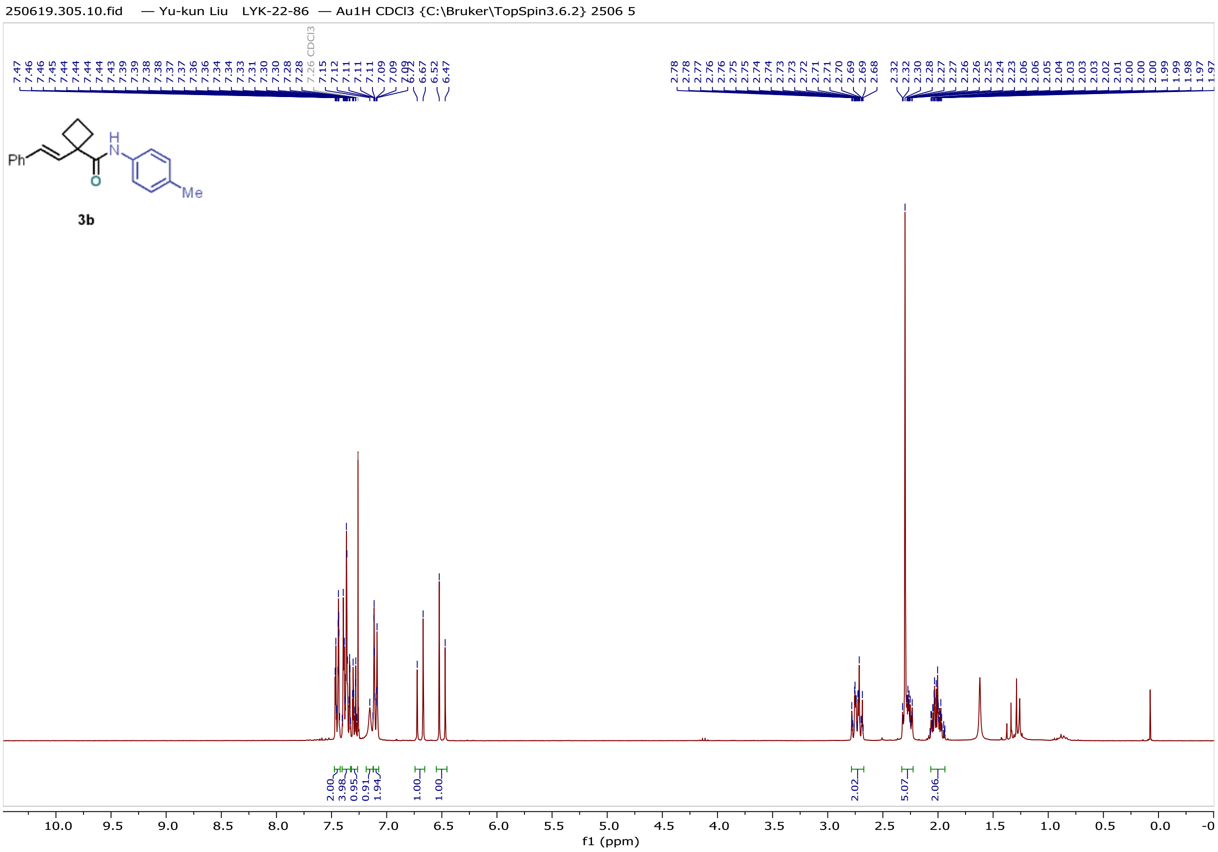

^{13}C NMR spectrum of **3b** (75 MHz, $CDCl_3$)

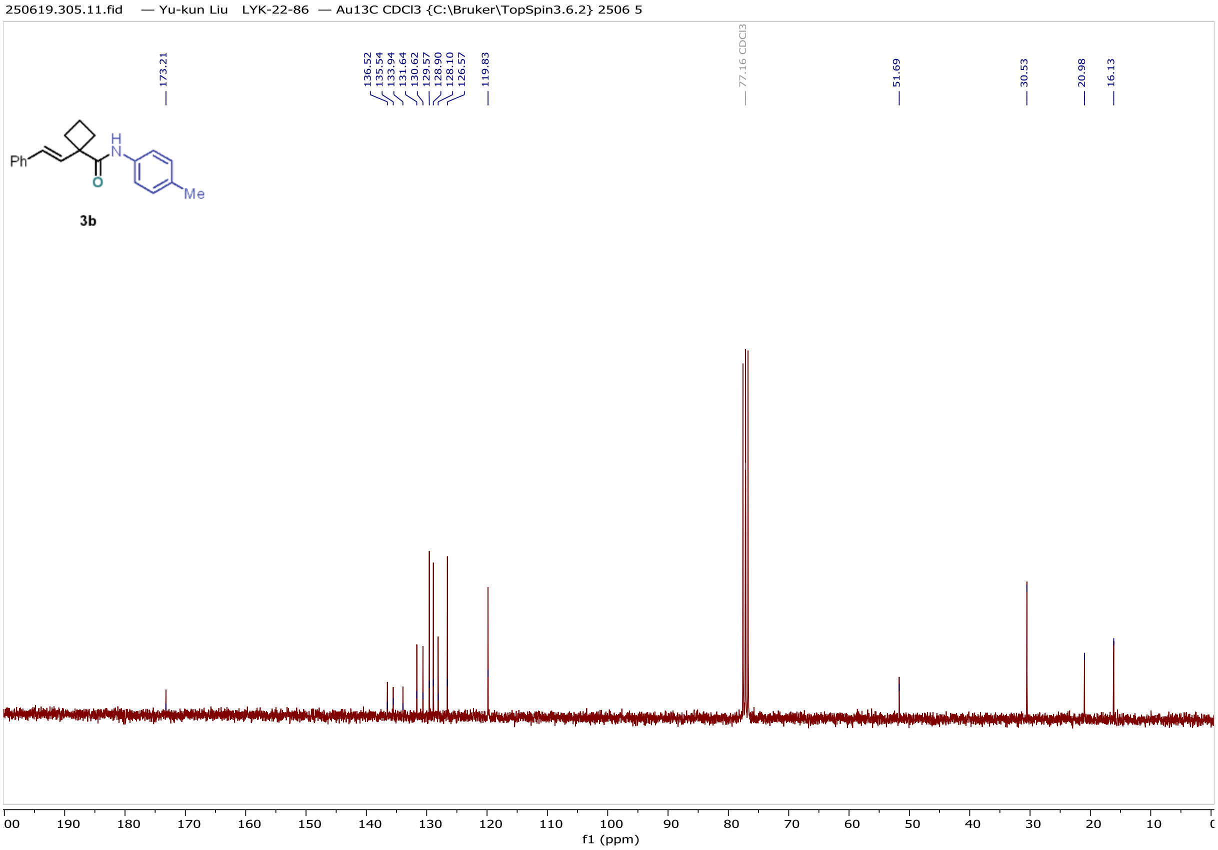

^{1}H NMR spectrum of **3c** (300 MHz, $CDCl_3$)

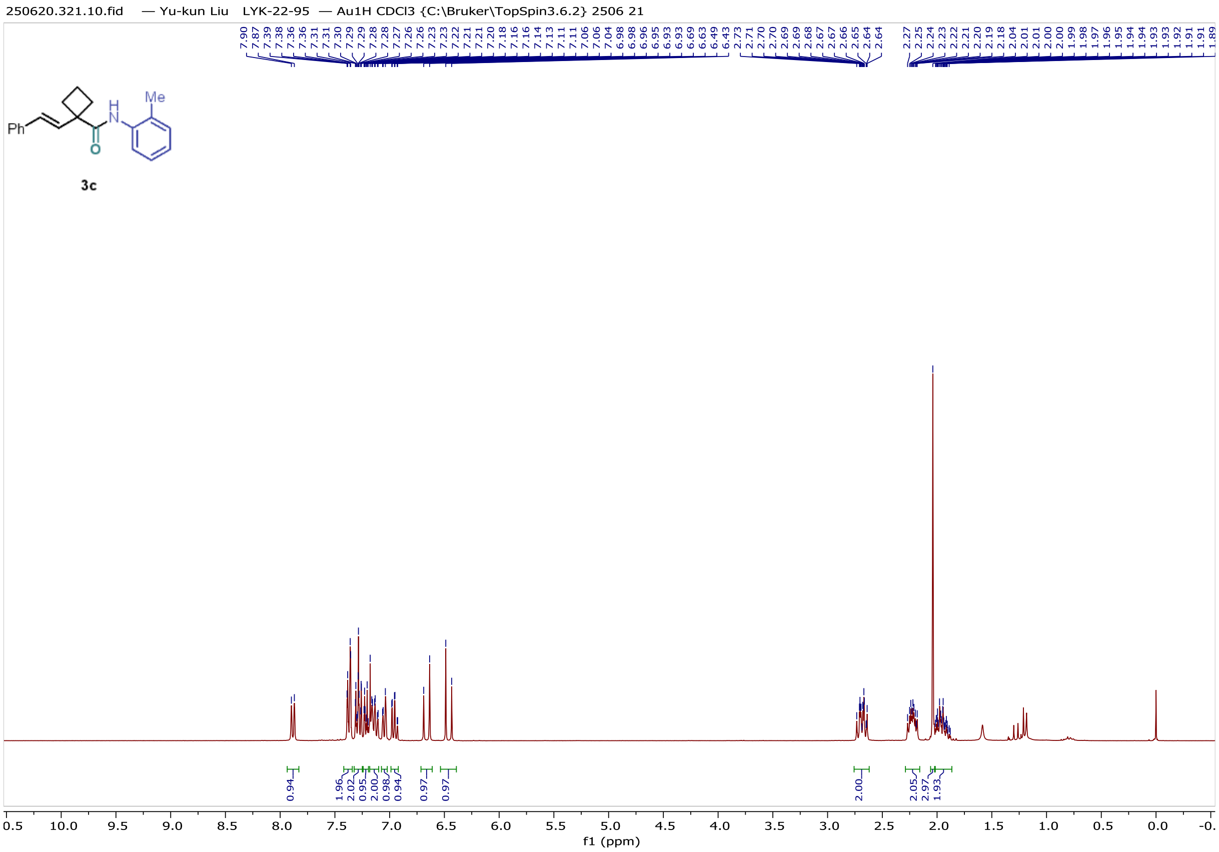

^{13}C NMR spectrum of **3c** (75 MHz, $CDCl_3$)

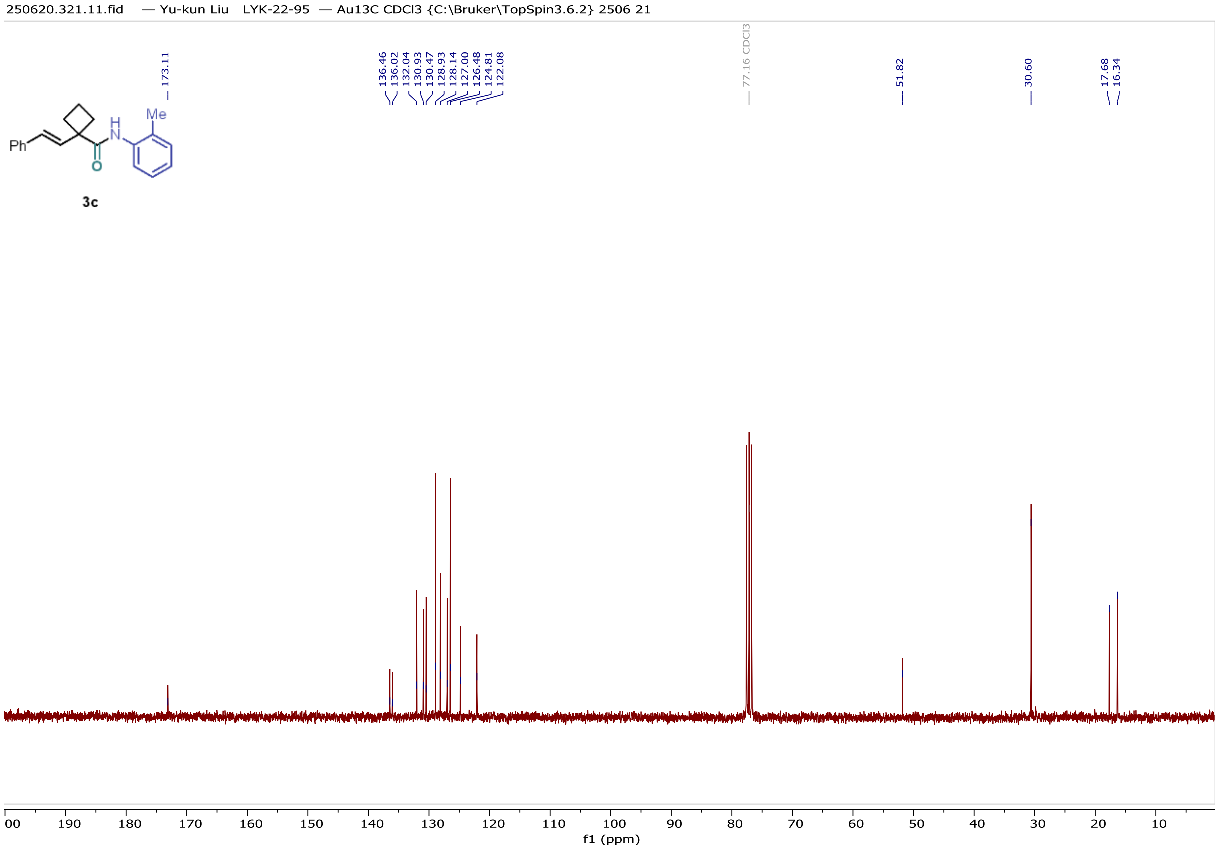

^{1}H NMR spectrum of **3d** (300 MHz, $CDCl_3$)

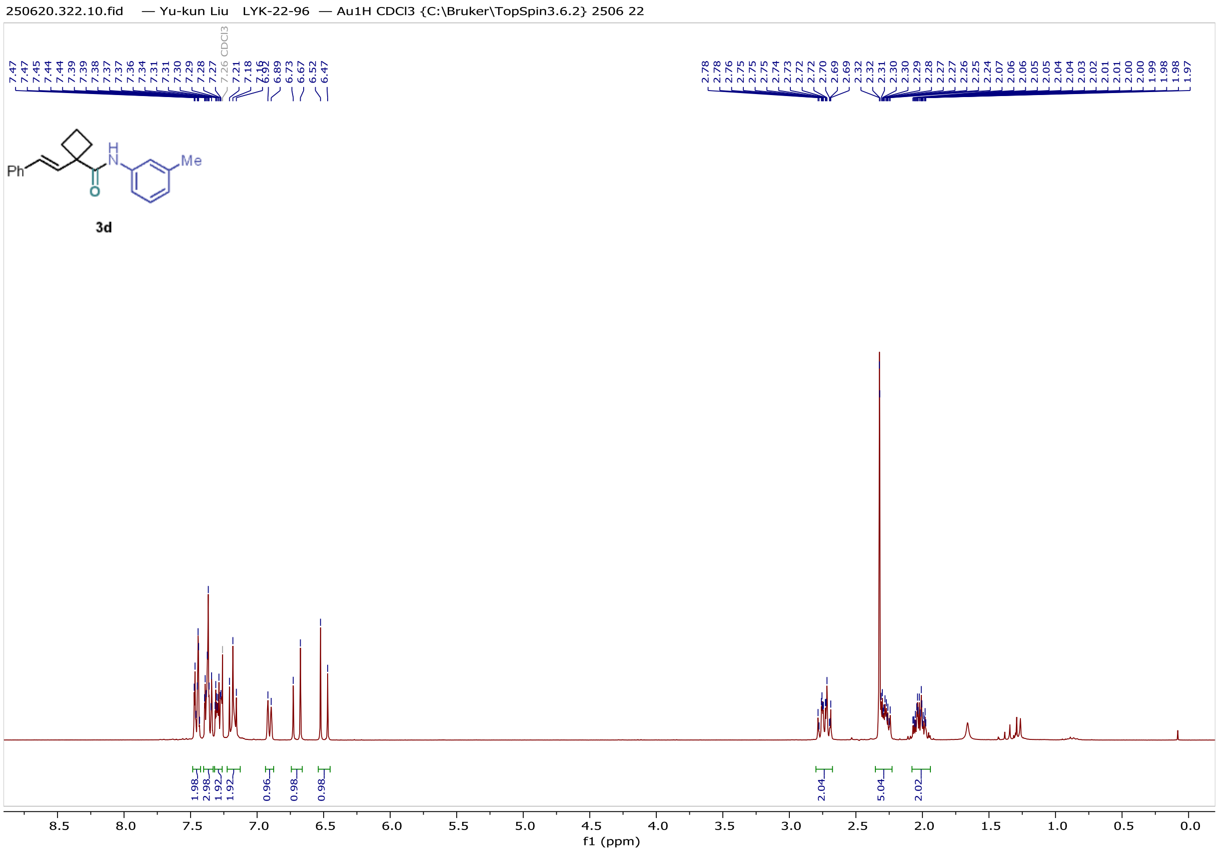

^{13}C NMR spectrum of **3d** (75MHz, $CDCl_3$)

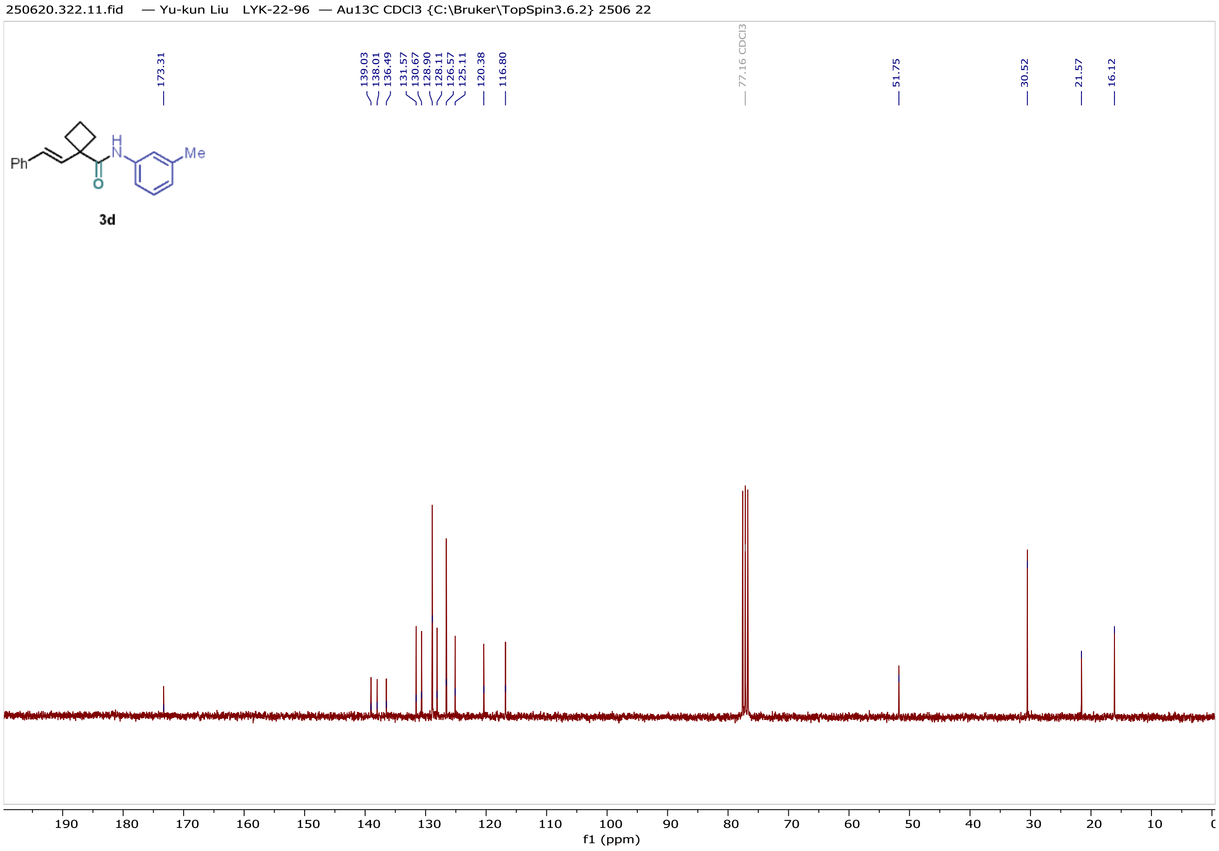

^{1}H NMR spectrum of **3e** (400 MHz, $CDCl_3$)

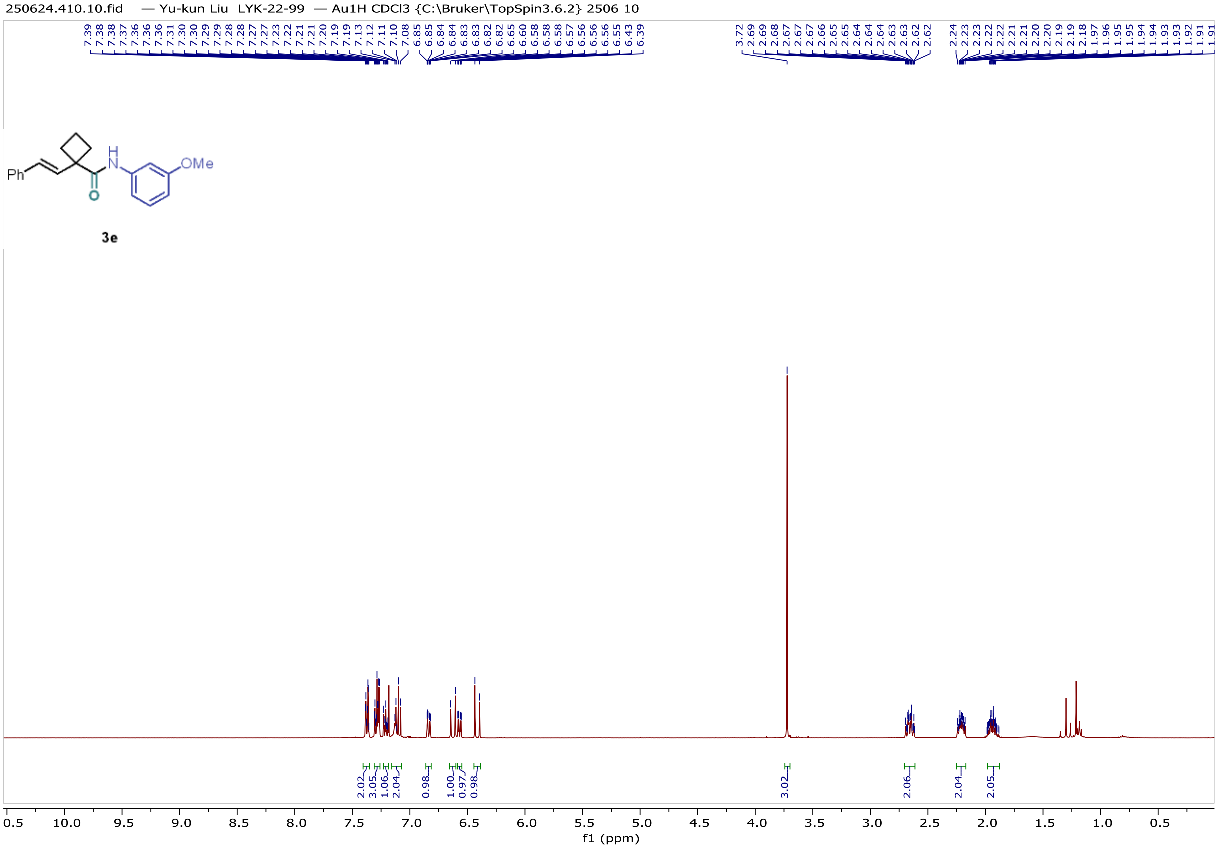

^{13}C NMR spectrum of **3e** (101 MHz, $CDCl_3$)

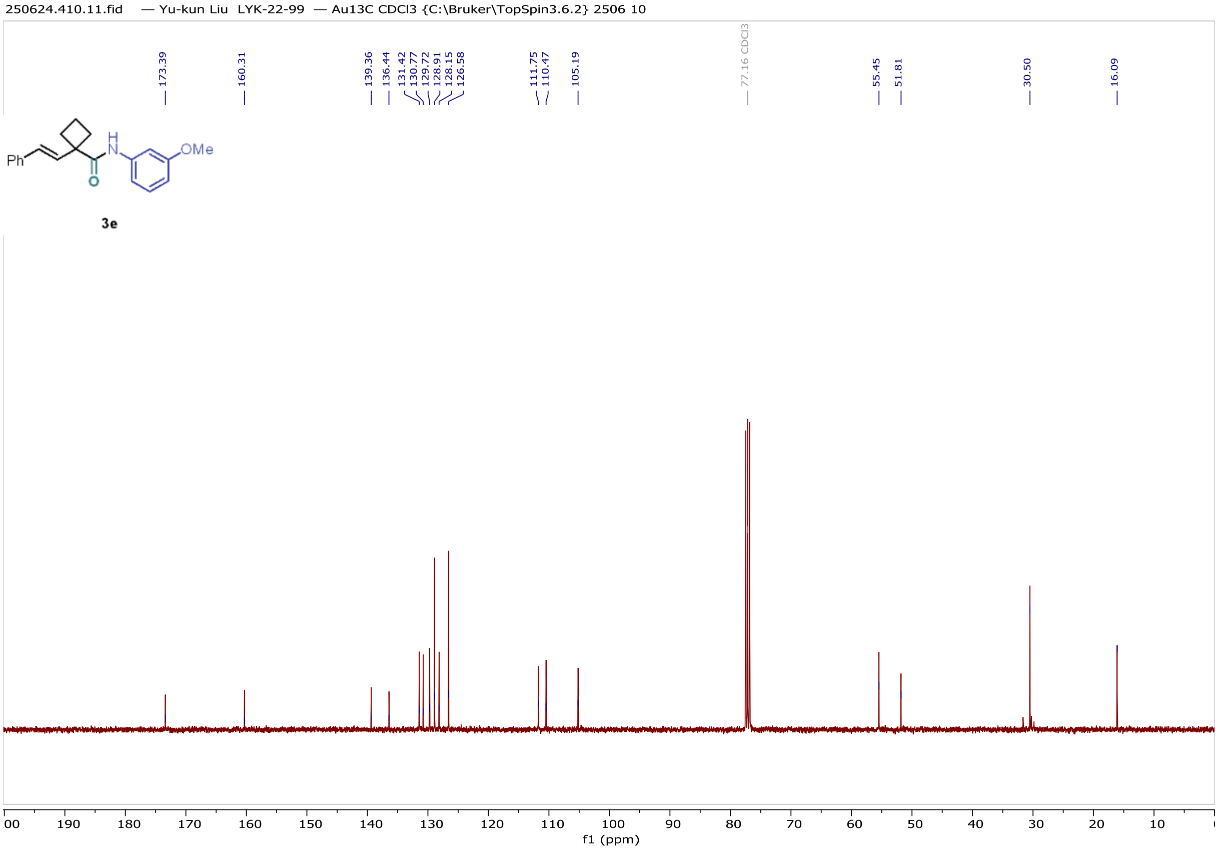

^{1}H NMR spectrum of **3f** (400 MHz, $CDCl_3$)

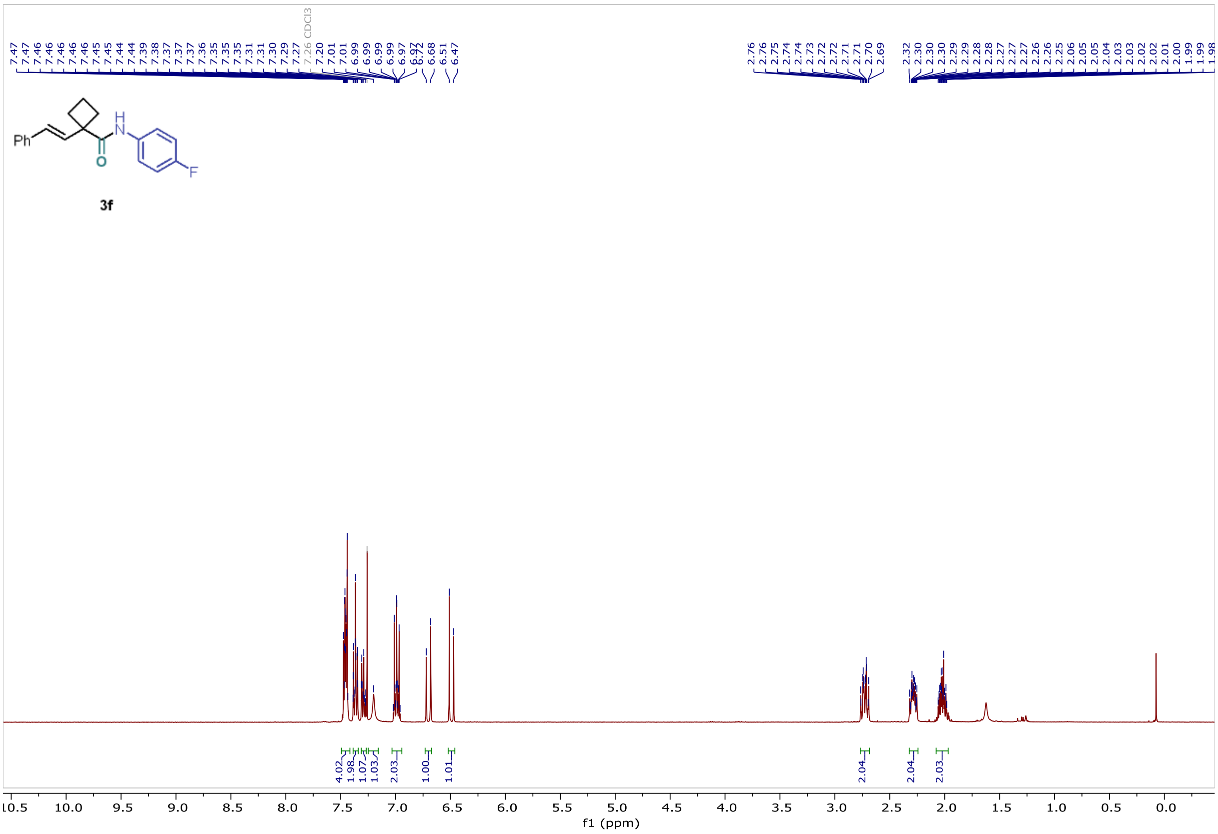

^{13}C NMR spectrum of **3f** (101 MHz, $CDCl_3$)

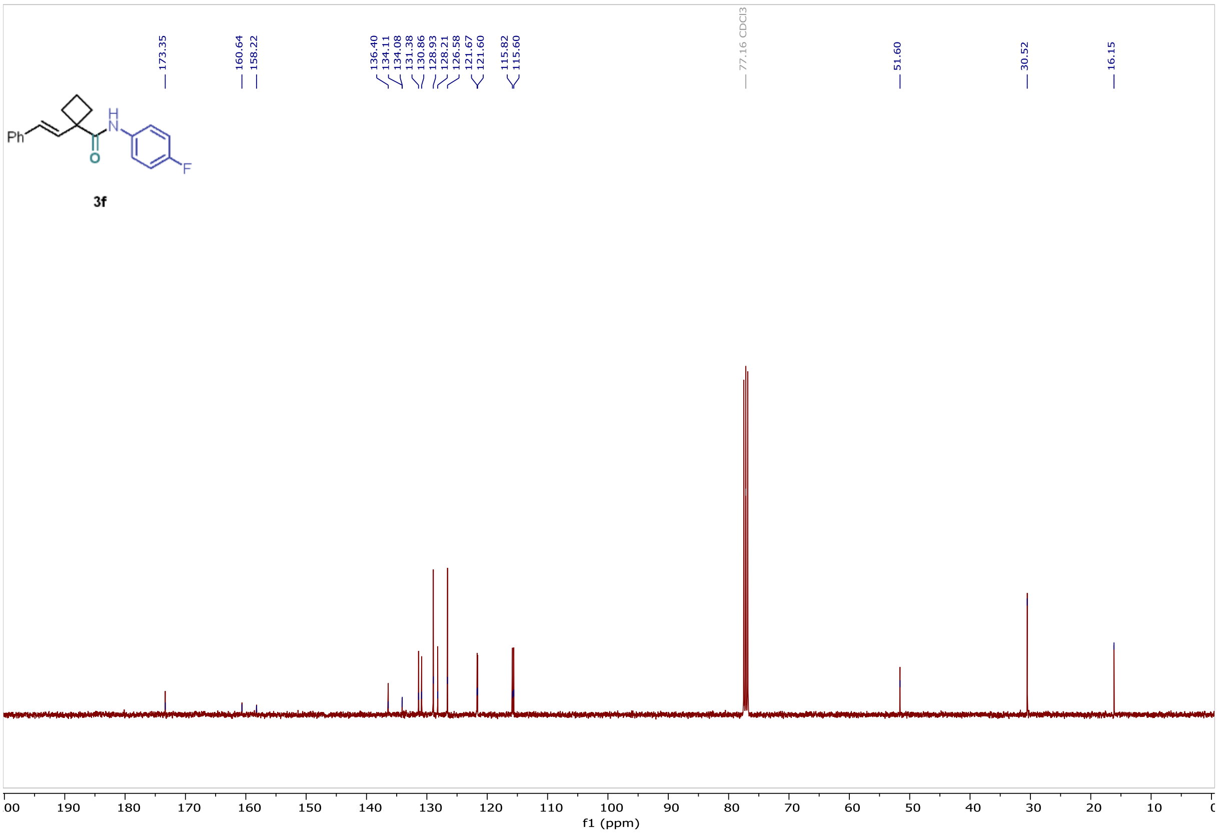

^{19}F NMR spectrum of **3f** (376 MHz, $CDCl_3$)

250624.415.12.fid — Yu-kun Liu LYK-22-103 — Au19F CDCl3 {C:\Bruker\TopSpin3.6.2} 2506 15

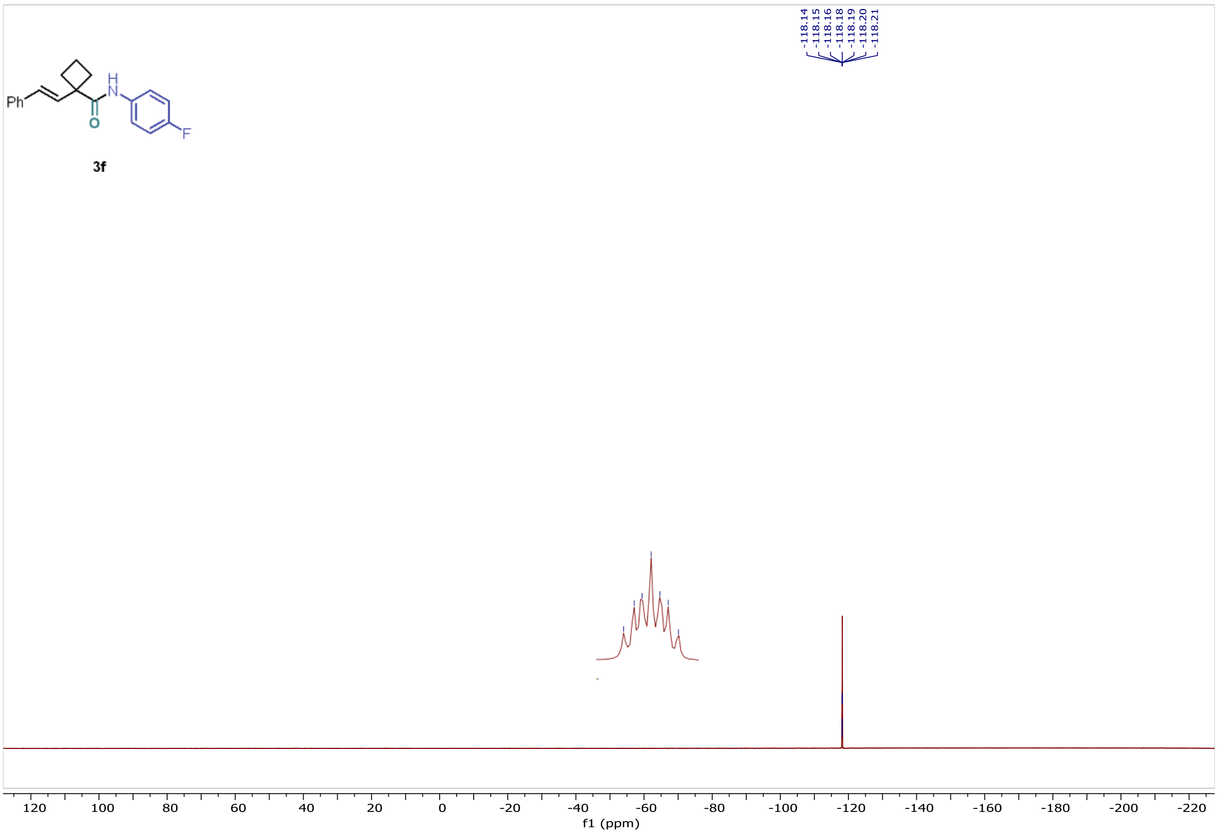

^{1}H NMR spectrum of **3g** (300 MHz, $CDCl_3$)

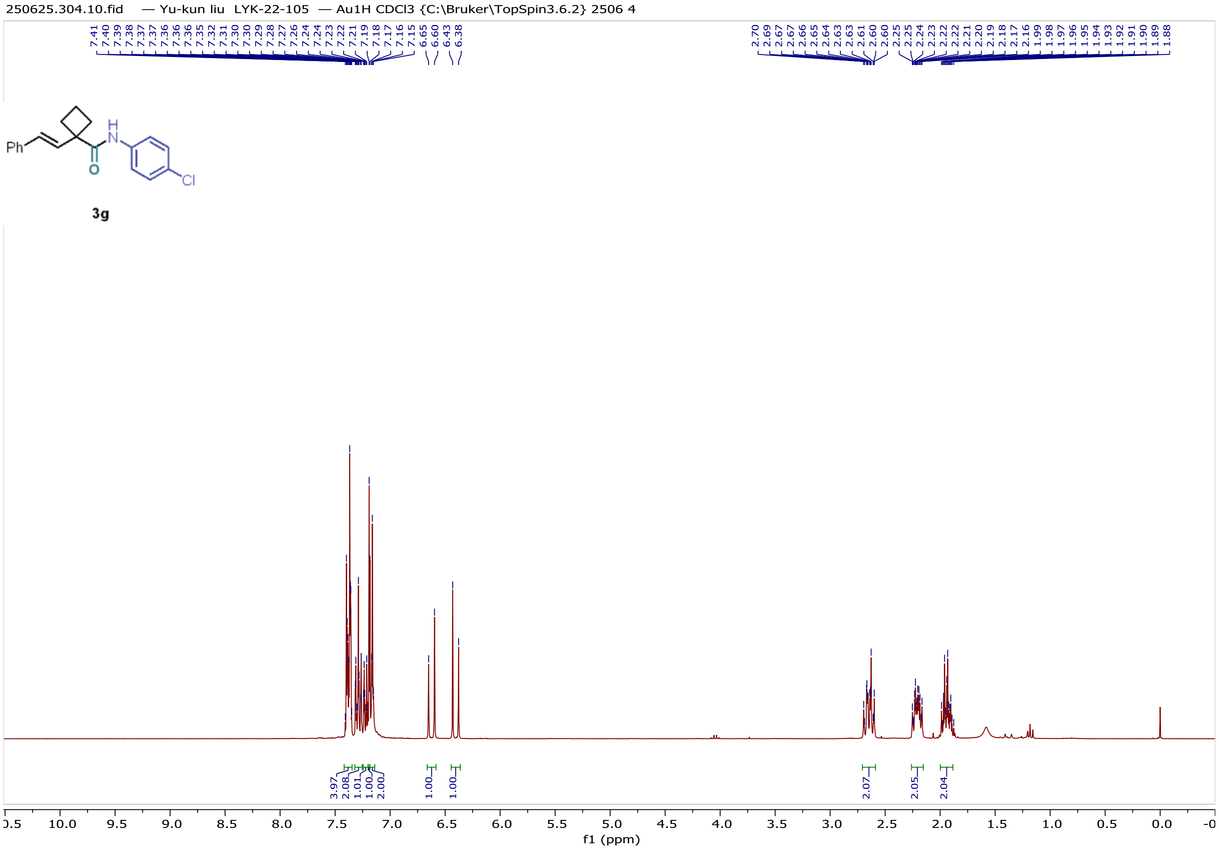

^{13}C NMR spectrum of **3g** (75 MHz, $CDCl_3$)

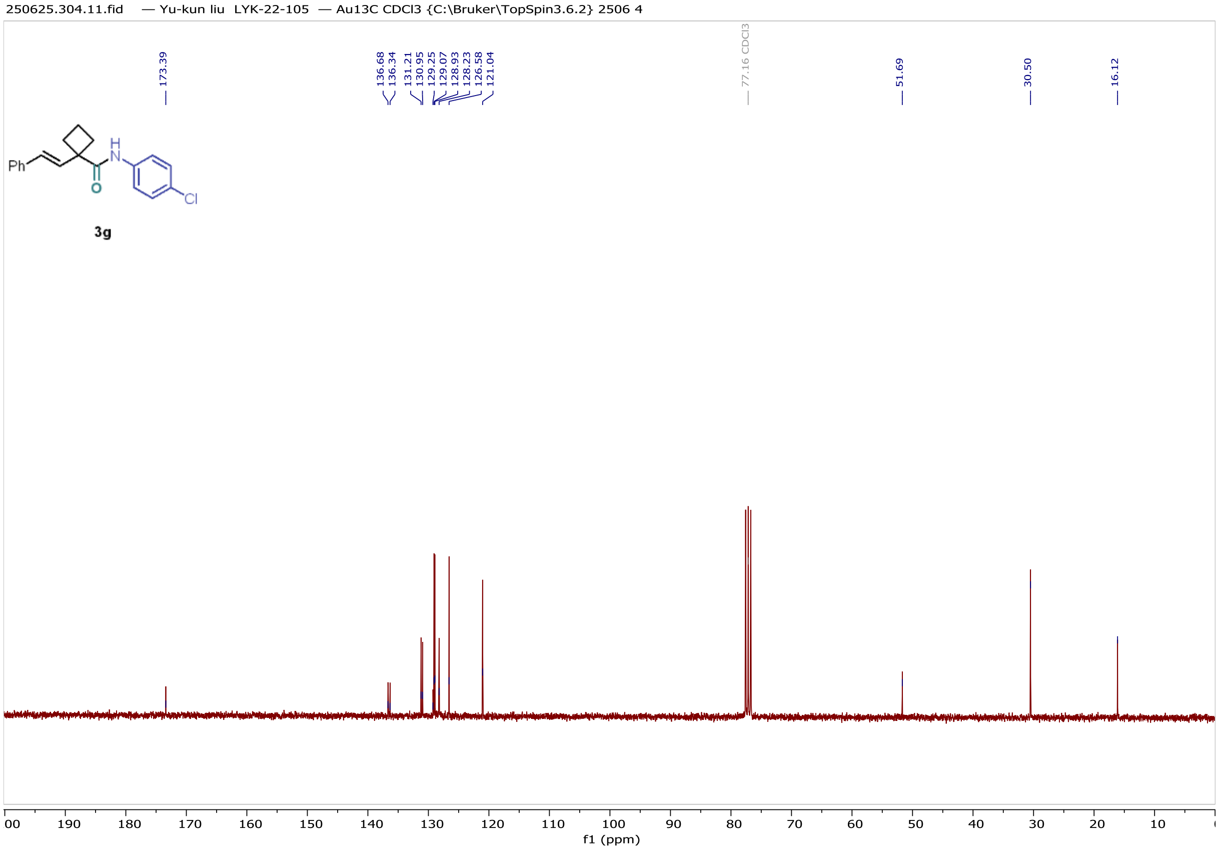

^{1}H NMR spectrum of **3h** (300 MHz, $CDCl_3$)

250619.307.10.fid — Yu-kun Liu LYK-22-88 — Au1H CDCl3 {C:\Bruker\TopSpin3.6.2} 2506 7

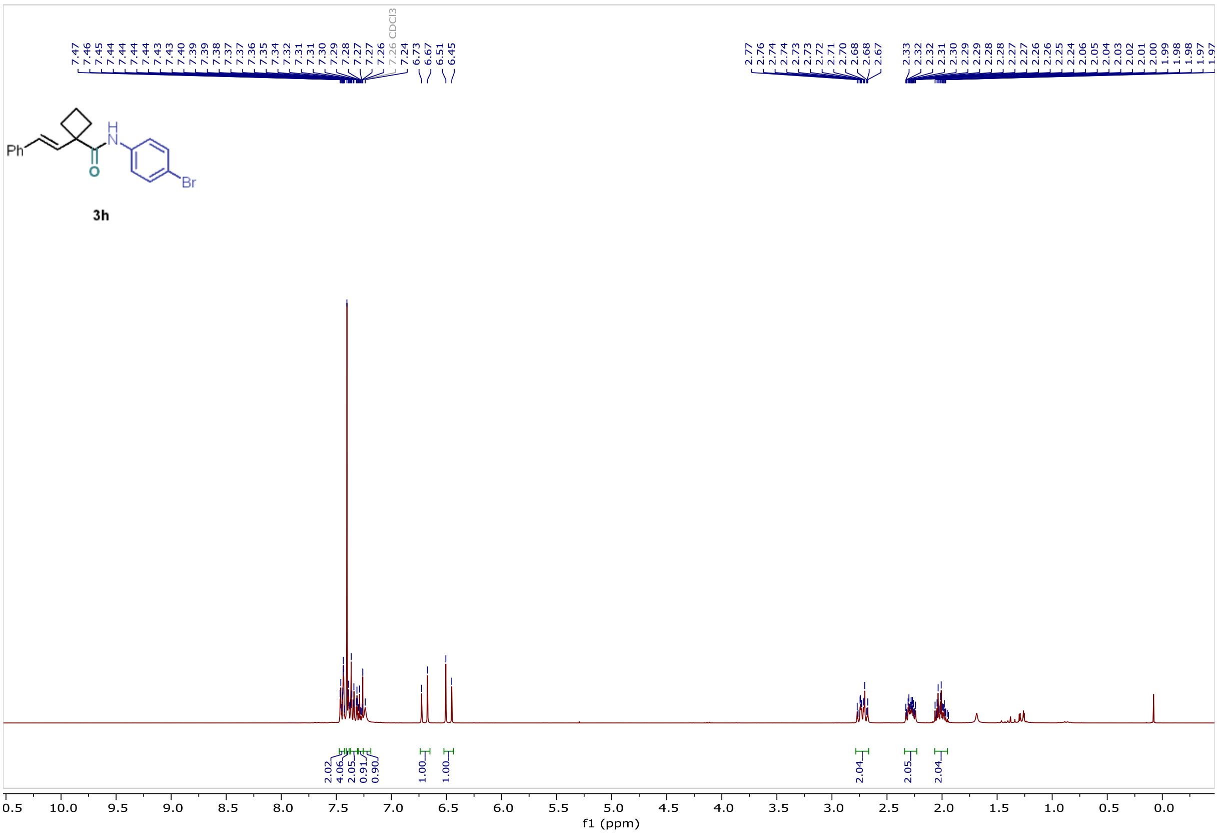

^{13}C NMR spectrum of **3h** (75 MHz, $CDCl_3$)

250619.307.11.fid — Yu-kun Liu LYK-22-88 — Au13C CDCl3 {C:\Bruker\TopSpin3.6.2} 2506 7

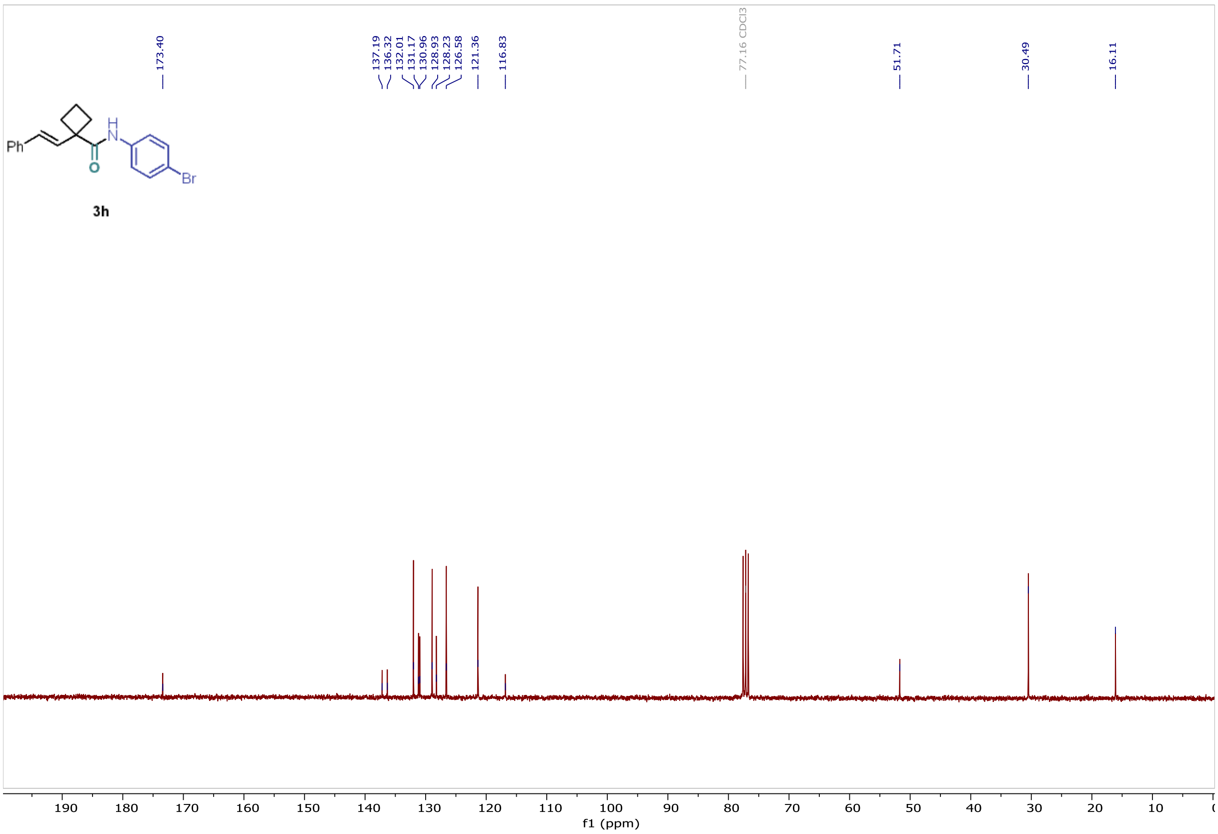

^{1}H NMR spectrum of **3i** (300 MHz, $CDCl_3$)

250619.303.10.fid — Yu-kun Liu LYK-22-84 — Au1H CDCl3 {C:\Bruker\TopSpin3.6.2} 2506 3

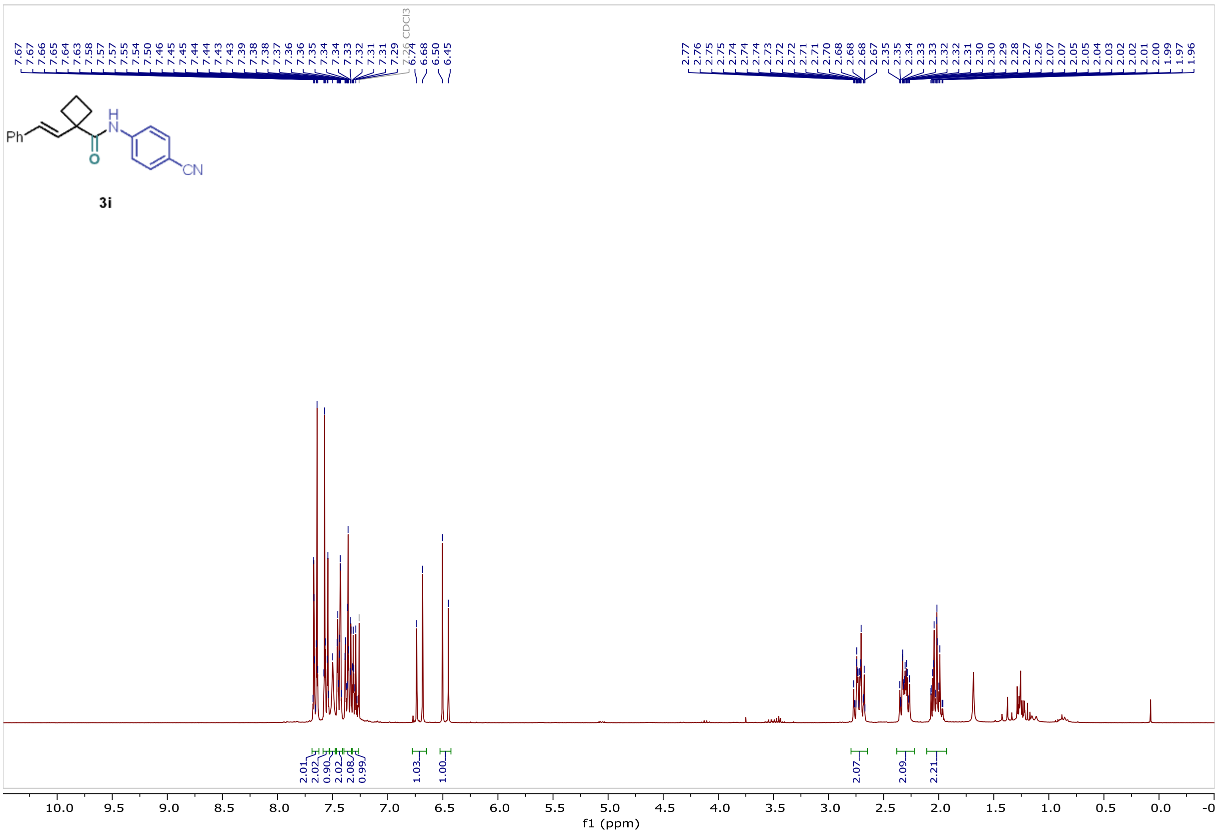

^{13}C NMR spectrum of **3i** (75 MHz, $CDCl_3$)

250619.303.11.fid — Yu-kun Liu LYK-22-84 — Au13C CDCl3 {C:\Bruker\TopSpin3.6.2} 2506 3

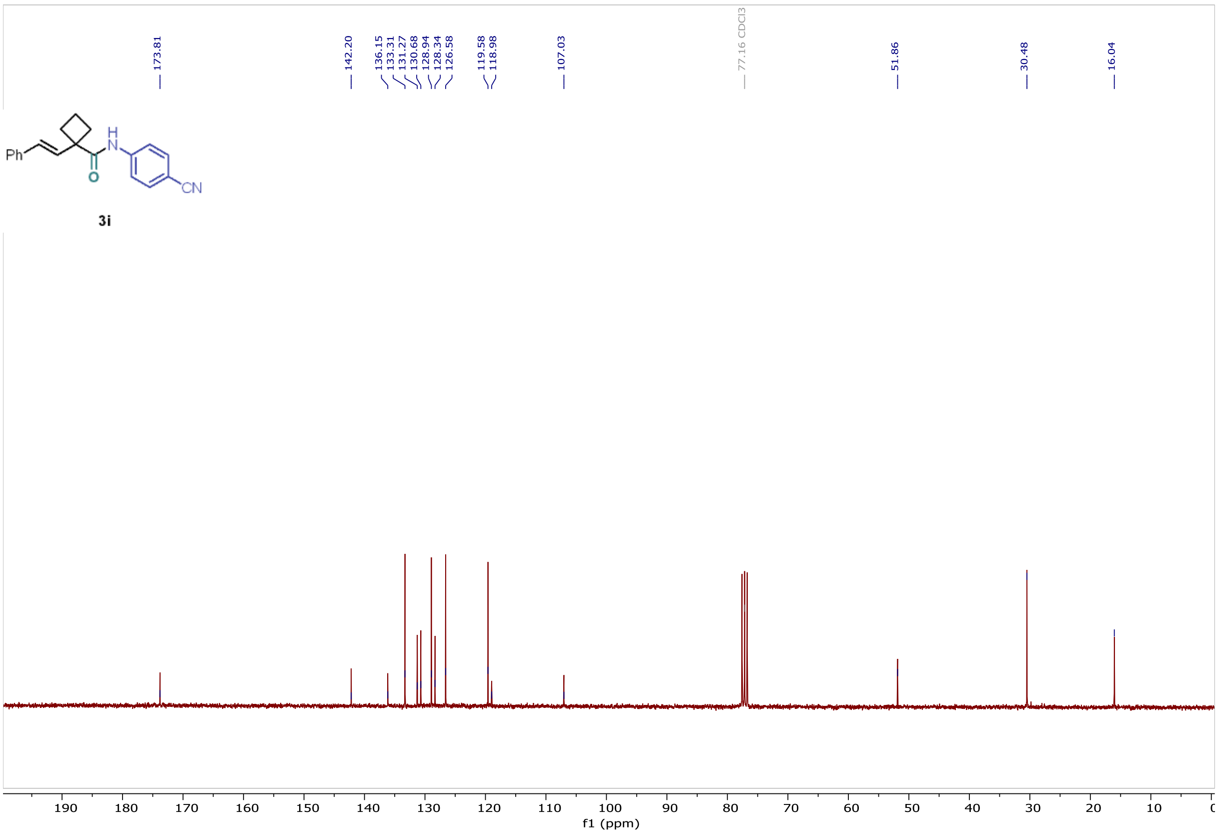

^{1}H NMR spectrum of **3j** (300 MHz, $CDCl_3$)

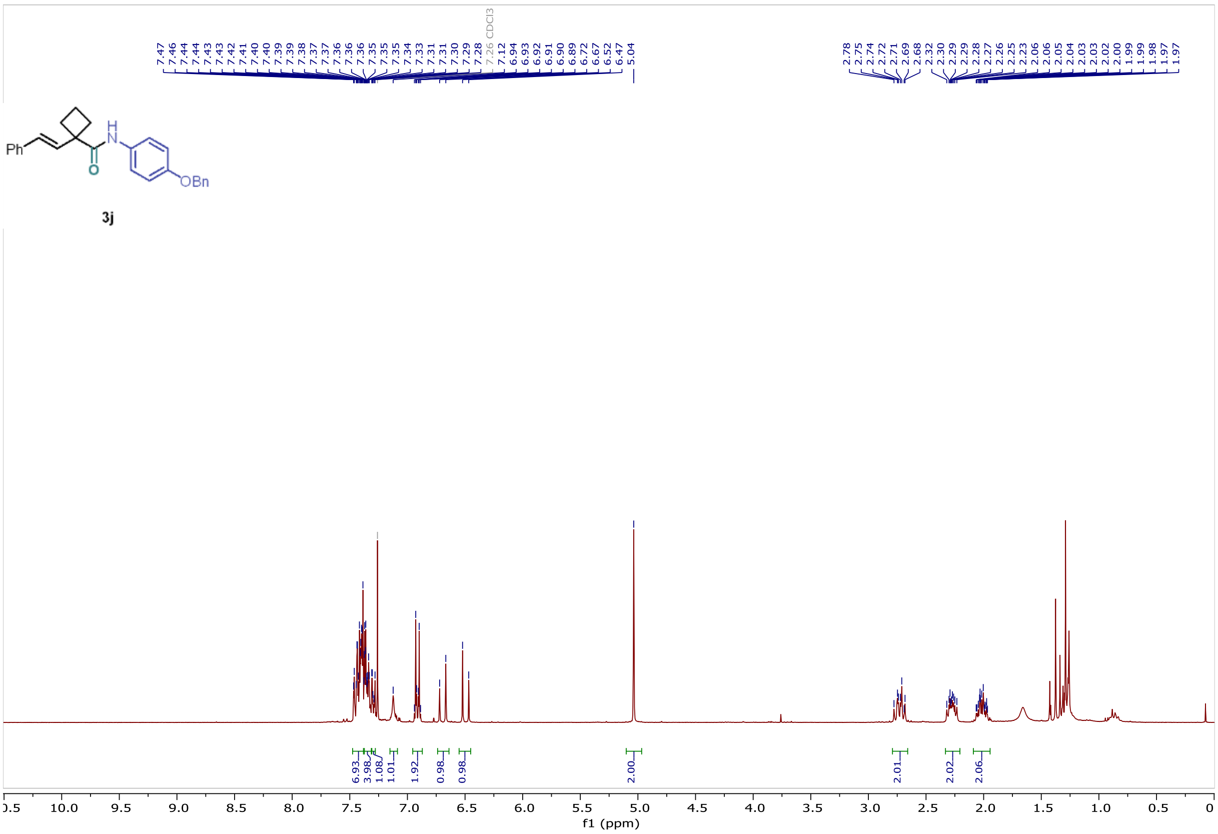

^{13}C NMR spectrum of **3j** (75 MHz, $CDCl_3$)

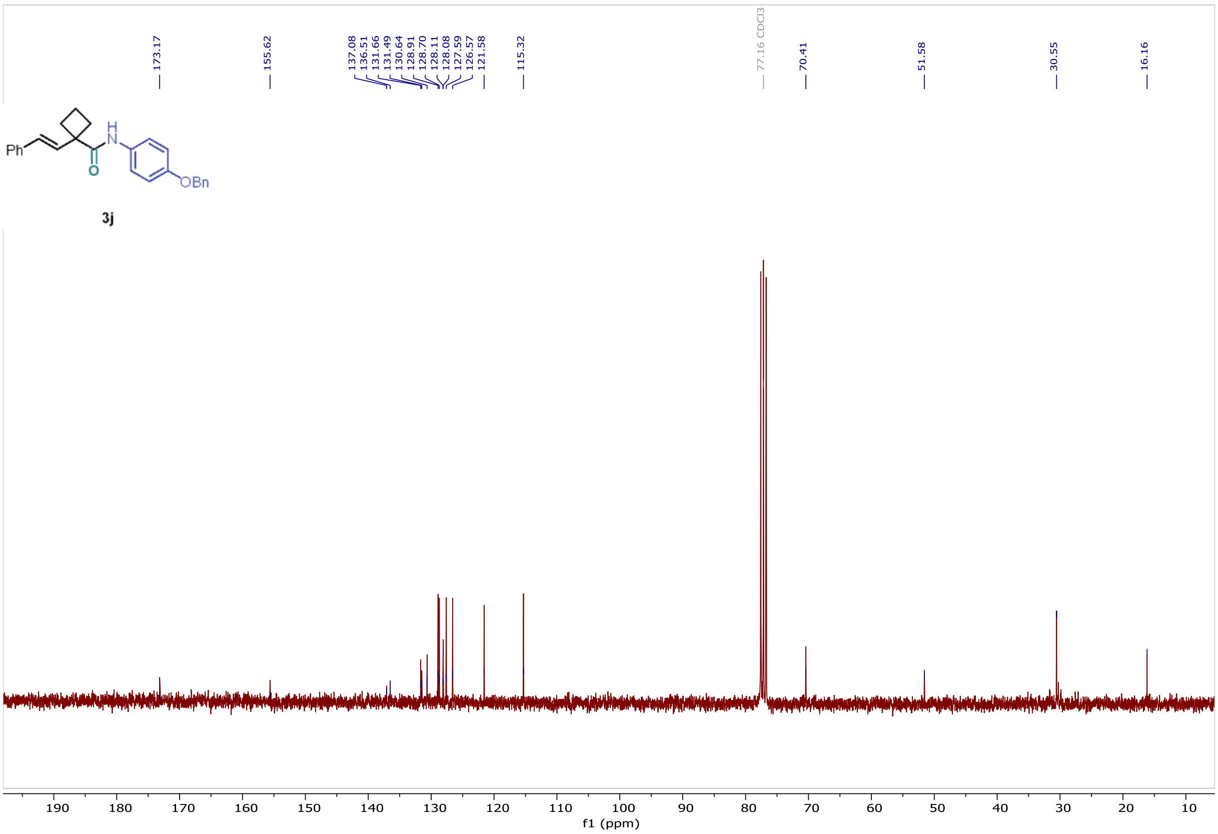

^{1}H NMR spectrum of **3k** (300 MHz, $CDCl_3$)

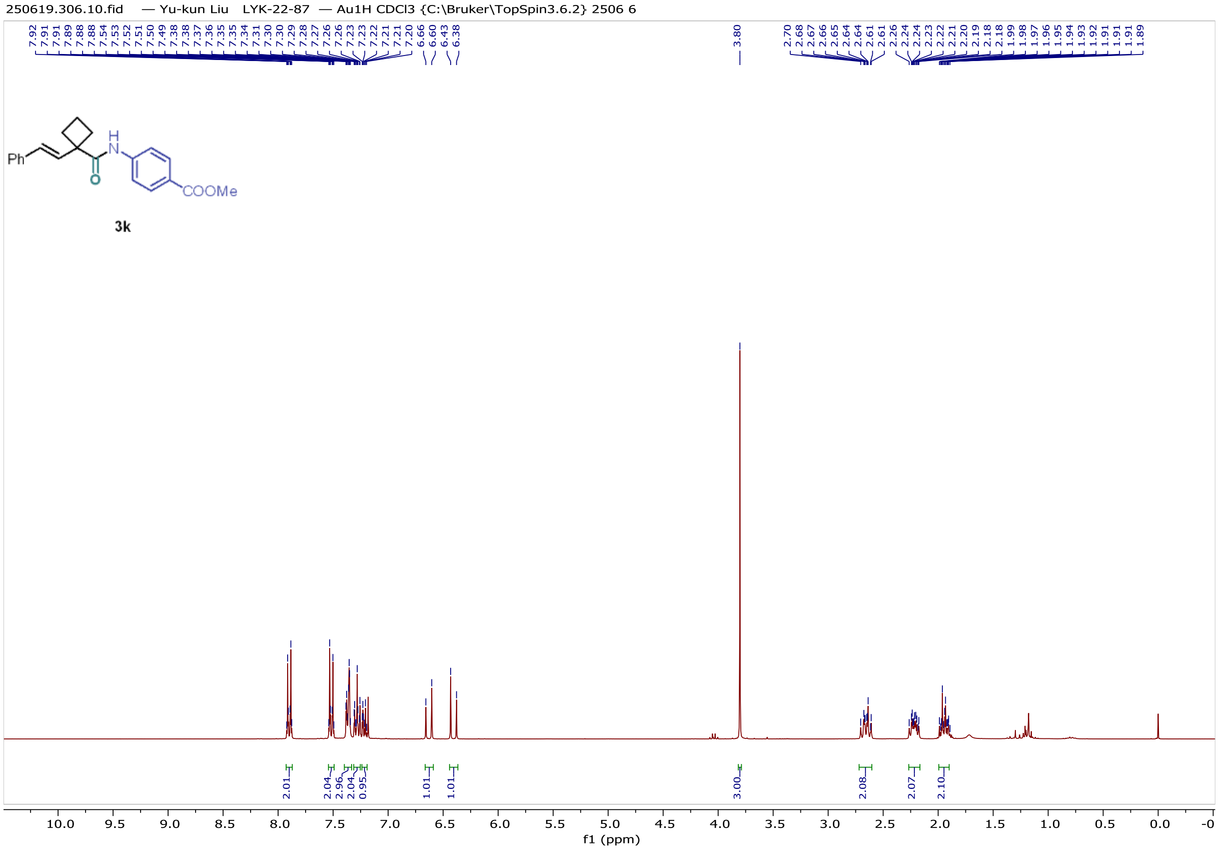

^{13}C NMR spectrum of **3k** (75MHz, $CDCl_3$)

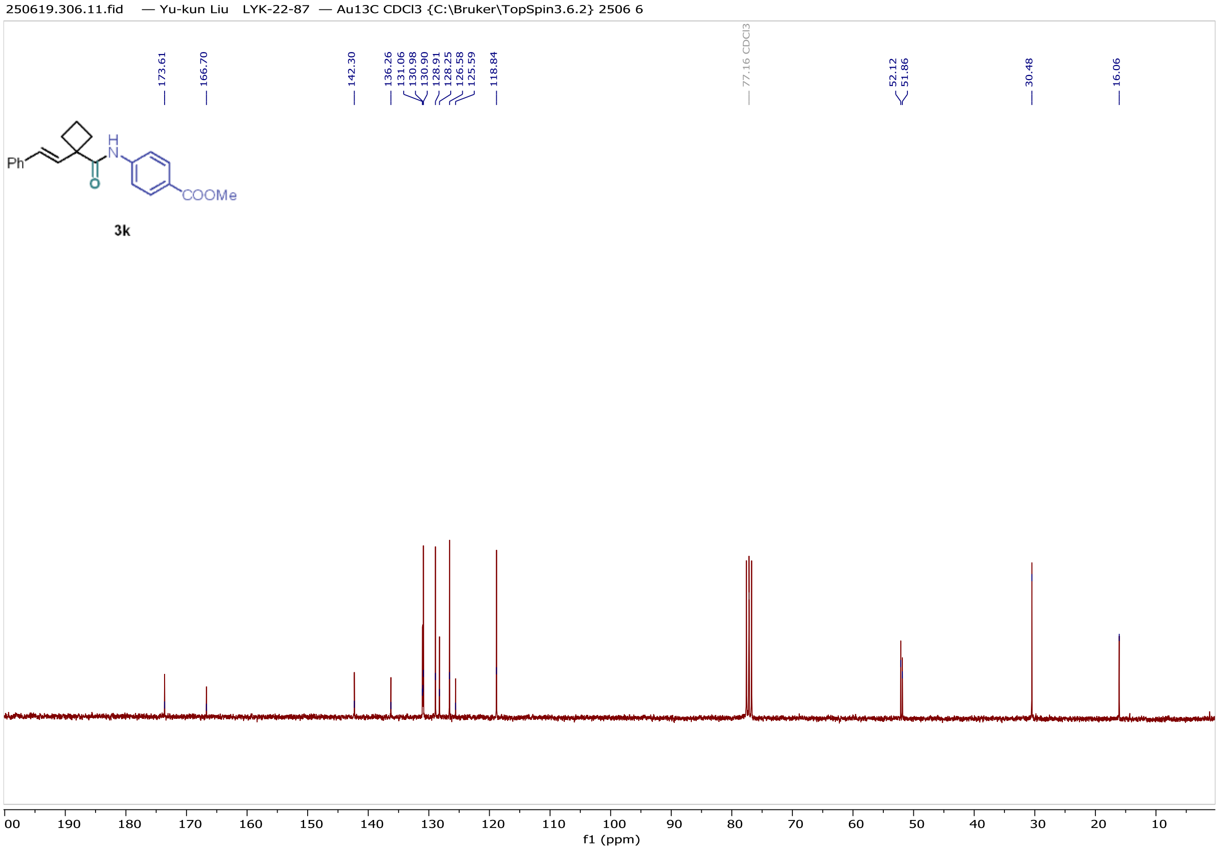

^{1}H NMR spectrum of **3l** (300 MHz, $CDCl_3$)

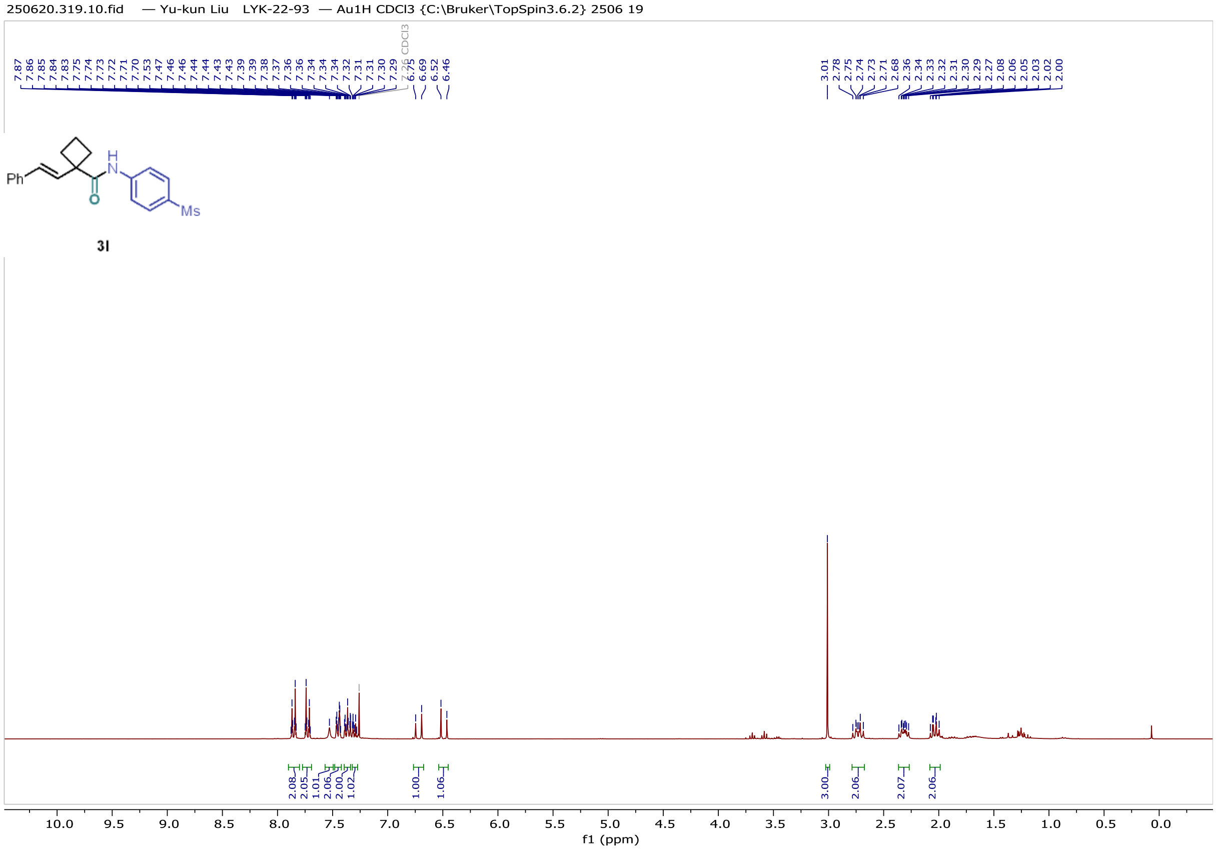

^{13}C NMR spectrum of **3l** (75 MHz, $CDCl_3$)

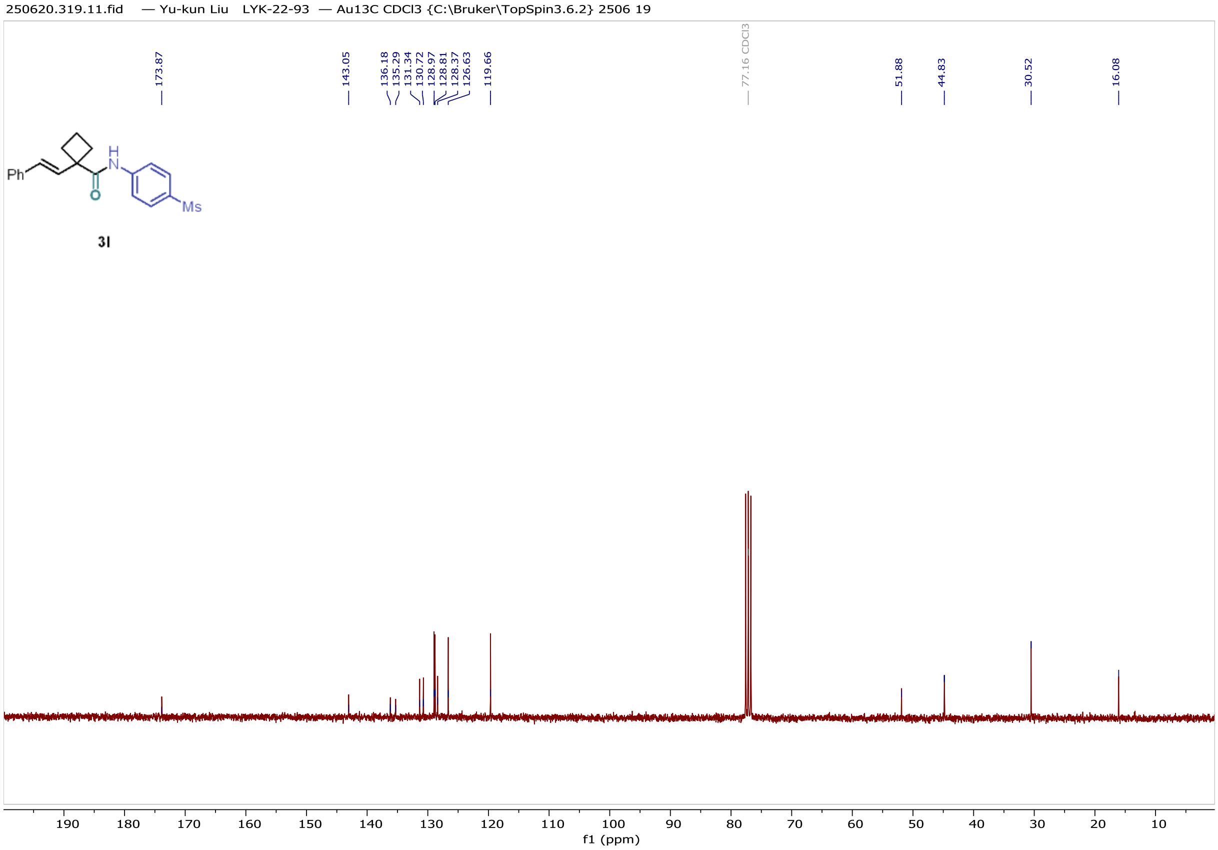

^{1}H NMR spectrum of **3m** (300 MHz, $CDCl_3$)

250619.308.10.fid — Yu-kun Liu LYK-22-89 — Au1H CDCl3 {C:\Bruker\TopSpin3.6.2} 2506 8

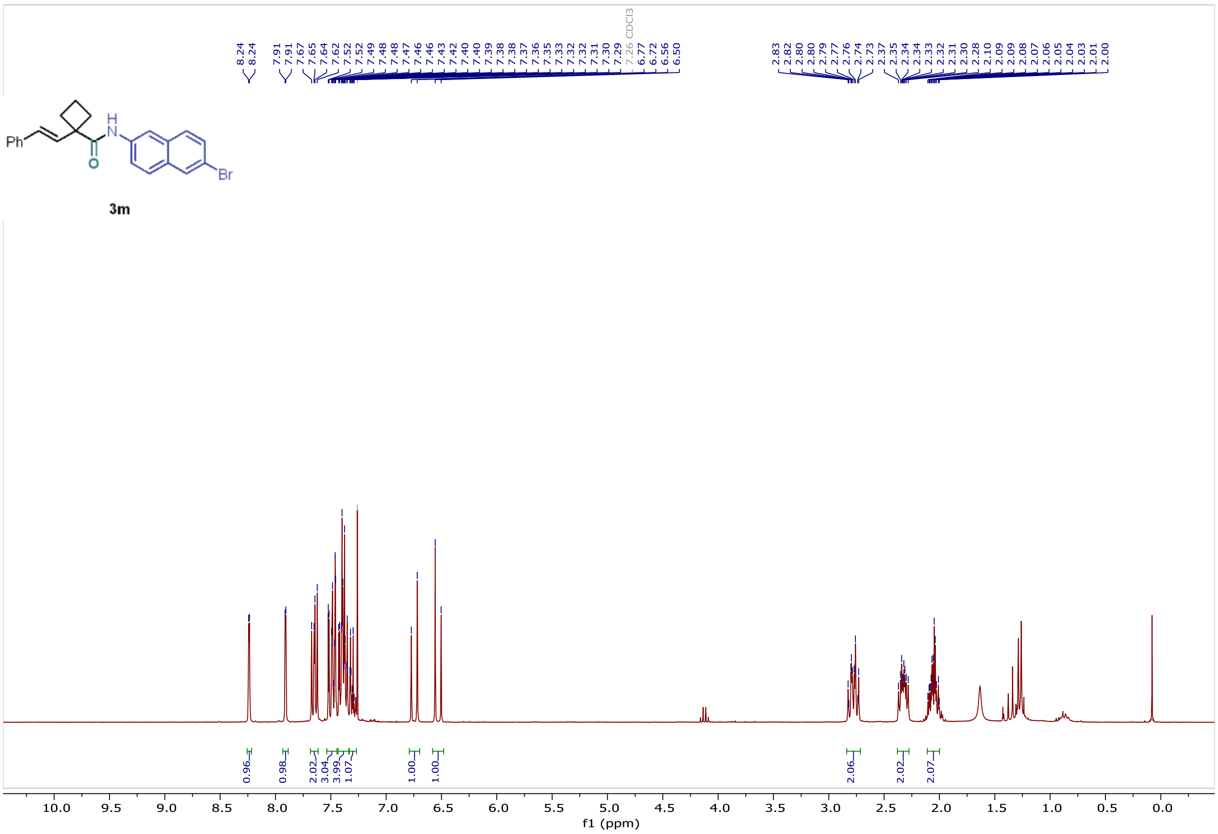

^{13}C NMR spectrum of **3m** (75 MHz, $CDCl_3$)

250619.308.11.fid — Yu-kun Liu LYK-22-89 — Au13C CDCl3 {C:\Bruker\TopSpin3.6.2} 2506 8

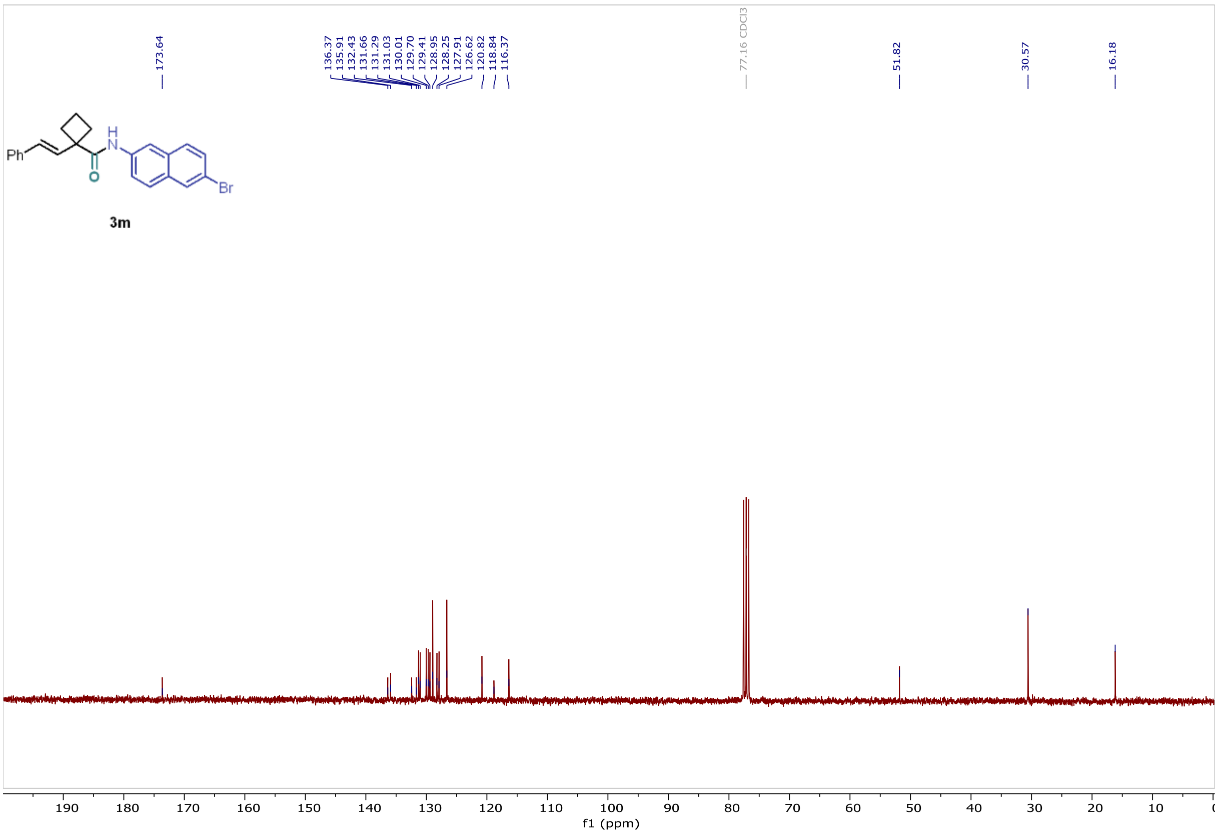

^{1}H NMR spectrum of **3n** (400 MHz, $CDCl_3$)

250624.413.10.fid — Yu-kun Liu LYK-22-102 — Au1H CDCl3 {C:\Bruker\TopSpin3.6.2} 2506 13

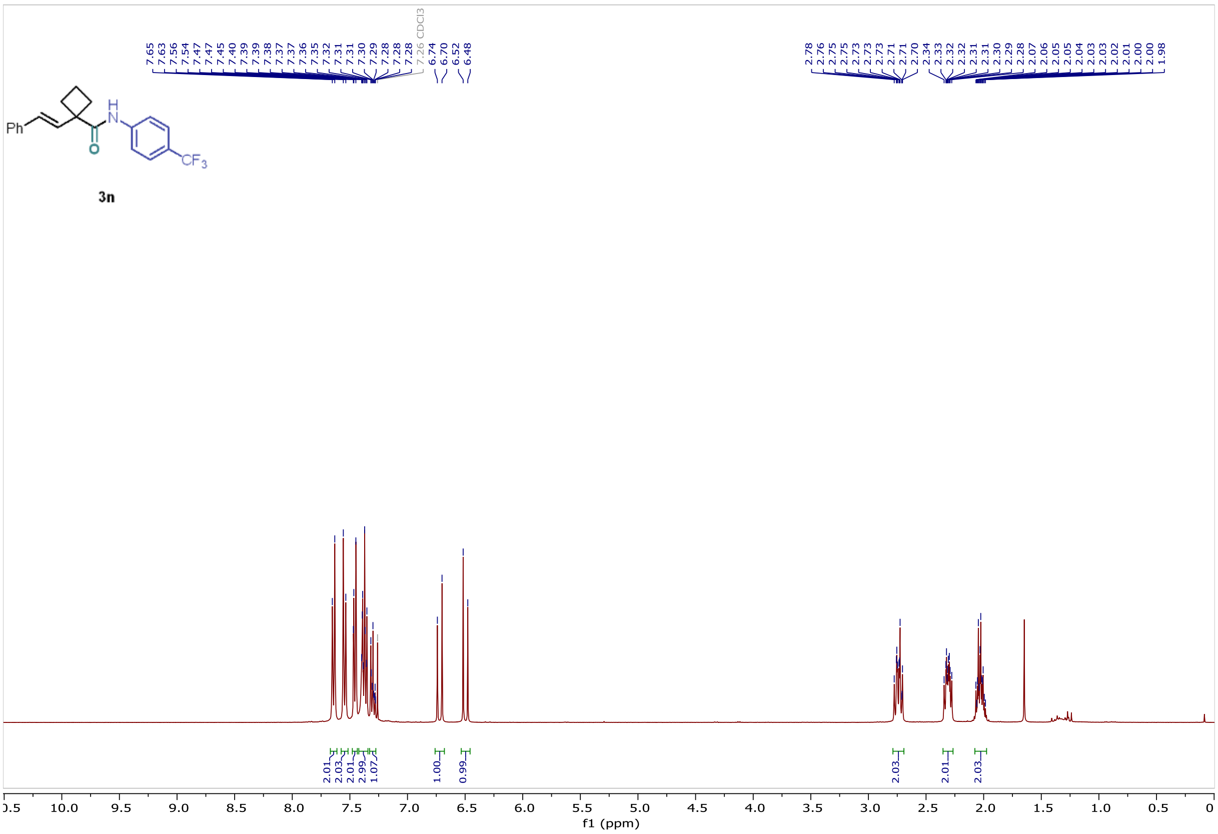

^{13}C NMR spectrum of **3n** (101 MHz, $CDCl_3$)

250624.413.11.fid — Yu-kun Liu LYK-22-102 — Au13C CDCl3 {C:\Bruker\TopSpin3.6.2} 2506 13

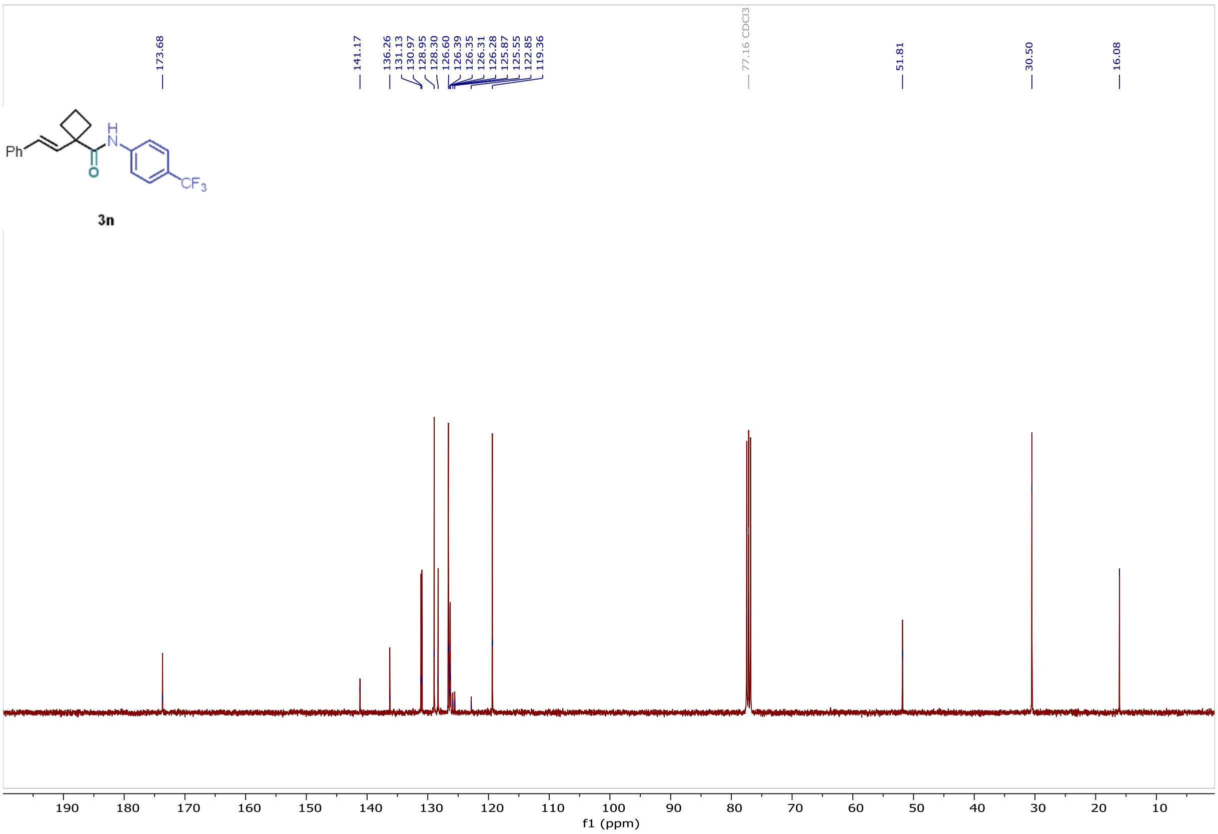

^{19}F NMR spectrum of **3n** (376 MHz, $CDCl_3$)

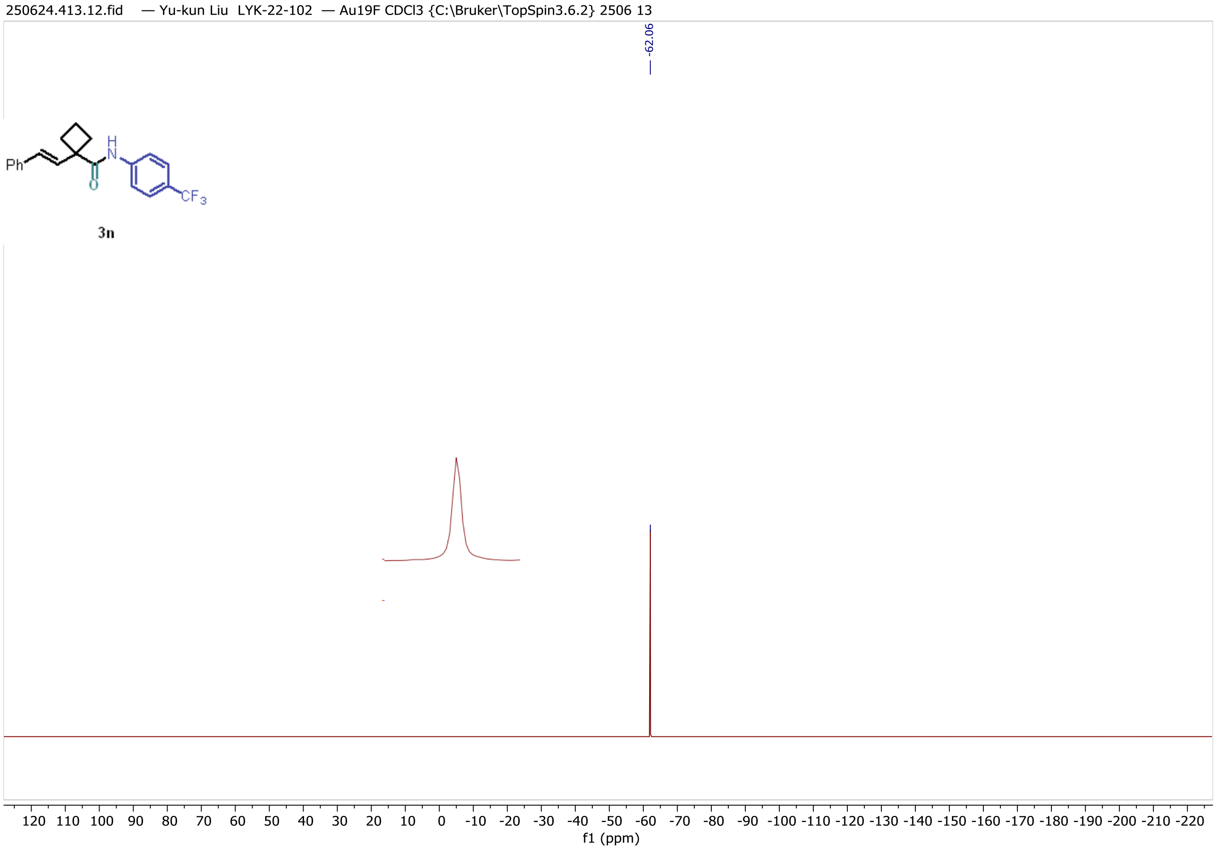

^{1}H NMR spectrum of **3o** (300 MHz, $CDCl_3$)

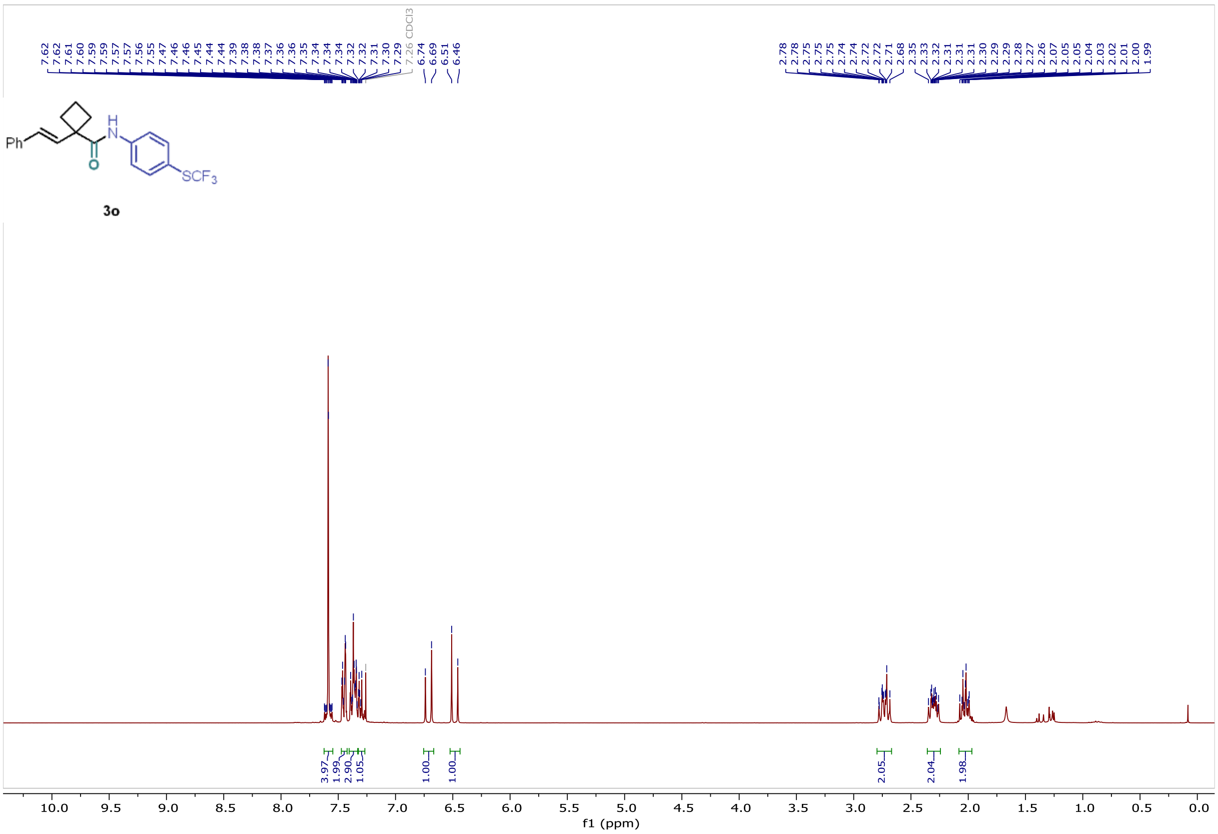

^{13}C NMR spectrum of **3o** (75 MHz, $CDCl_3$)

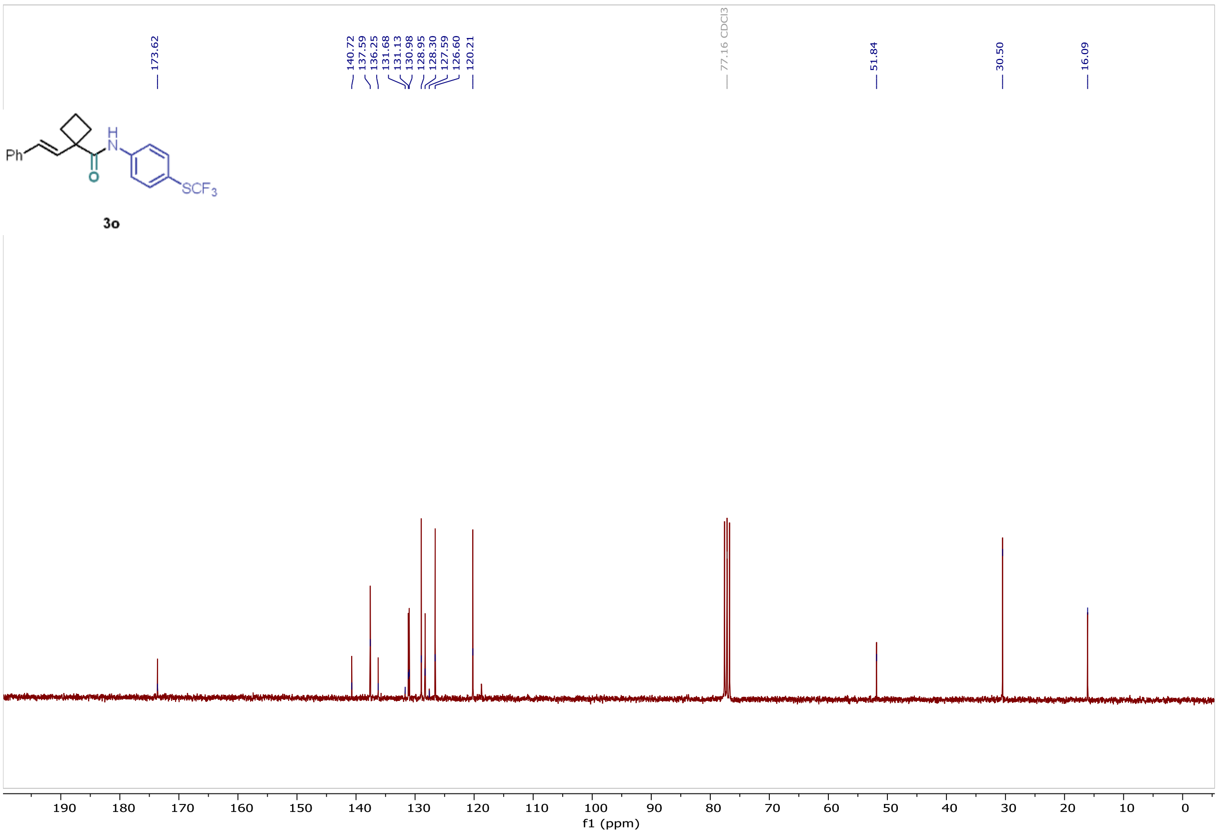

^{19}F NMR spectrum of **3o** (282 MHz, $CDCl_3$)

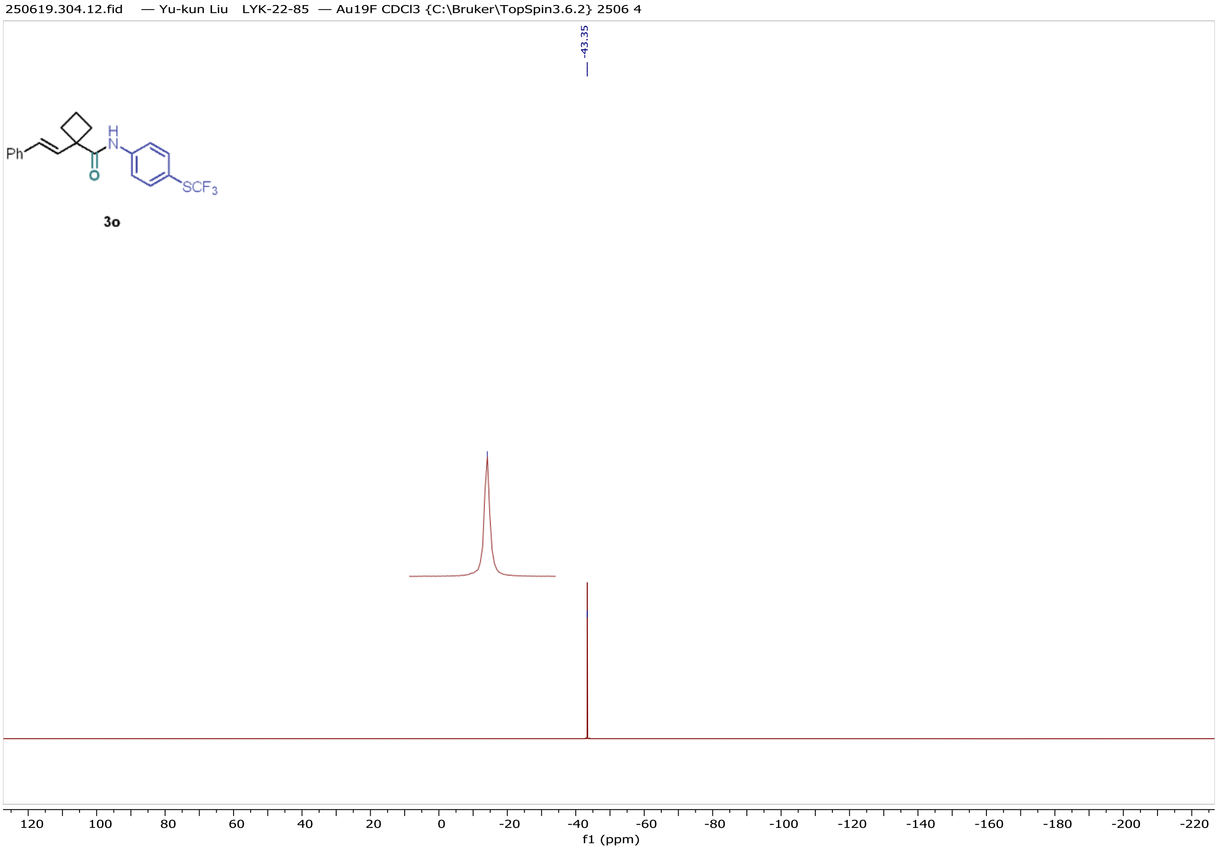

^{1}H NMR spectrum of **3p** (300 MHz, $CDCl_3$)

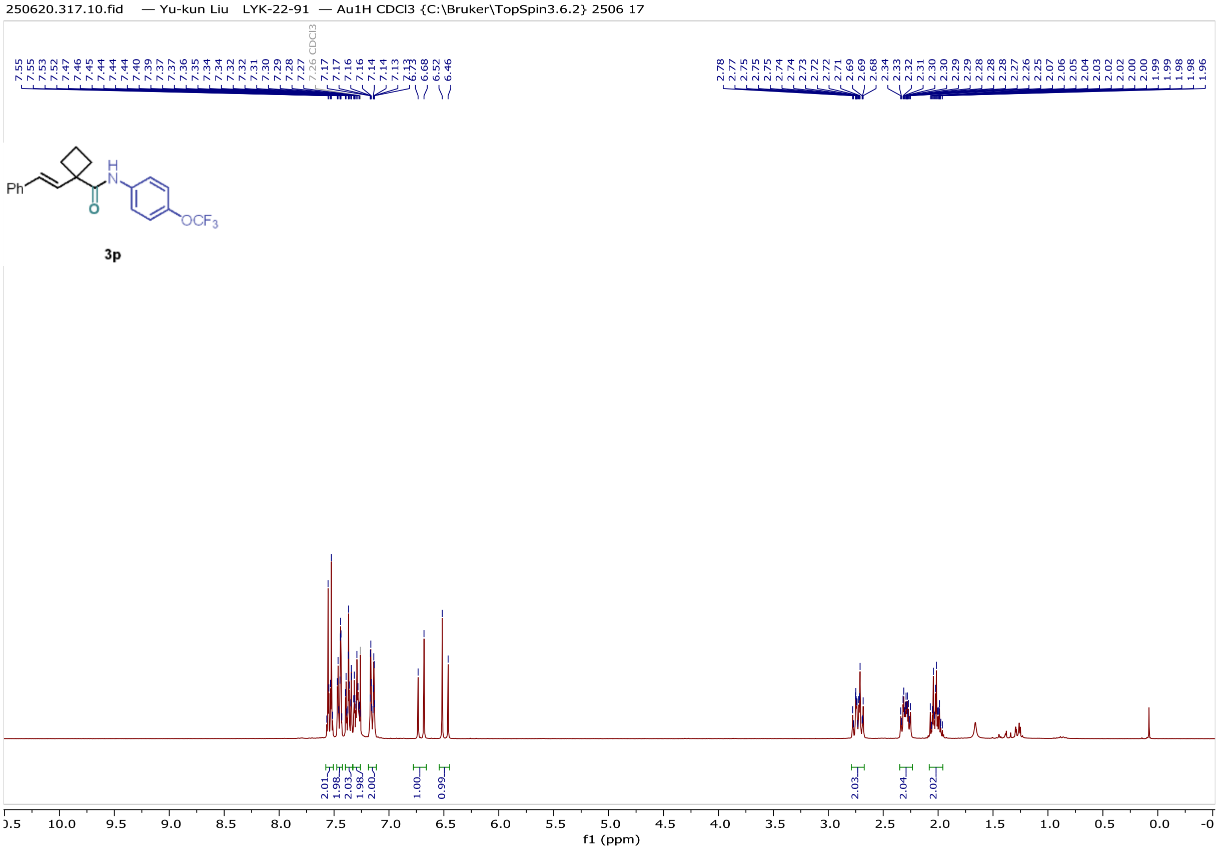

^{13}C NMR spectrum of **3p** (75 MHz, $CDCl_3$)

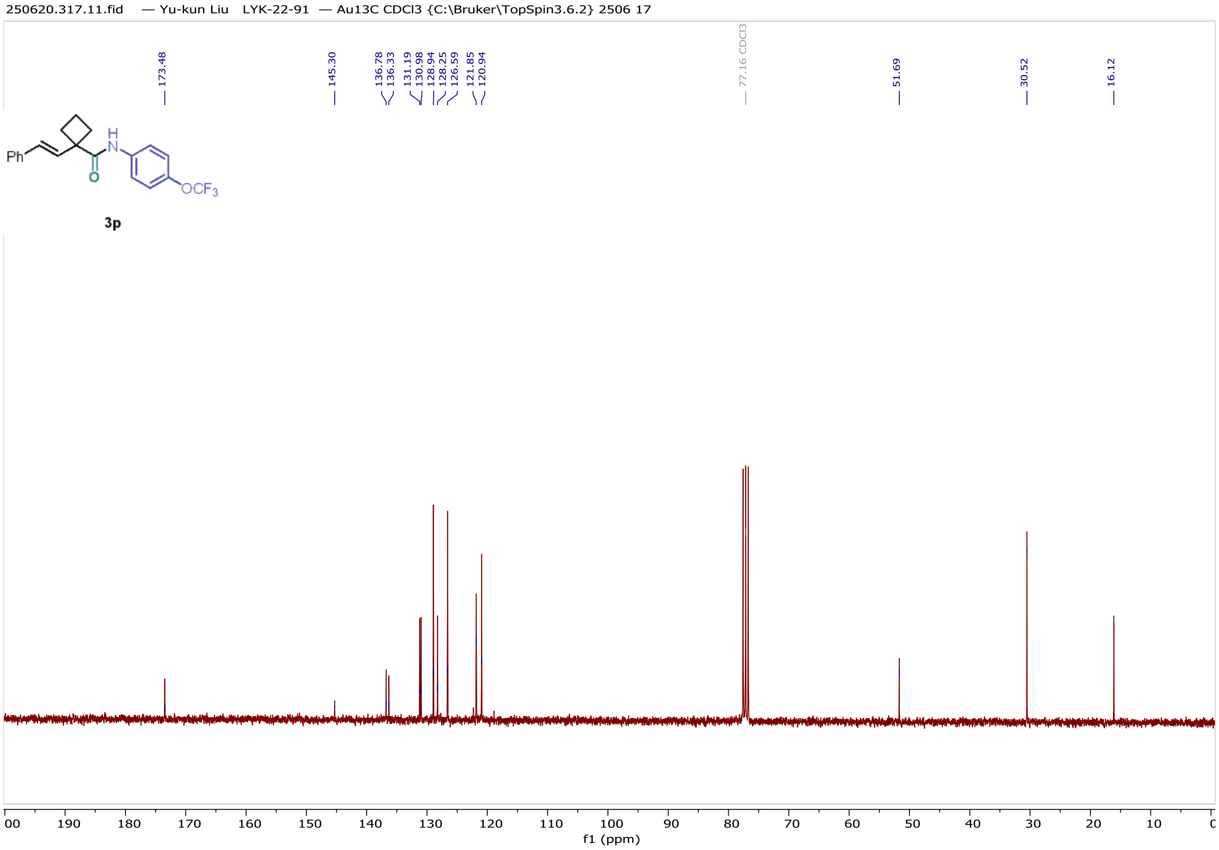

^{19}F NMR spectrum of **3p** (282 MHz, $CDCl_3$)

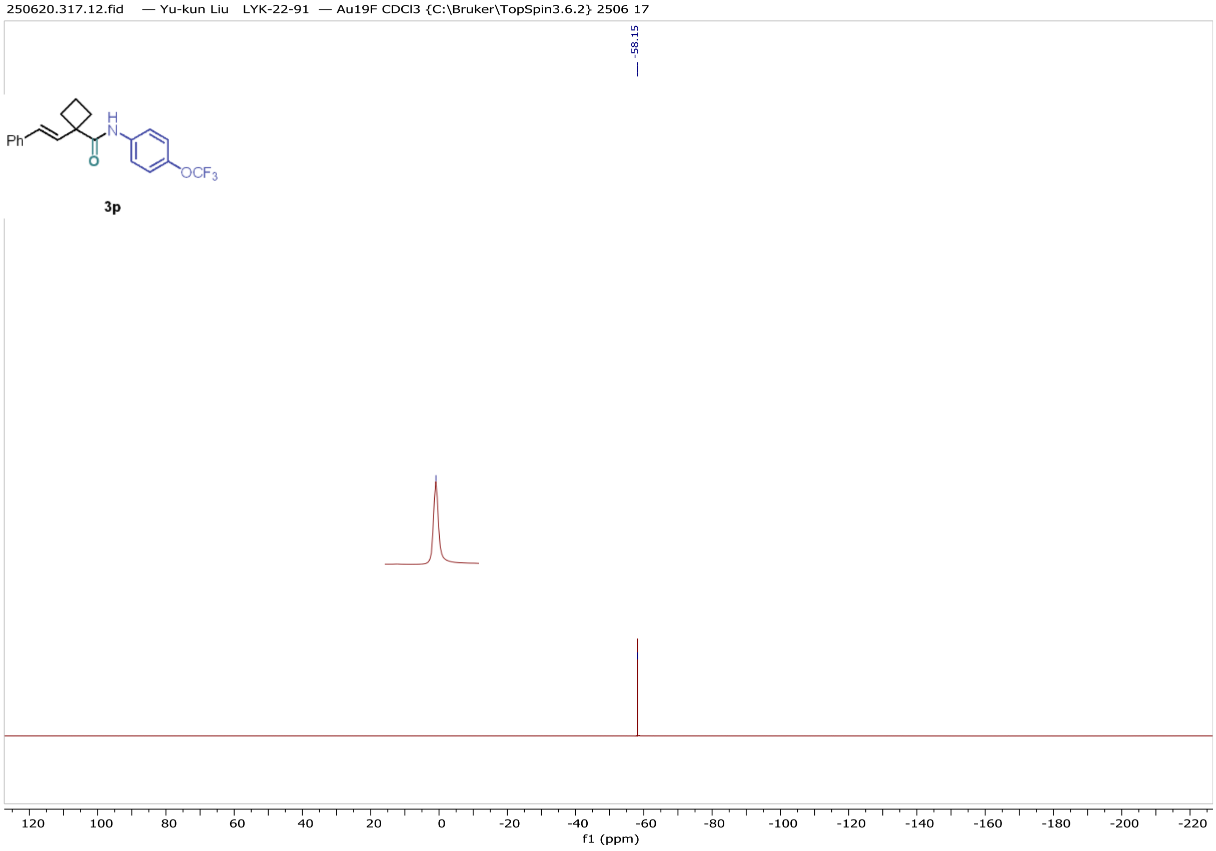

^{1}H NMR spectrum of **3q** (300 MHz, $CDCl_3$)

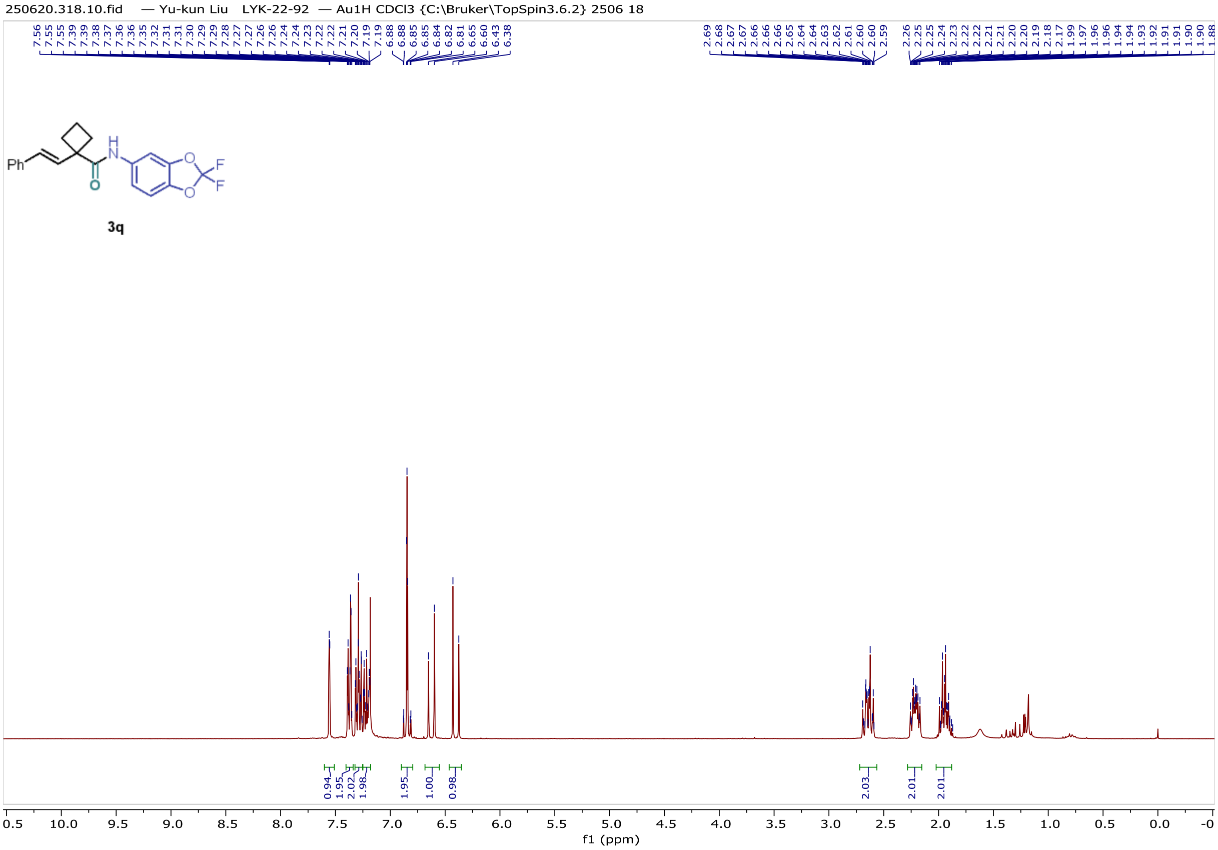

^{13}C NMR spectrum of **3q** (75 MHz, $CDCl_3$)

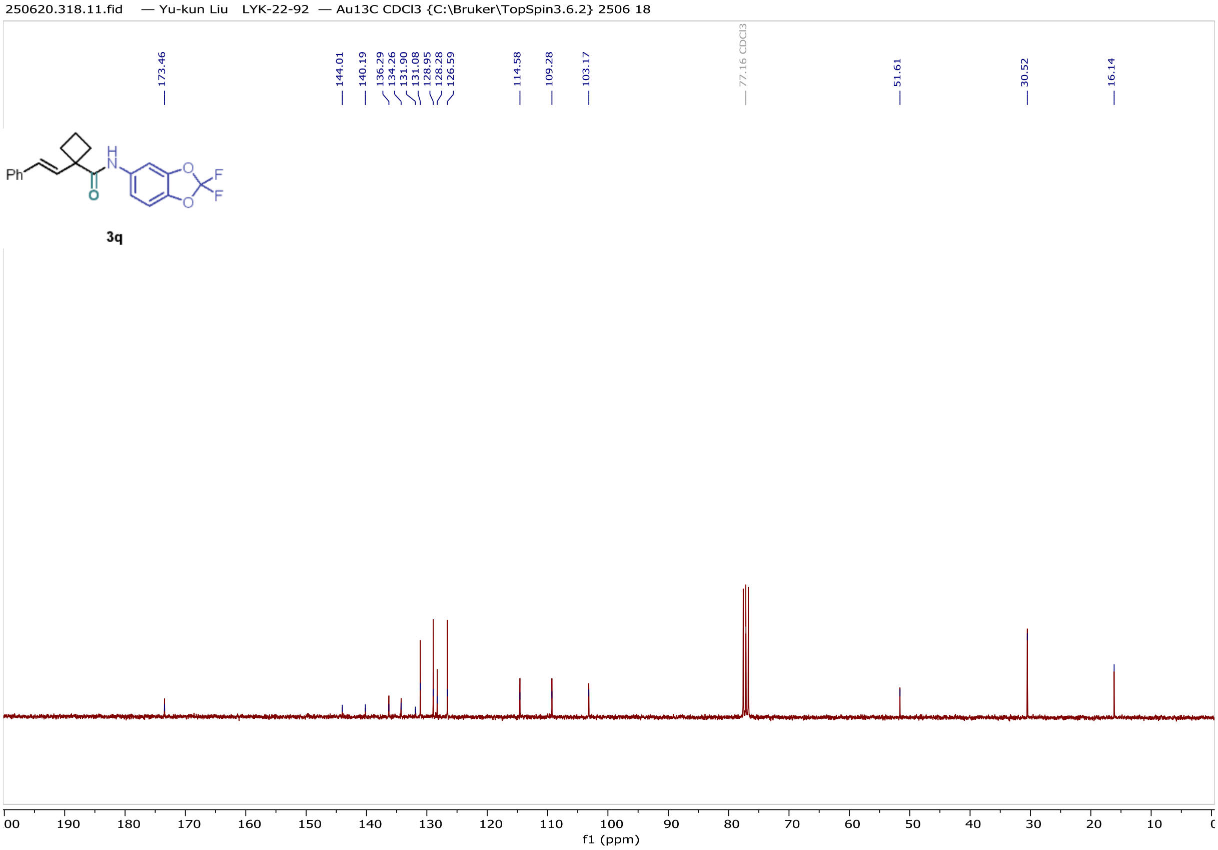

^{19}F NMR spectrum of **3q** (282 MHz, $CDCl_3$)

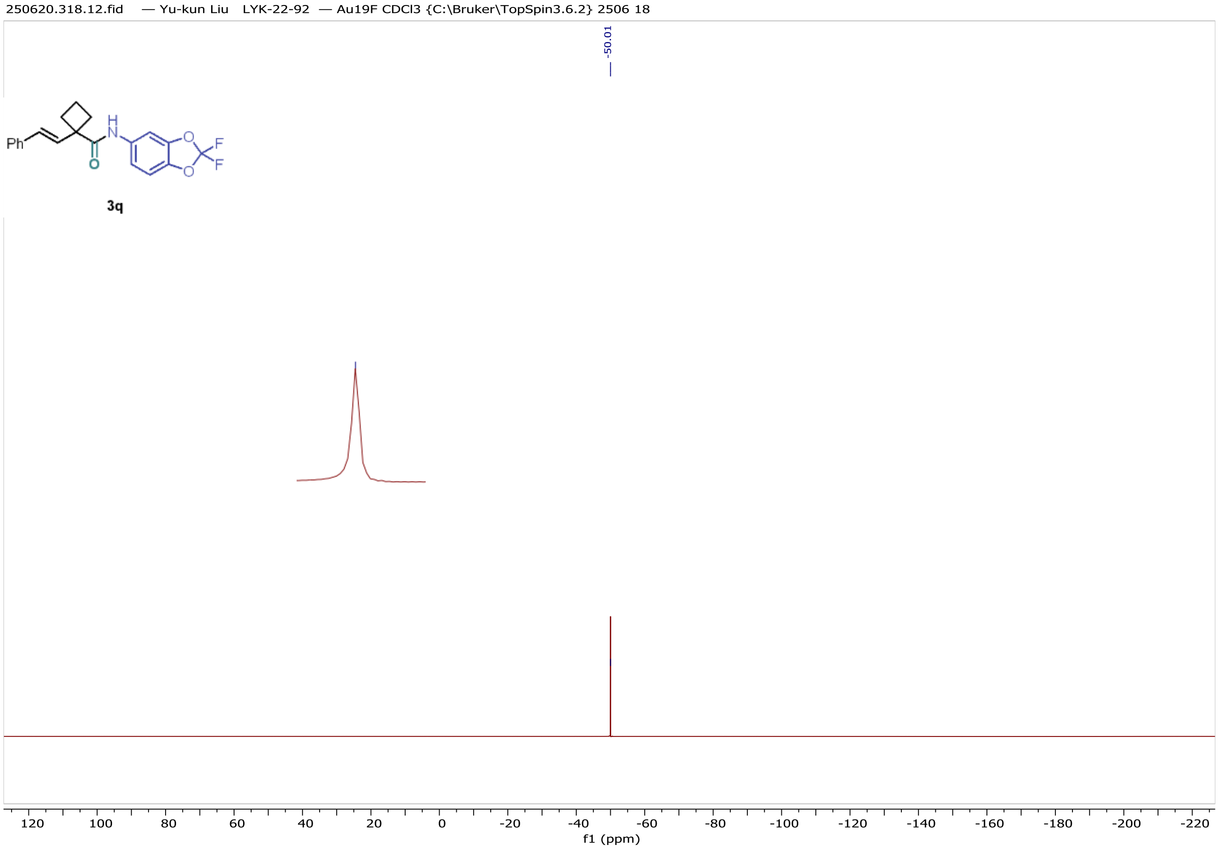

^{1}H NMR spectrum of **3r** (300 MHz, $CDCl_3$)

250620.320.10.fid — Yu-kun Liu LYK-22-94 — Au1H CDCl3 {C:\Bruker\TopSpin3.6.2} 2506 20

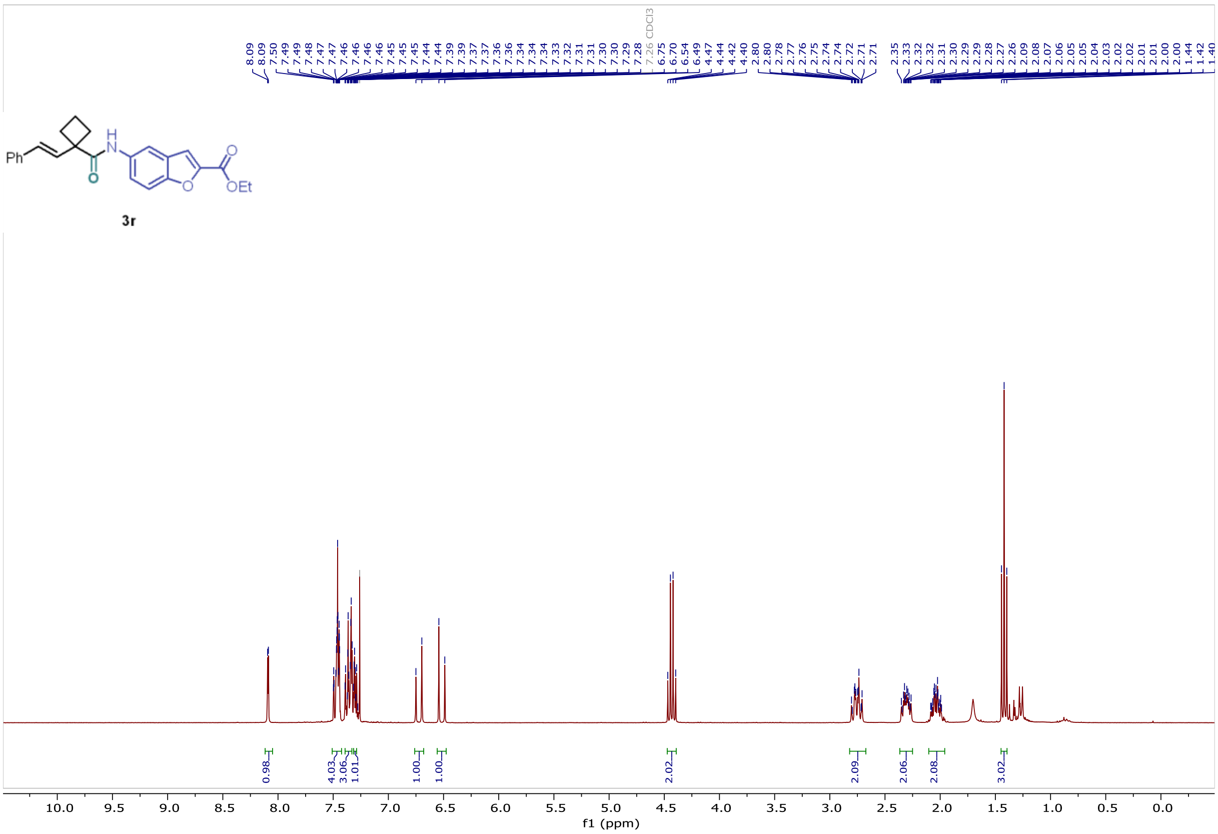

^{13}C NMR spectrum of **3r** (75 MHz, $CDCl_3$)

250620.320.11.fid — Yu-kun Liu LYK-22-94 — Au13C CDCl3 {C:\Bruker\TopSpin3.6.2} 2506 20

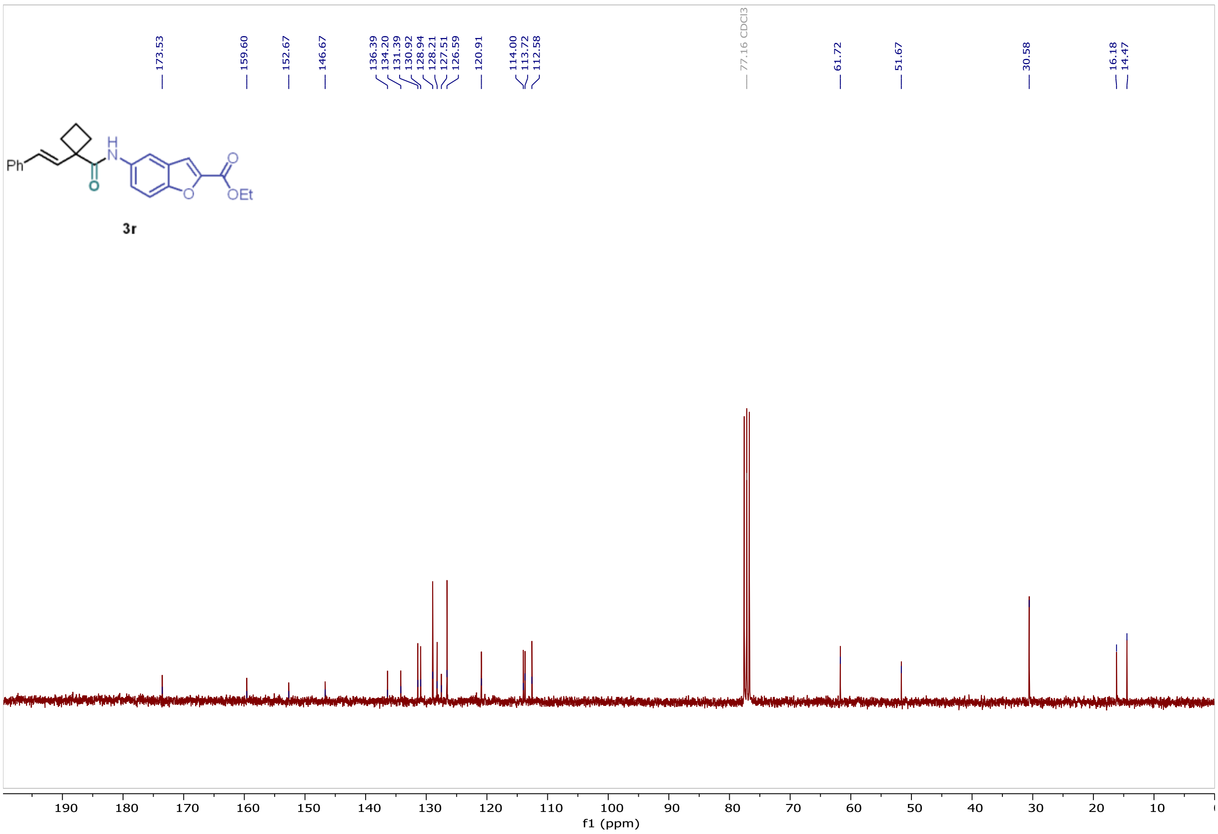

^{1}H NMR spectrum of **3s** (400 MHz, $CDCl_3$)

250624.411.10.fid — Yu-kun Liu LYK-22-100 — Au1H CDCl3 {C:\Bruker\TopSpin3.6.2} 2506 11

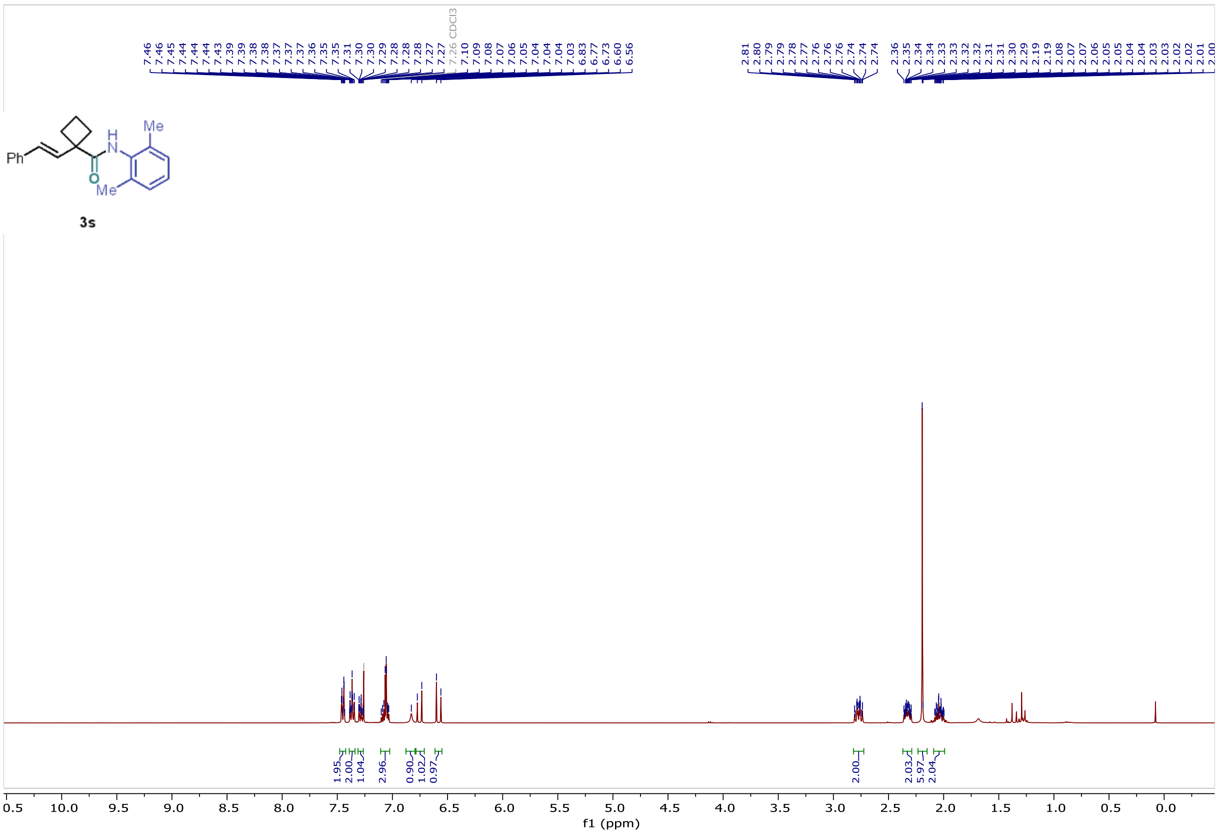

^{13}C NMR spectrum of **3s** (101 MHz, $CDCl_3$)

250624.411.11.fid — Yu-kun Liu LYK-22-100 — Au13C CDCl3 {C:\Bruker\TopSpin3.6.2} 2506 11

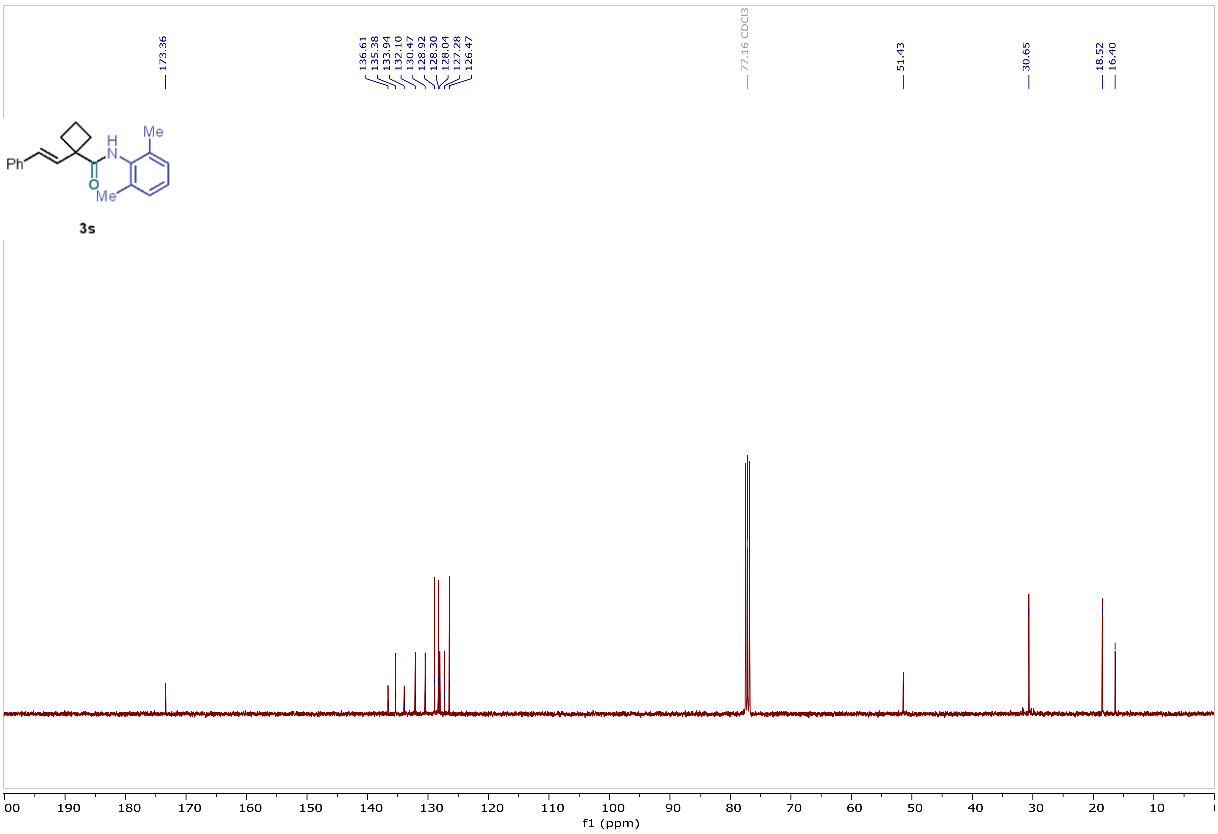

^{1}H NMR spectrum of **3t** (400 MHz, $CDCl_3$)

250624.412.10.fid — Yu-kun Liu LYK-22-101 — Au1H CDCl3 {C:\Bruker\TopSpin3.6.2} 2506 12

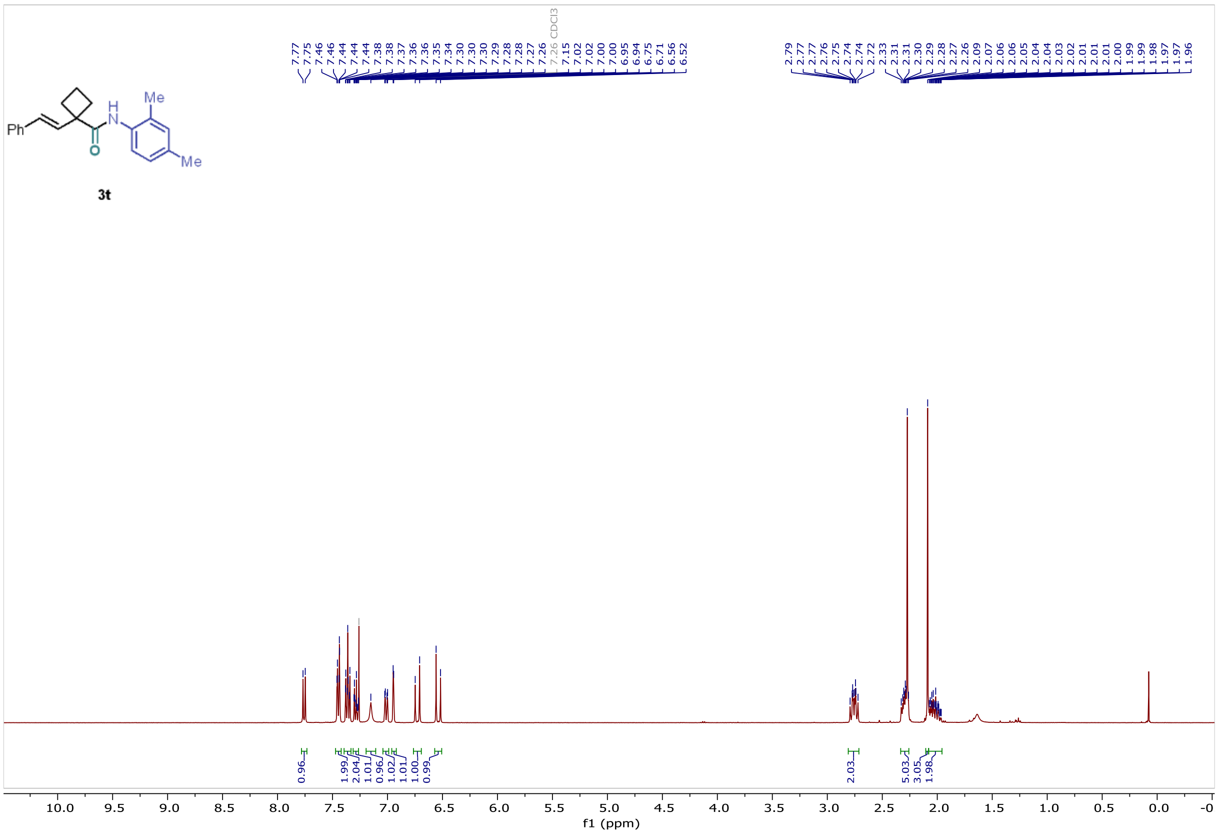

^{13}C NMR spectrum of **3t** (101 MHz, $CDCl_3$)

250624.412.11.fid — Yu-kun Liu LYK-22-101 — Au13C CDCl3 {C:\Bruker\TopSpin3.6.2} 2506 12

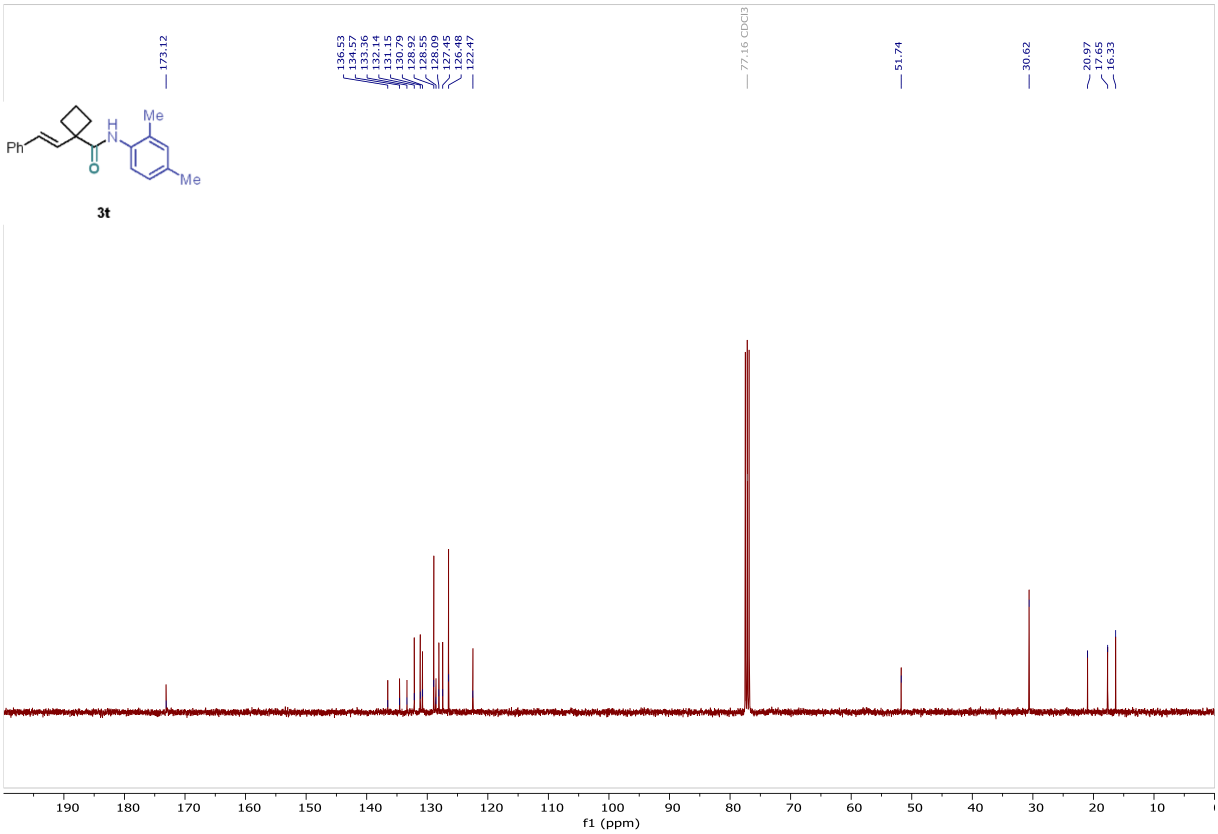

^{1}H NMR spectrum of **3u** (300 MHz, $CDCl_3$)

250625.305.10.fid — Yu-kun liu LYK-22-106 — Au1H CDCl3 {C:\Bruker\TopSpin3.6.2} 2506 5

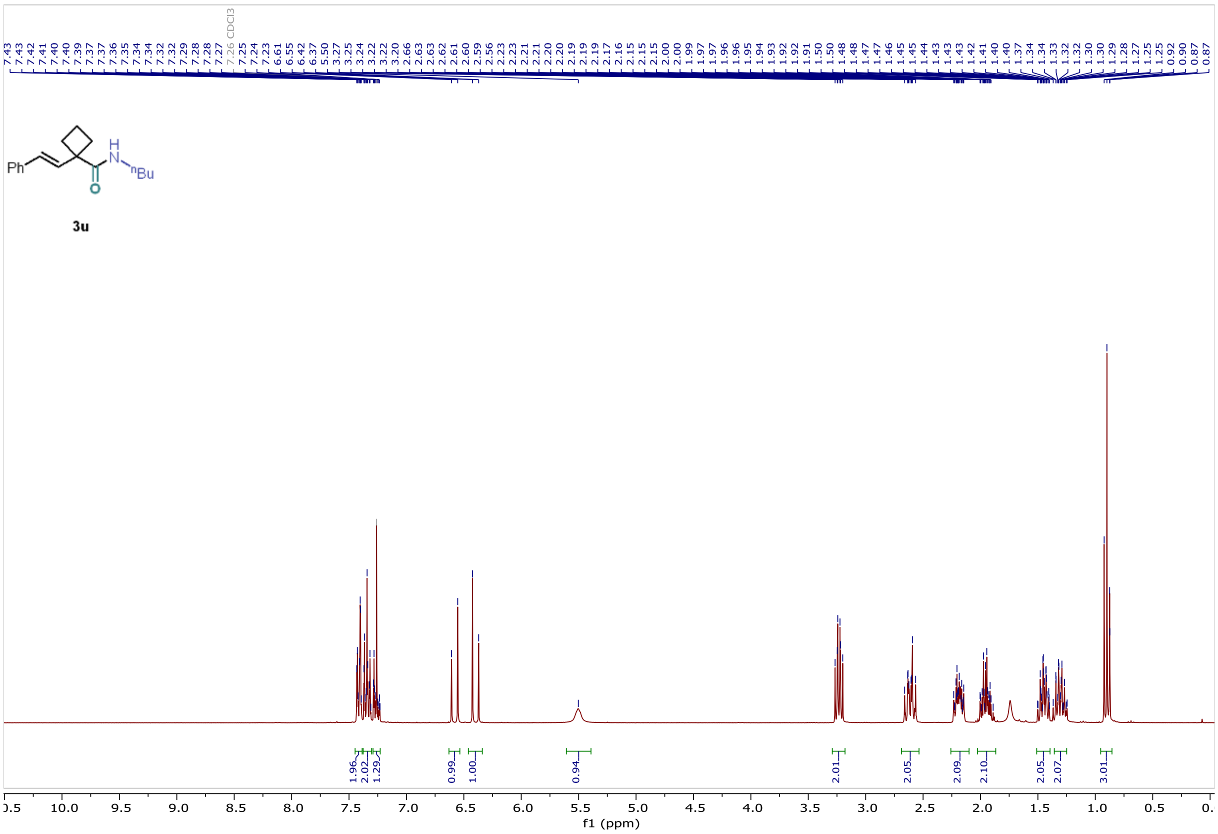

^{13}C NMR spectrum of **3u** (75 MHz, $CDCl_3$)

250625.305.11.fid — Yu-kun liu LYK-22-106 — Au13C CDCl3 {C:\Bruker\TopSpin3.6.2} 2506 5

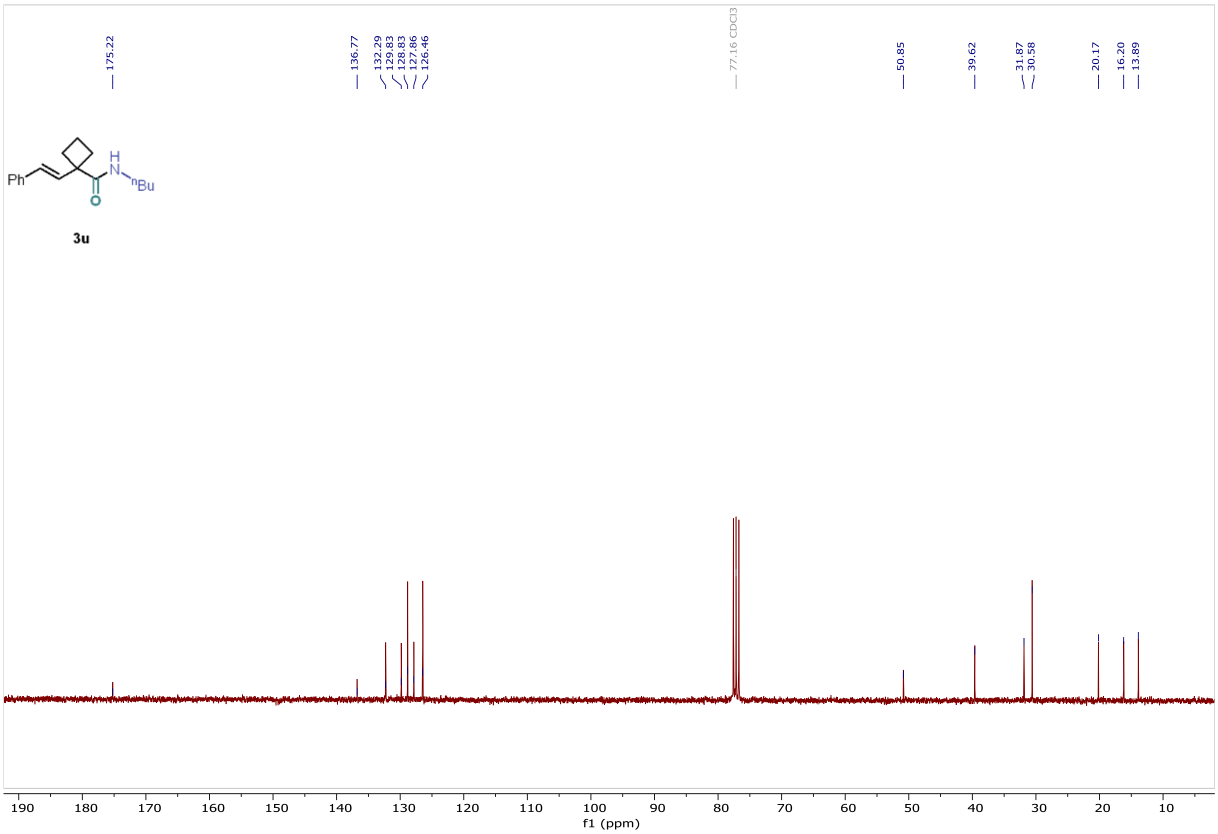

^{1}H NMR spectrum of **3v** (300 MHz, $CDCl_3$)

250625.306.10.fid — Yu-kun liu LYK-22-107 — Au1H CDCl3 {C:\Bruker\TopSpin3.6.2} 2506 6

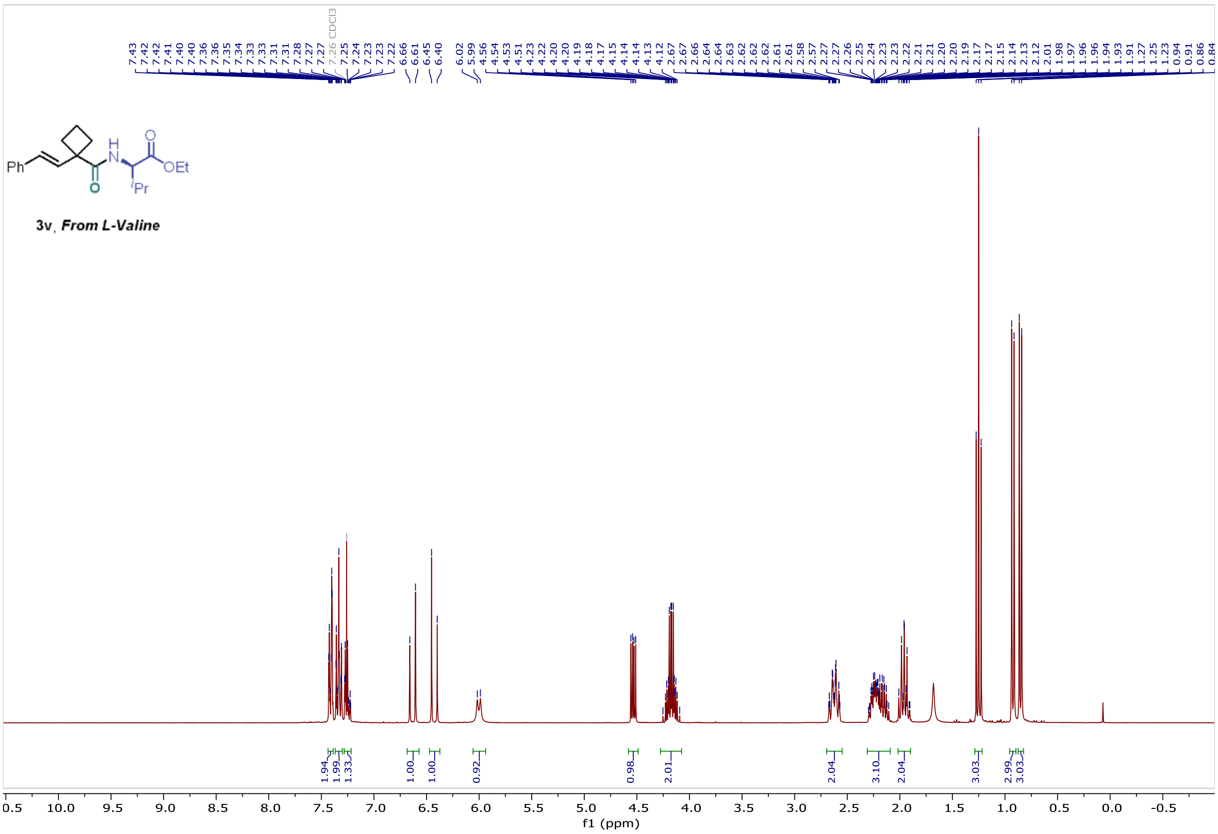

^{13}C NMR spectrum of **3v** (75 MHz, $CDCl_3$)

250625.306.11.fid — Yu-kun liu LYK-22-107 — Au13C CDCl3 {C:\Bruker\TopSpin3.6.2} 2506 6

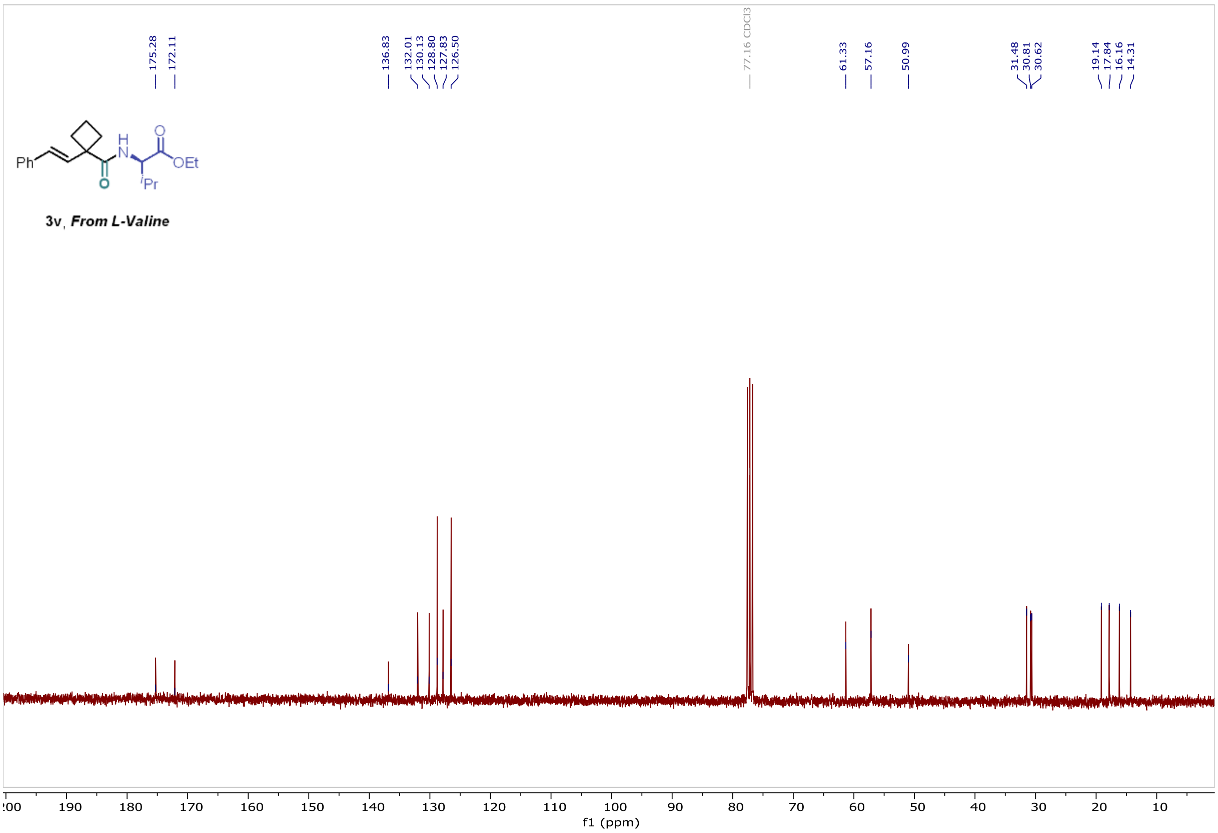

^{1}H NMR spectrum of **3w** (300 MHz, $CDCl_3$)

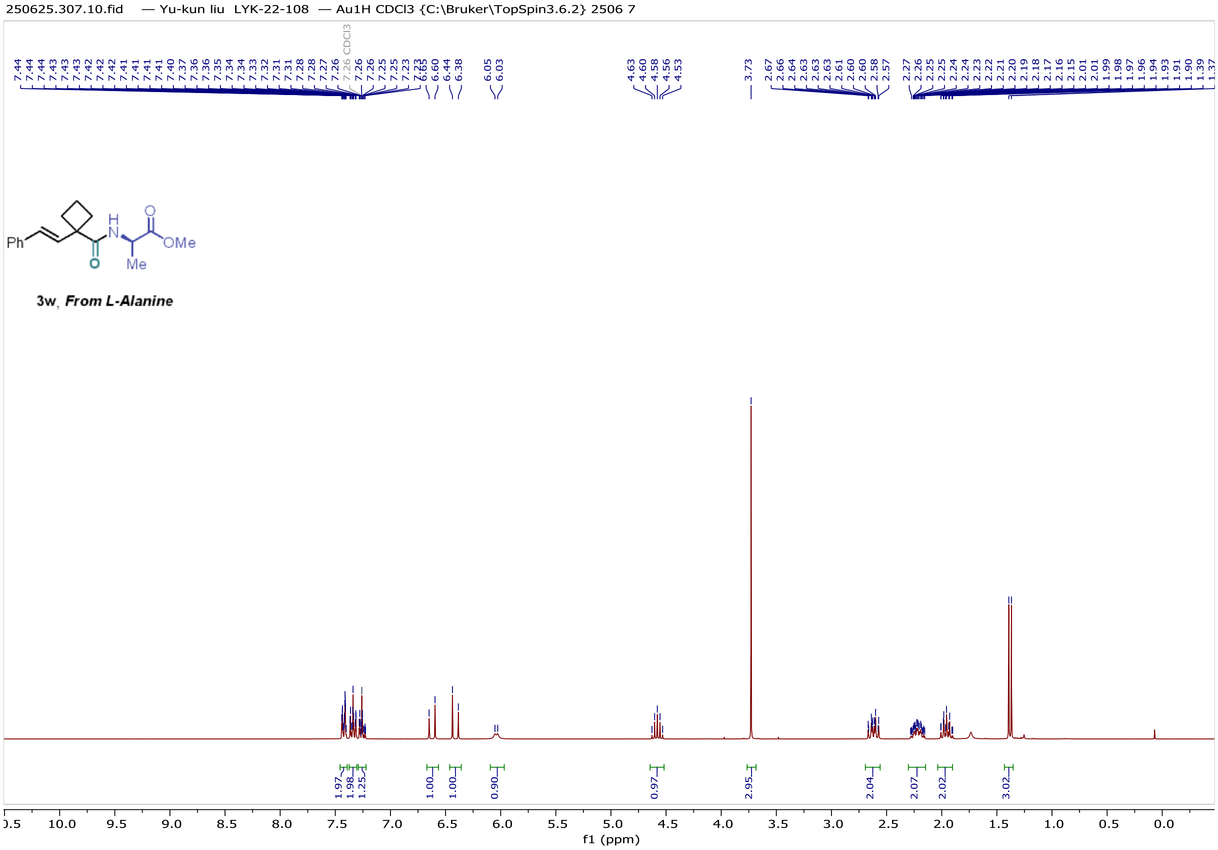

^{13}C NMR spectrum of **3w** (75 MHz, $CDCl_3$)

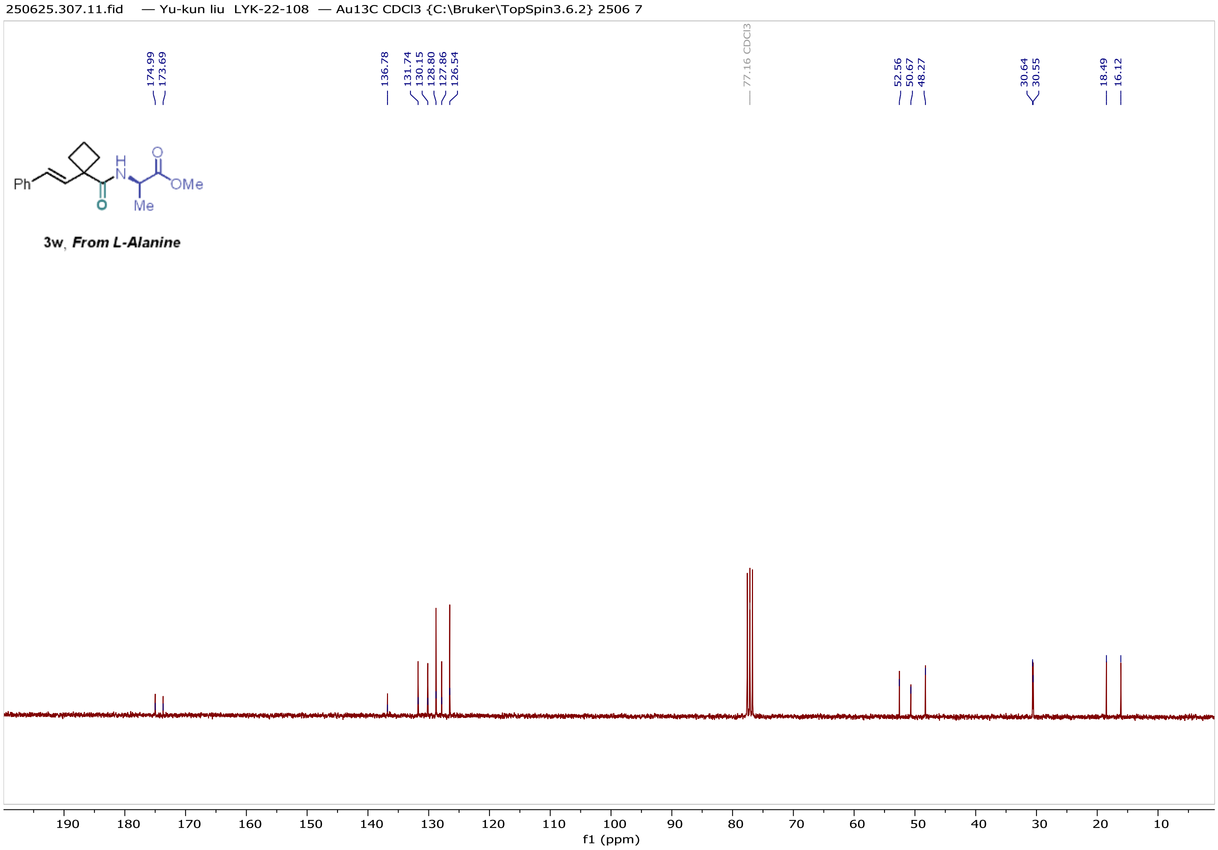

^{1}H NMR spectrum of **3x** (300 MHz, $CDCl_3$)

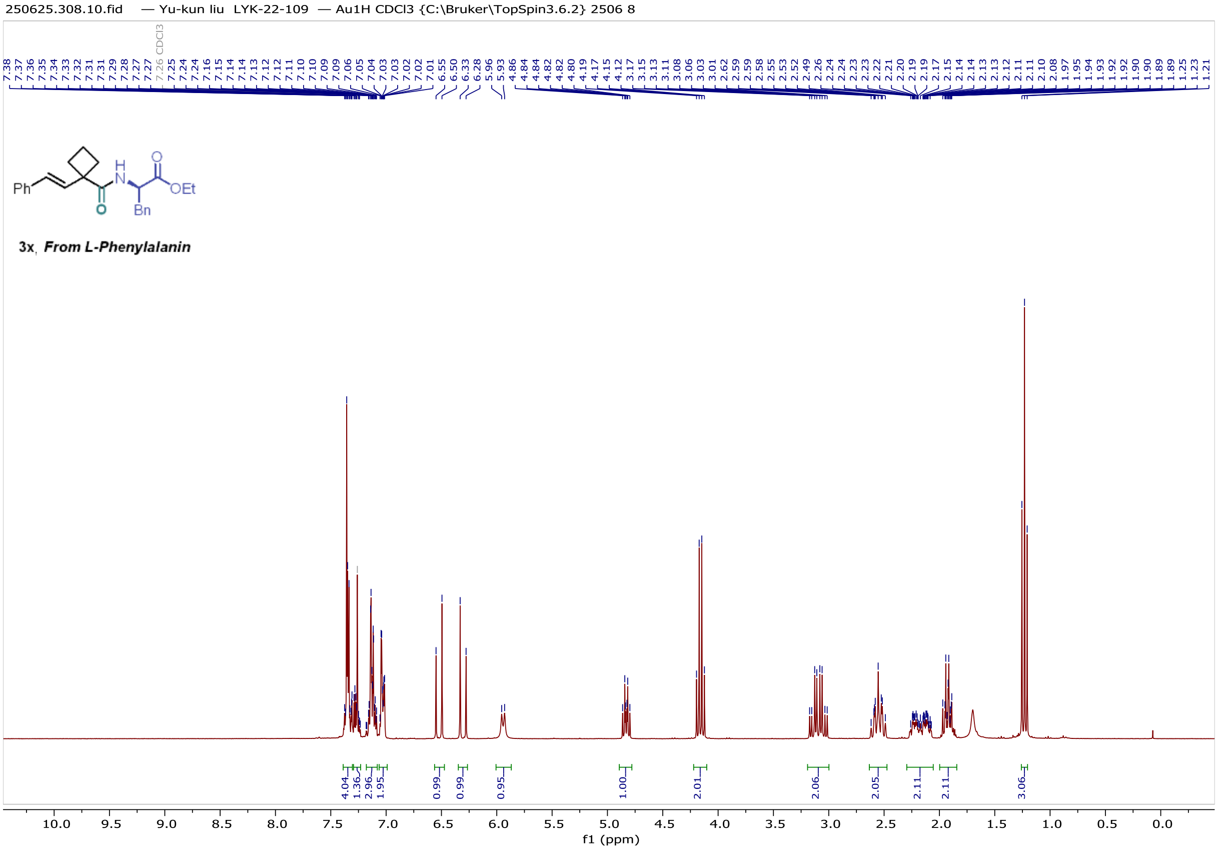

^{13}C NMR spectrum of **3x** (75 MHz, $CDCl_3$)

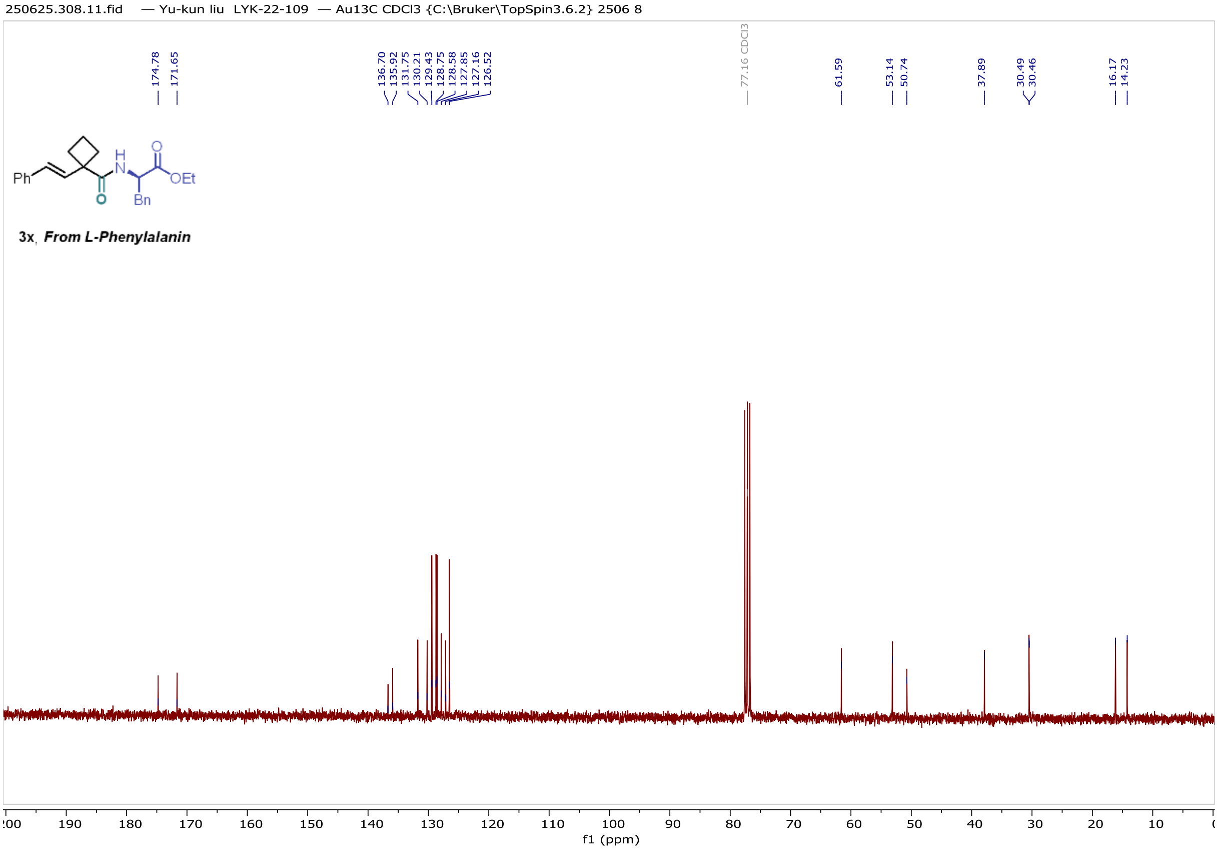

^{1}H NMR spectrum of **3y** (300 MHz, $CDCl_3$)

250625.309.10.fid — Yu-kun liu LYK-22-110 — Au1H CDCl3 {C:\Bruker\TopSpin3.6.2} 2506 9

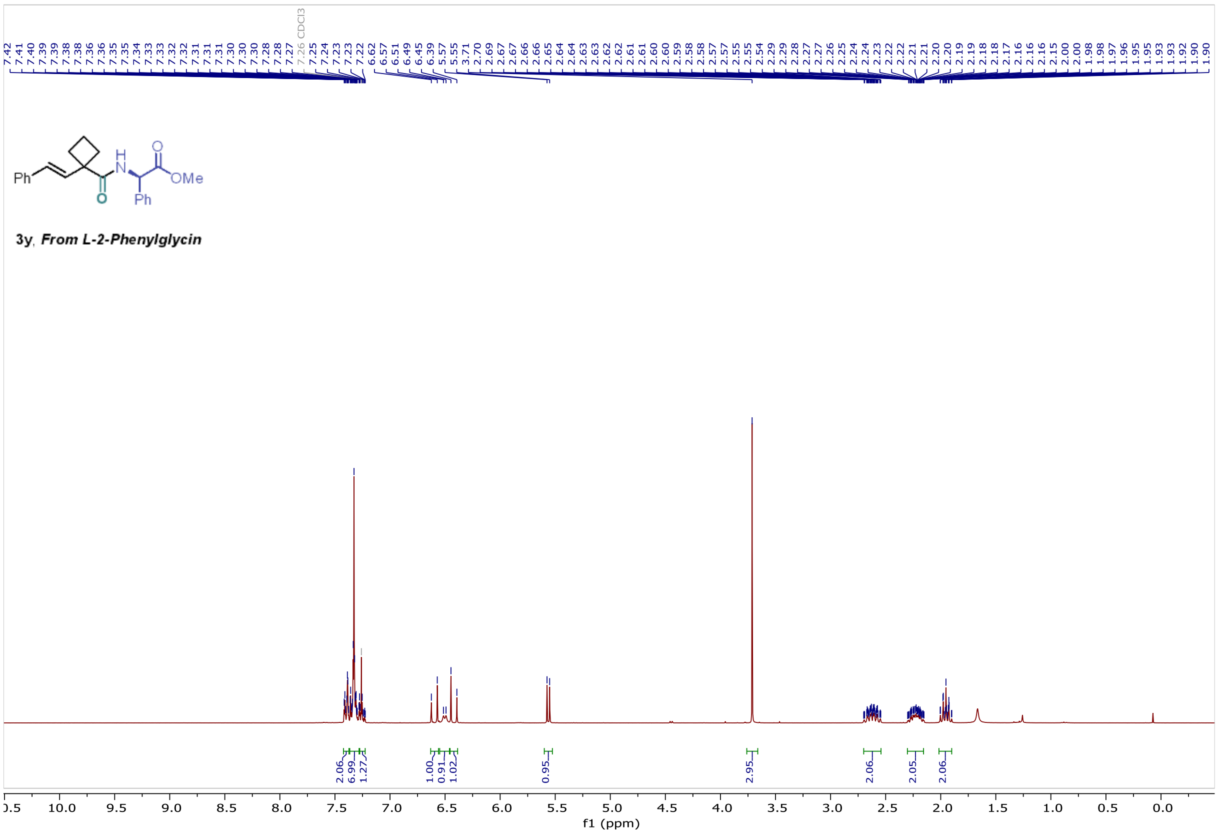

^{13}C NMR spectrum of **3y** (300 MHz, $CDCl_3$)

250625.309.11.fid — Yu-kun liu LYK-22-110 — Au13C CDCl3 {C:\Bruker\TopSpin3.6.2} 2506 9

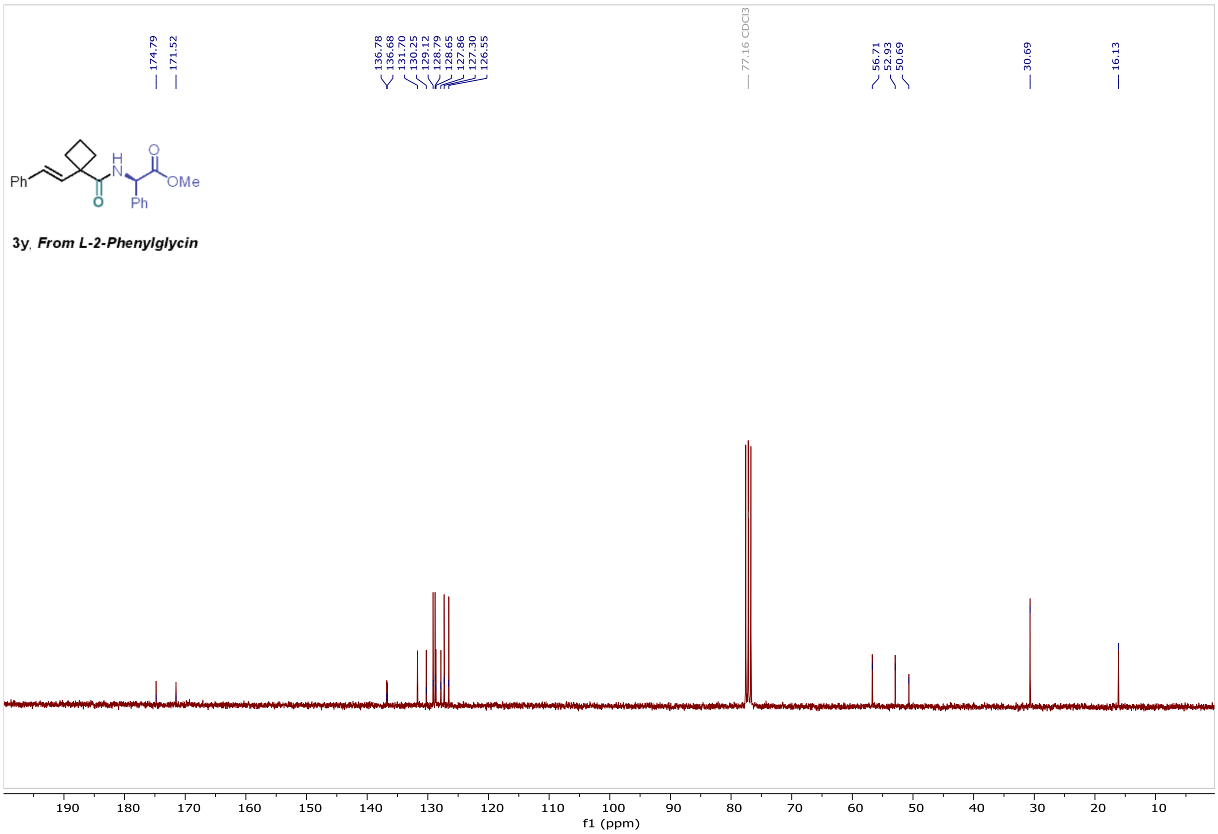

^{1}H NMR spectrum of **3z** (300 MHz, $CDCl_3$)

250626.301.10.fid — Yu-kun Liu LYK-22-111-re — Au1H CDCl3 {C:\Bruker\TopSpin3.6.2} 2506 1

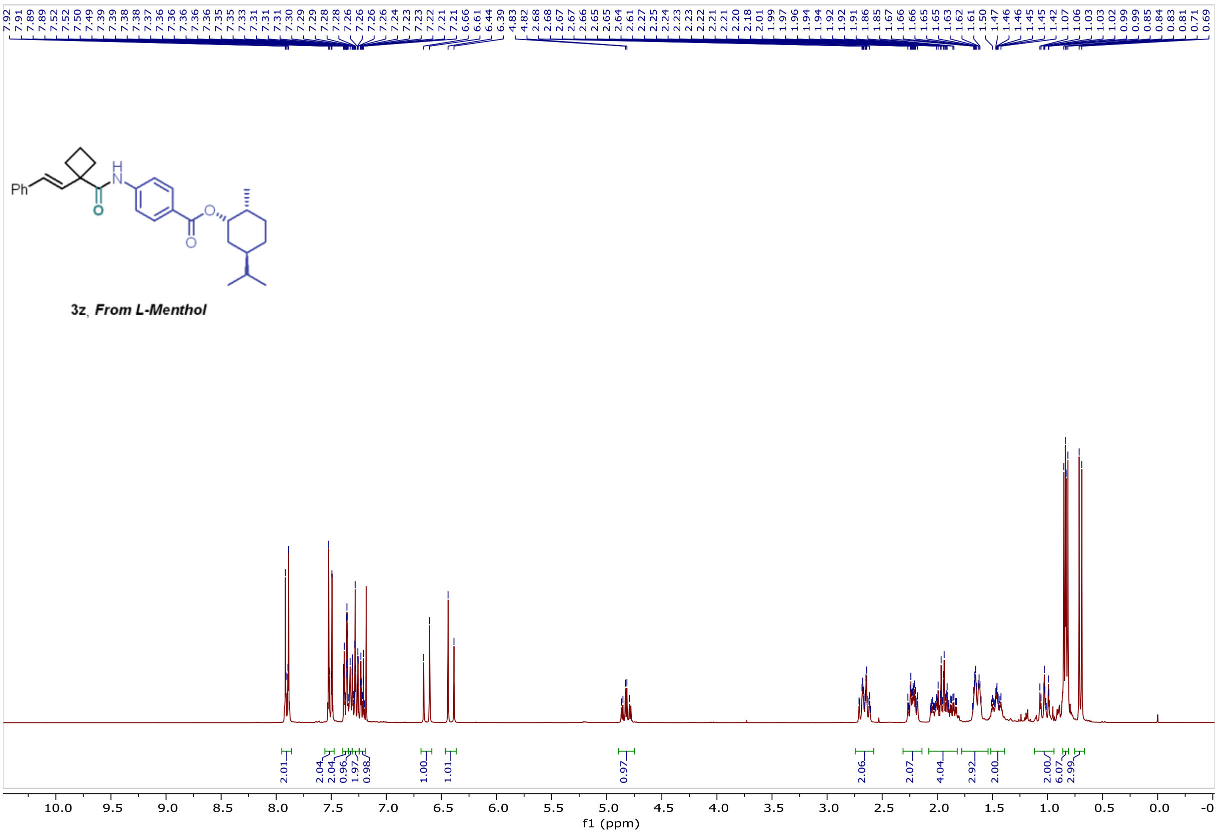

^{13}C NMR spectrum of **3z** (75 MHz, $CDCl_3$)

250626.301.11.fid — Yu-kun Liu LYK-22-111-re — Au13C CDCl3 {C:\Bruker\TopSpin3.6.2} 2506 1

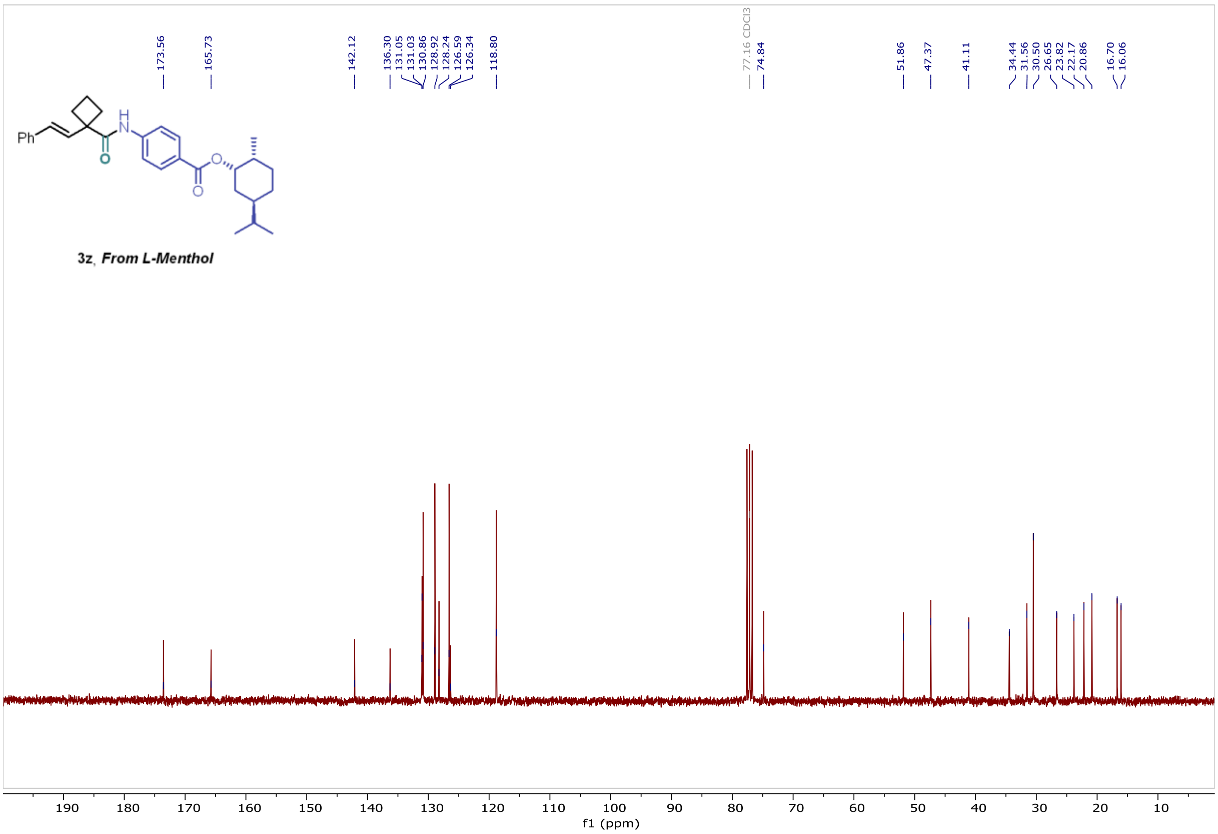

^{1}H NMR spectrum of **3ab** (300 MHz, $CDCl_3$)

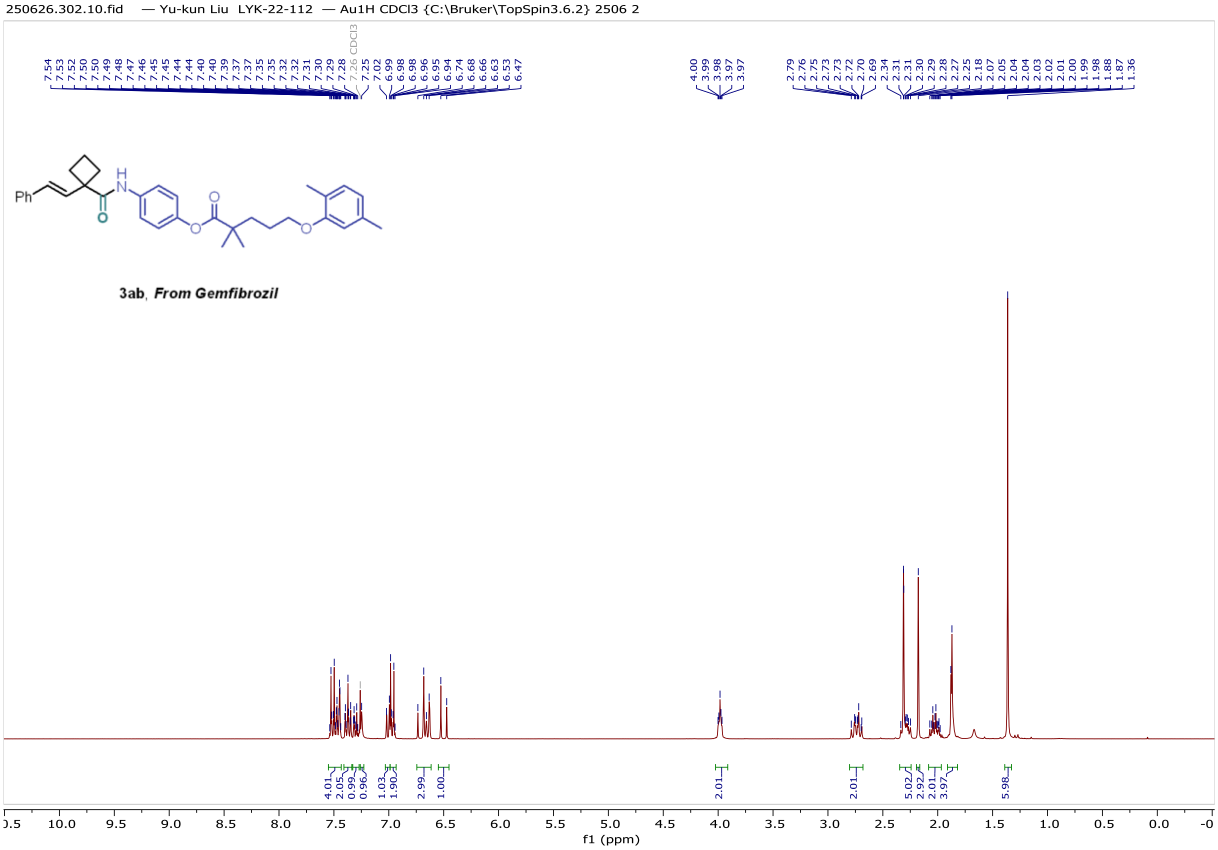

^{13}C NMR spectrum of **3ab** (75 MHz, $CDCl_3$)

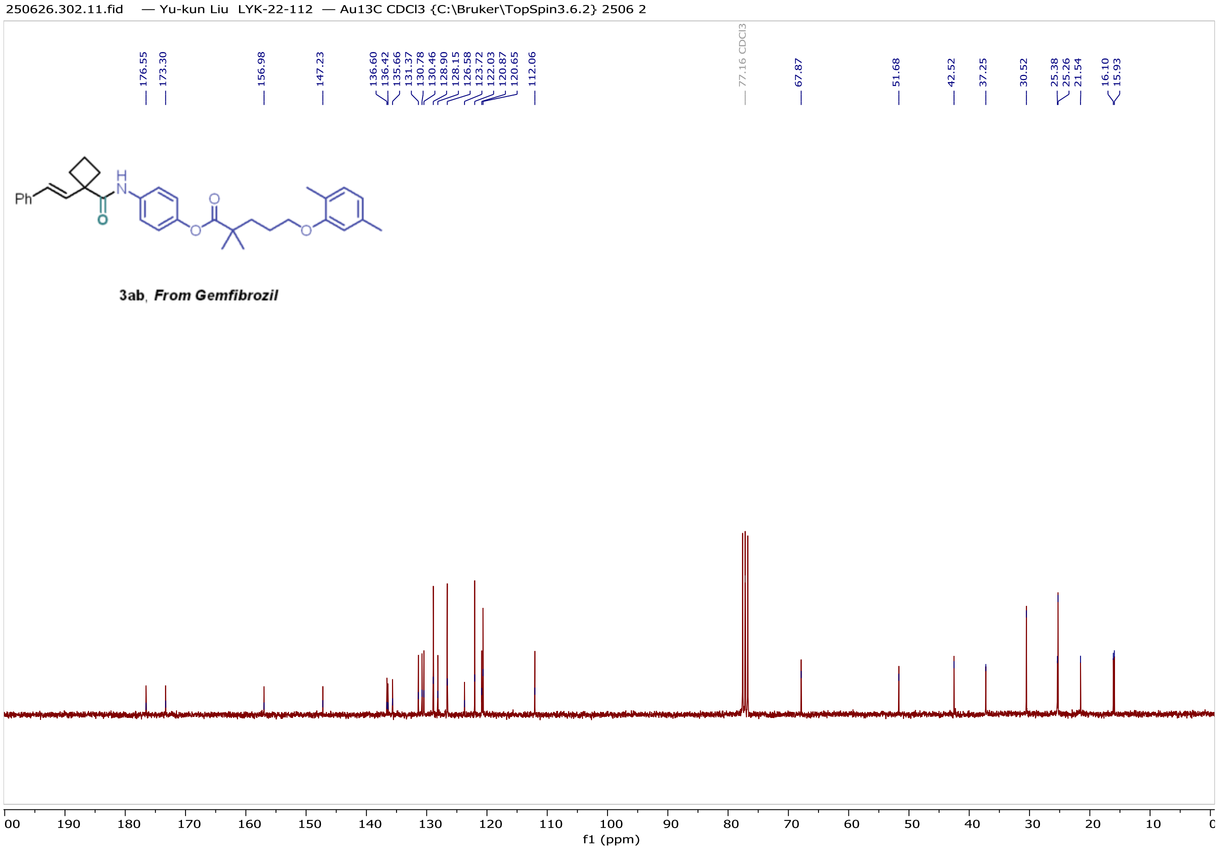

^{1}H NMR spectrum of **3ac** (300 MHz, $CDCl_3$)

250626.303.10.fid — Yu-kun Liu LYK-22-113 — Au1H CDCl3 {C:\Bruker\TopSpin3.6.2} 2506 3

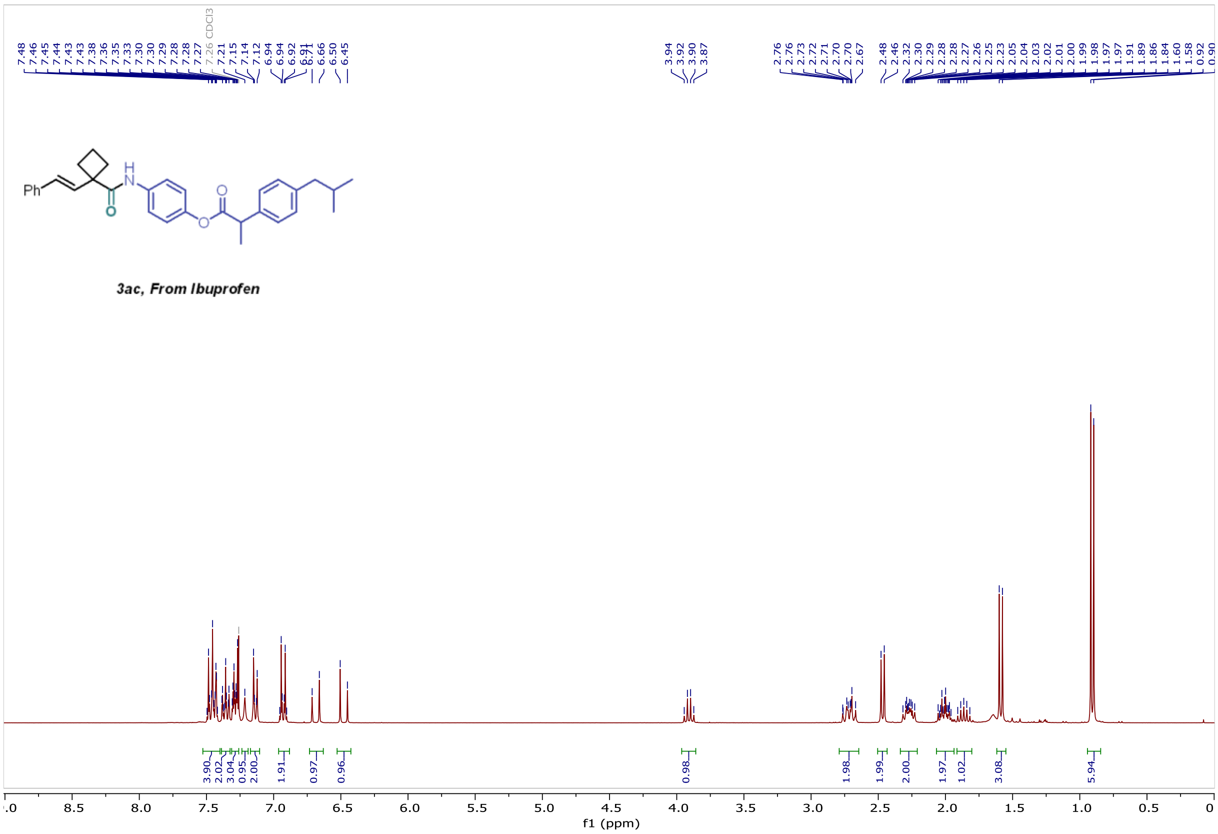

^{13}C NMR spectrum of **3ac** (75 MHz, $CDCl_3$)

250626.303.11.fid — Yu-kun Liu LYK-22-113 — Au13C CDCl3 {C:\Bruker\TopSpin3.6.2} 2506 3

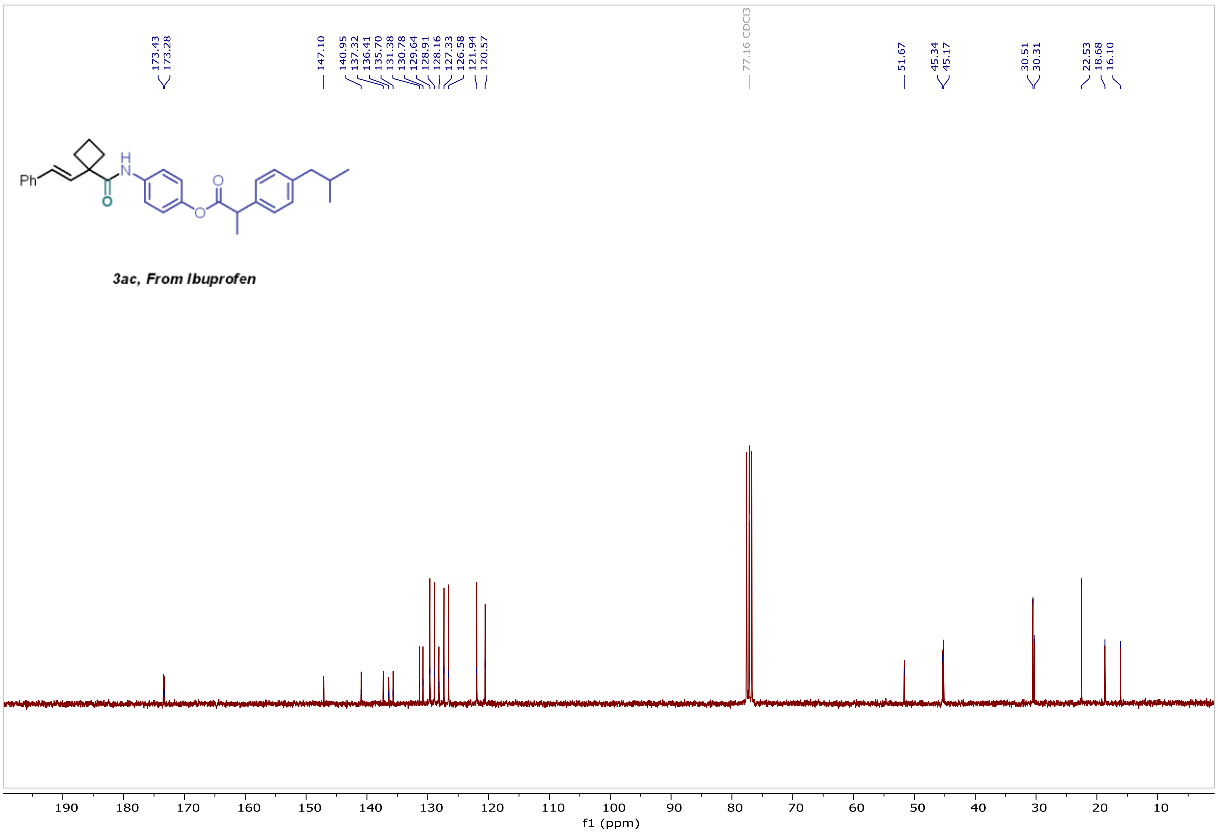

^{1}H NMR spectrum of **3ad** (400 MHz, $CDCl_3$)

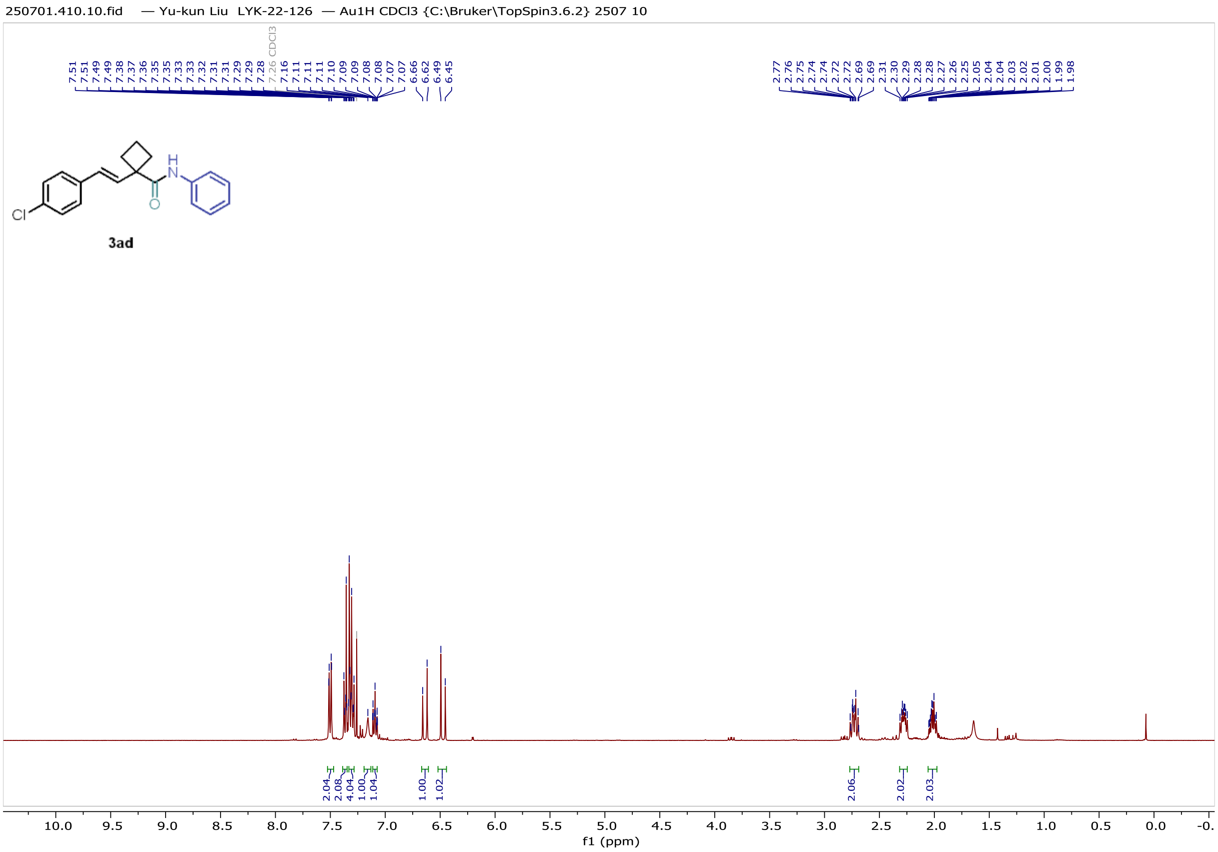

^{13}C NMR spectrum of **3ad** (101 MHz, $CDCl_3$)

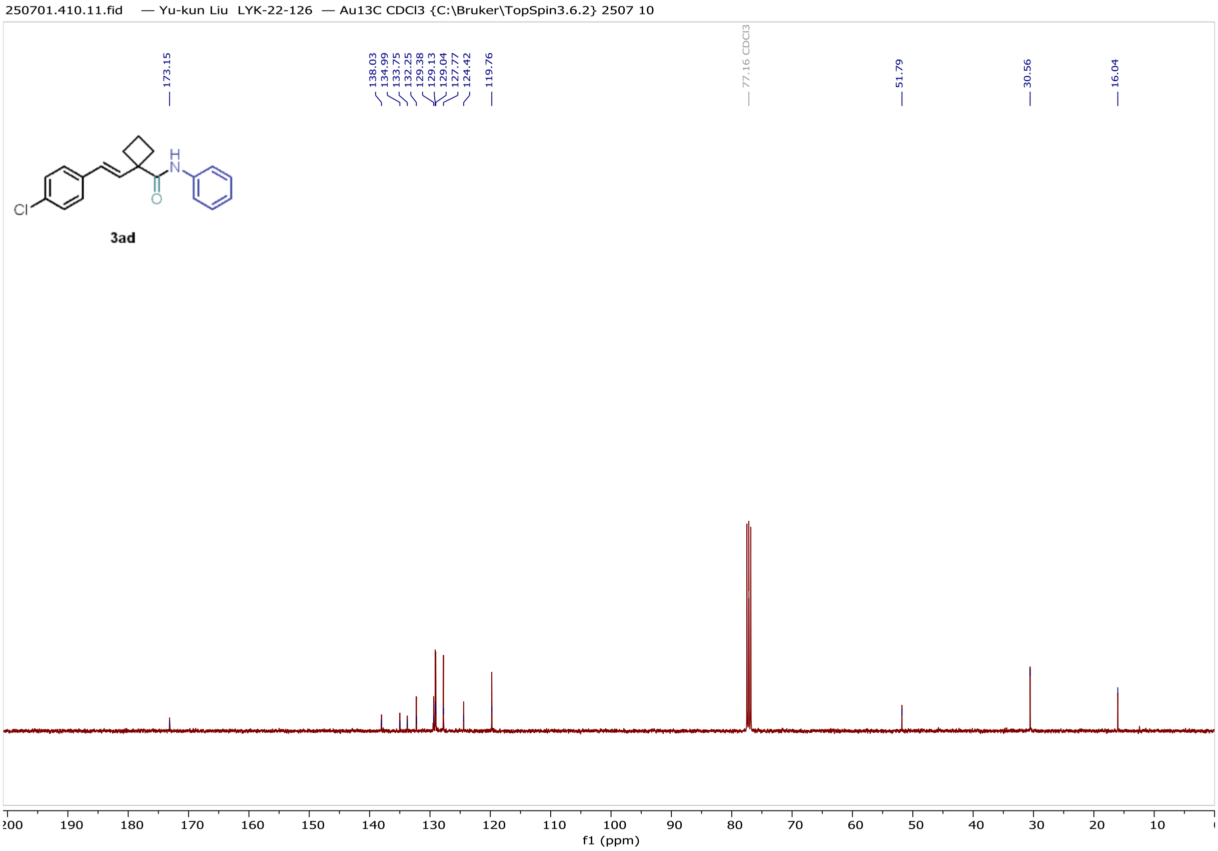

^{1}H NMR spectrum of **3ae** (400 MHz, $CDCl_3$)

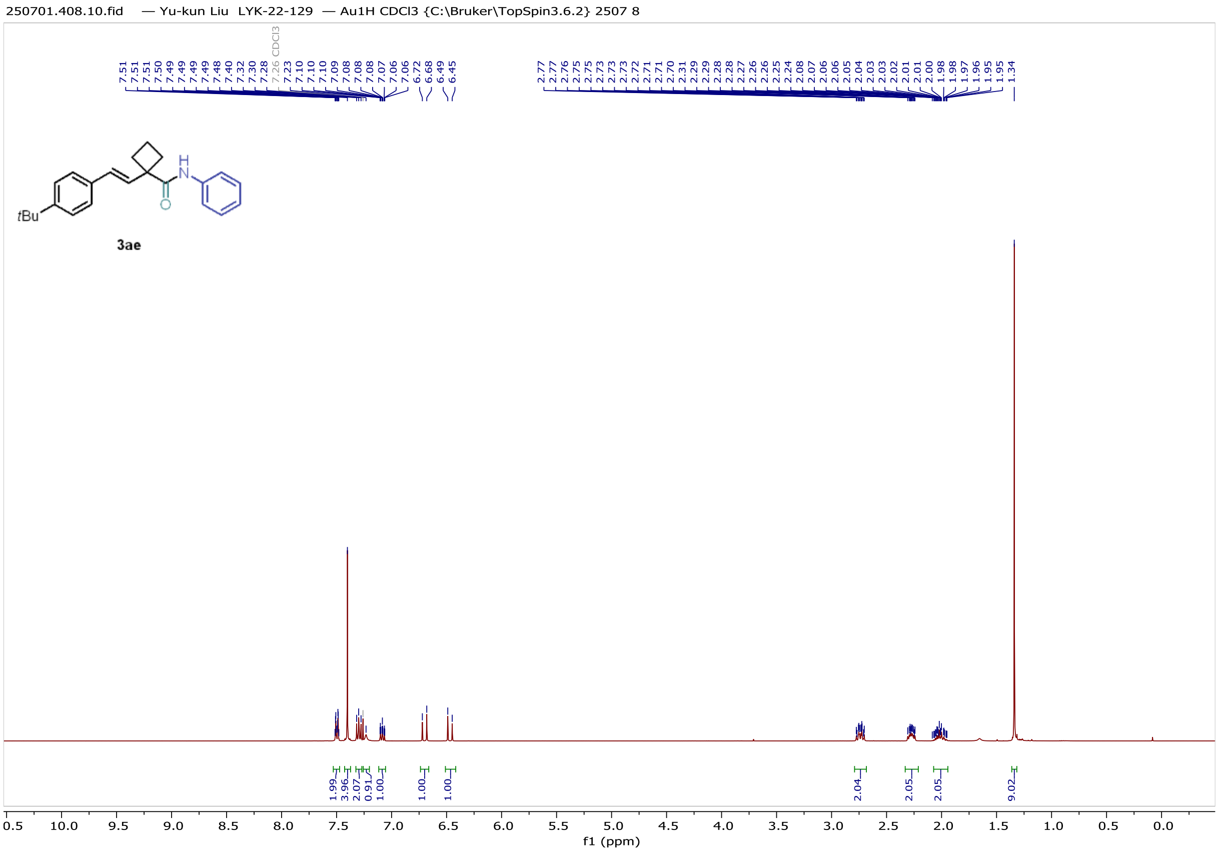

^{13}C NMR spectrum of **3ae** (101 MHz, $CDCl_3$)

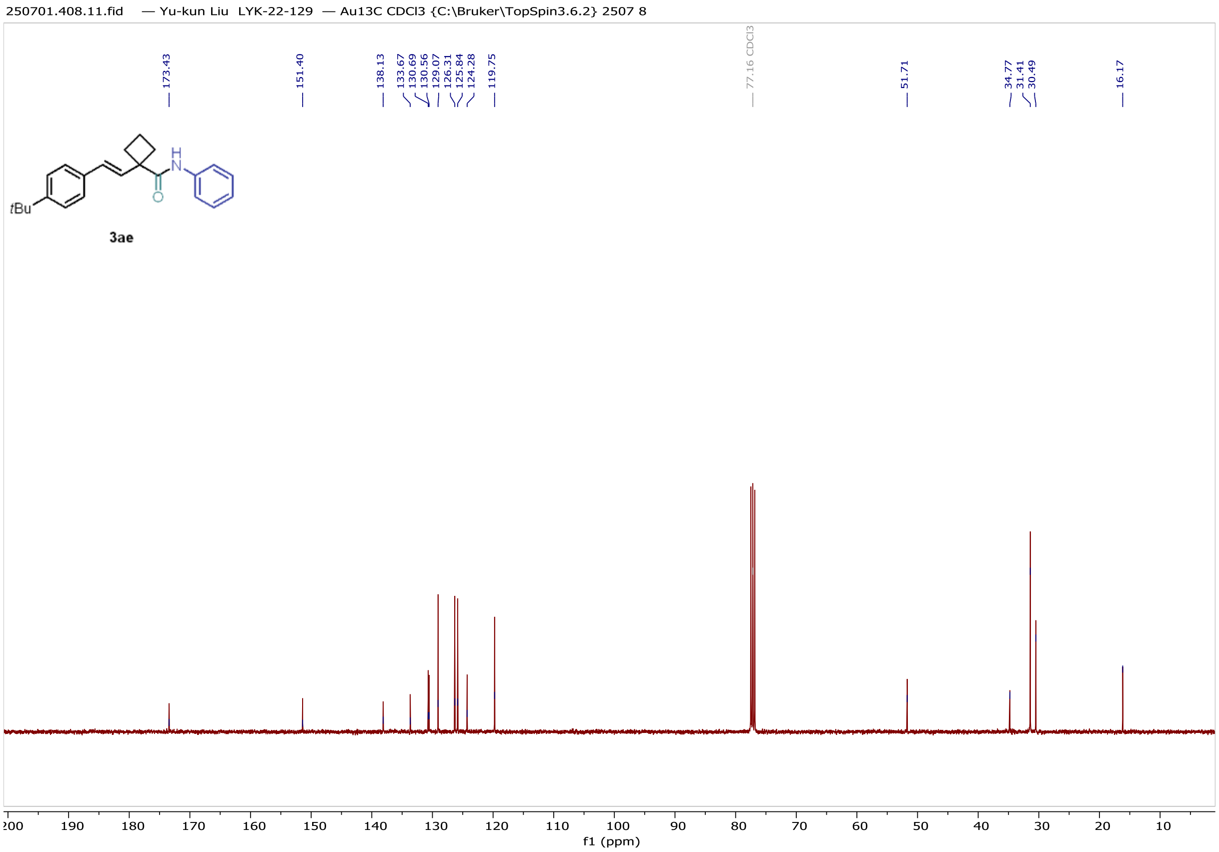

^{1}H NMR spectrum of **3af** (400 MHz, $CDCl_3$)

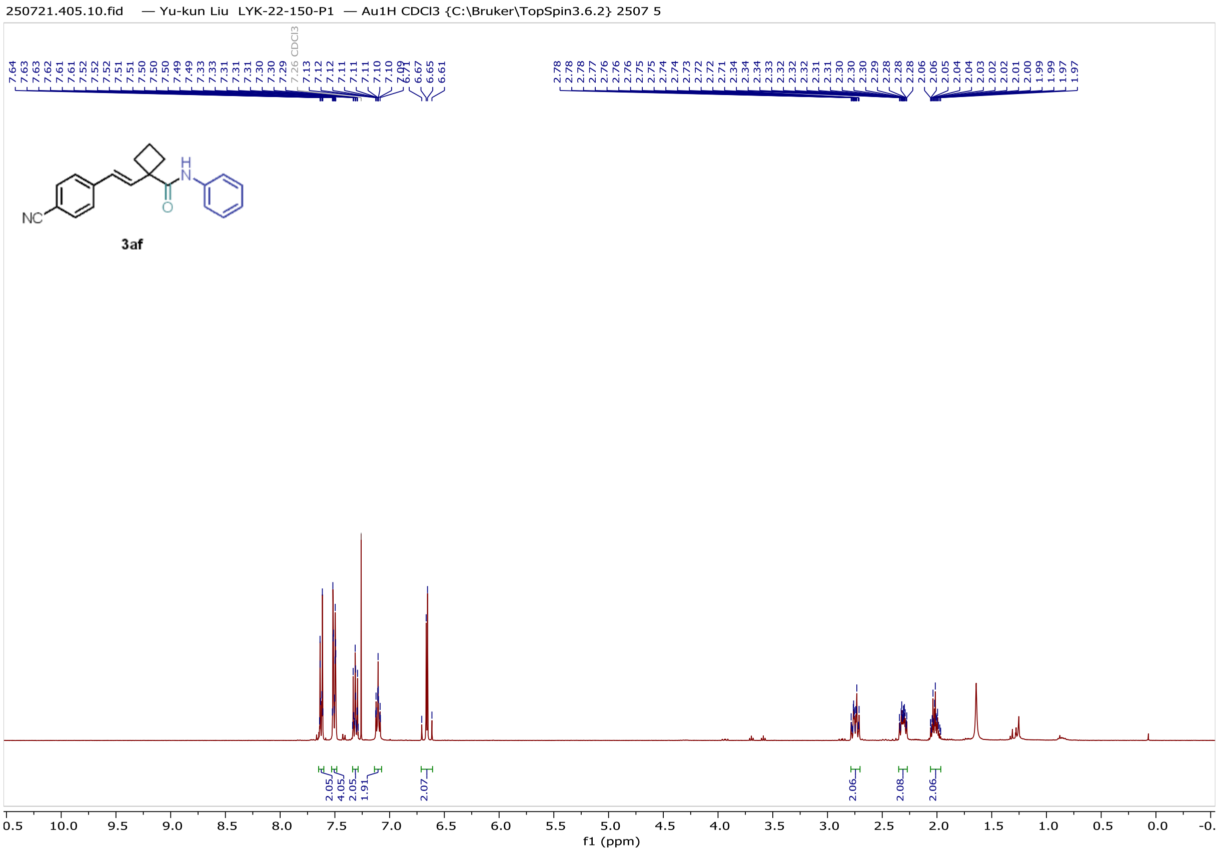

^{13}C NMR spectrum of **3af** (101 MHz, $CDCl_3$)

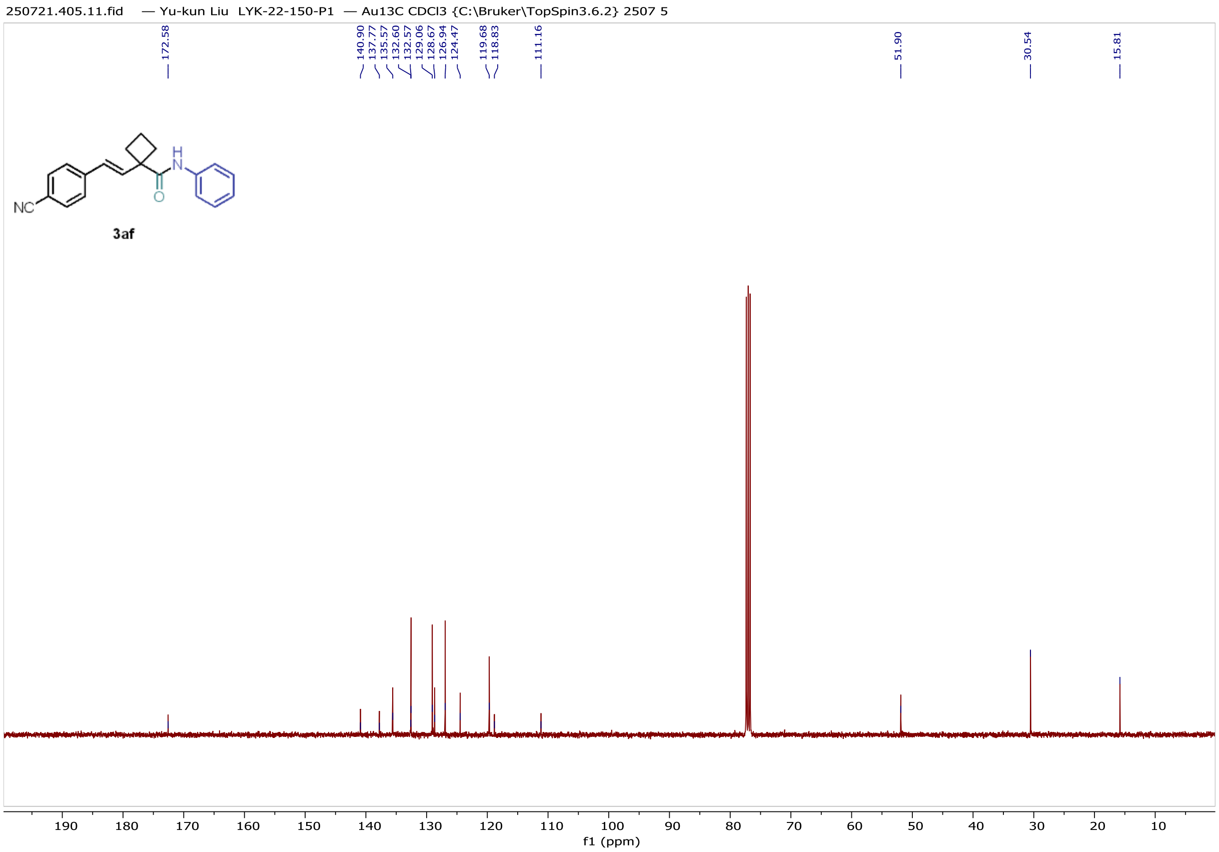

^{1}H NMR spectrum of **3ag** (300 MHz, $CDCl_3$)

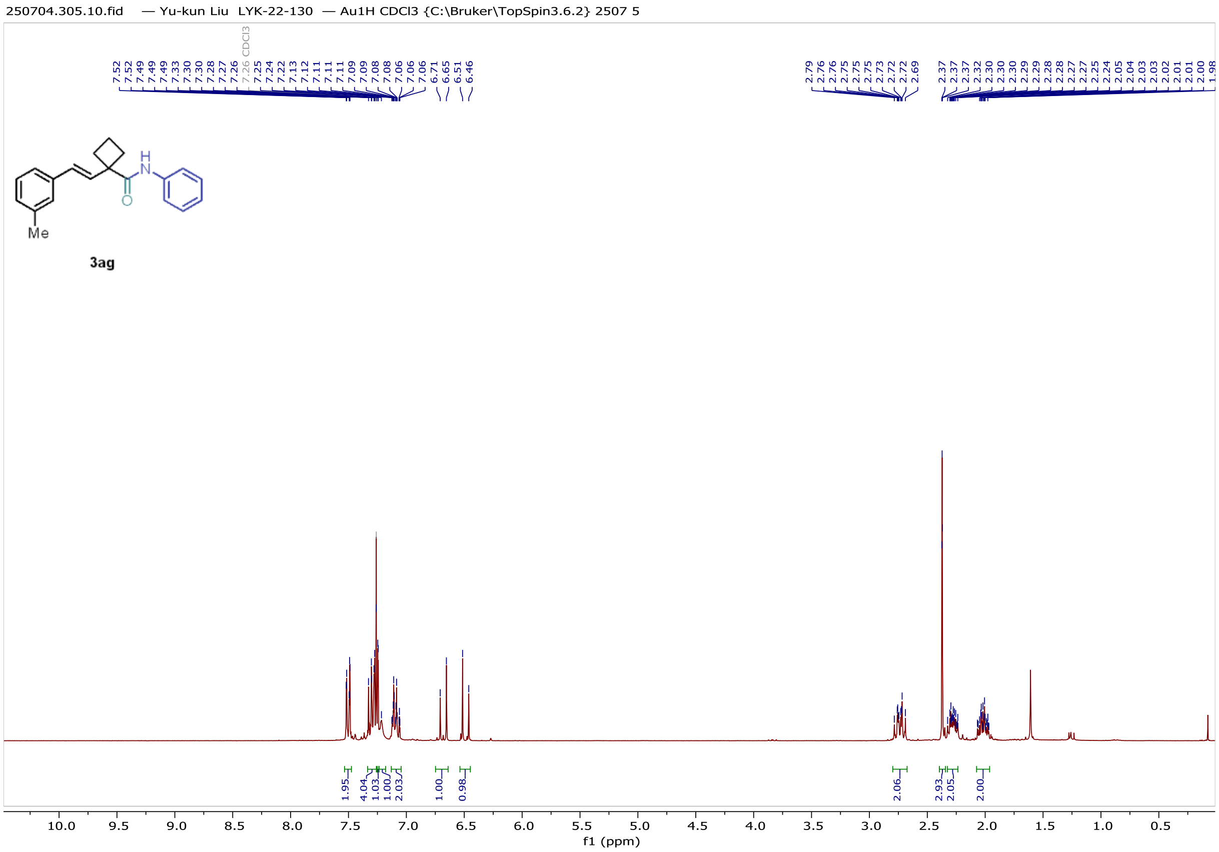

^{13}C NMR spectrum of **3ag** (75 MHz, $CDCl_3$)

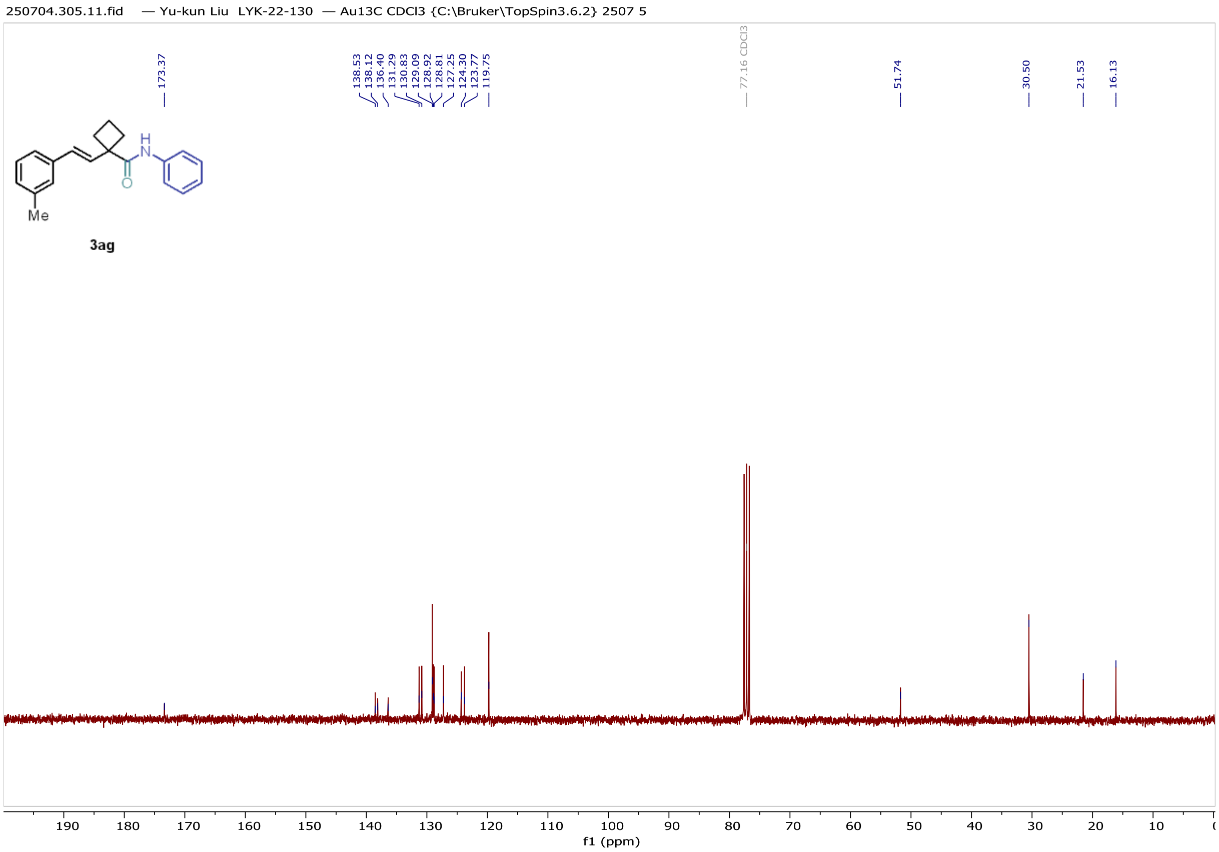

^{1}H NMR spectrum of **3ah** (400 MHz, $CDCl_3$)

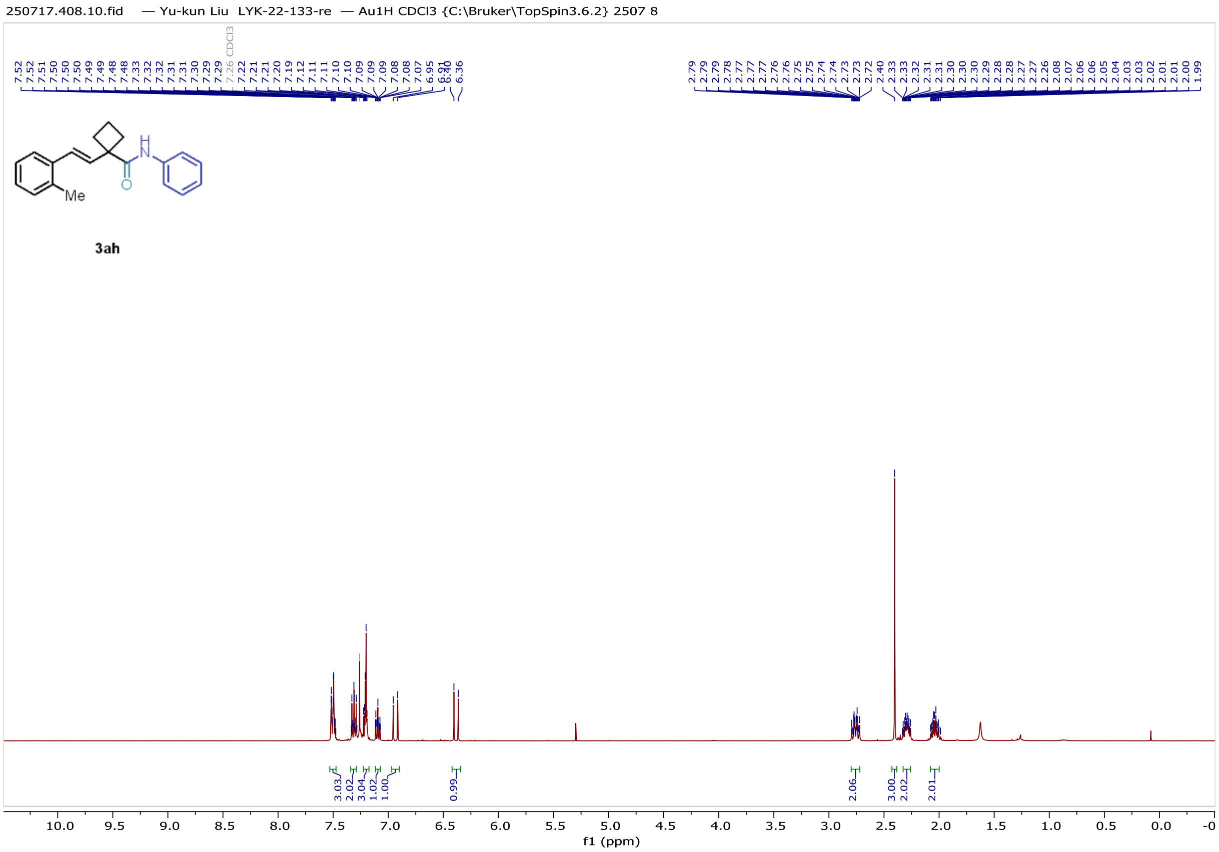

^{13}C NMR spectrum of **3ah** (101 MHz, $CDCl_3$)

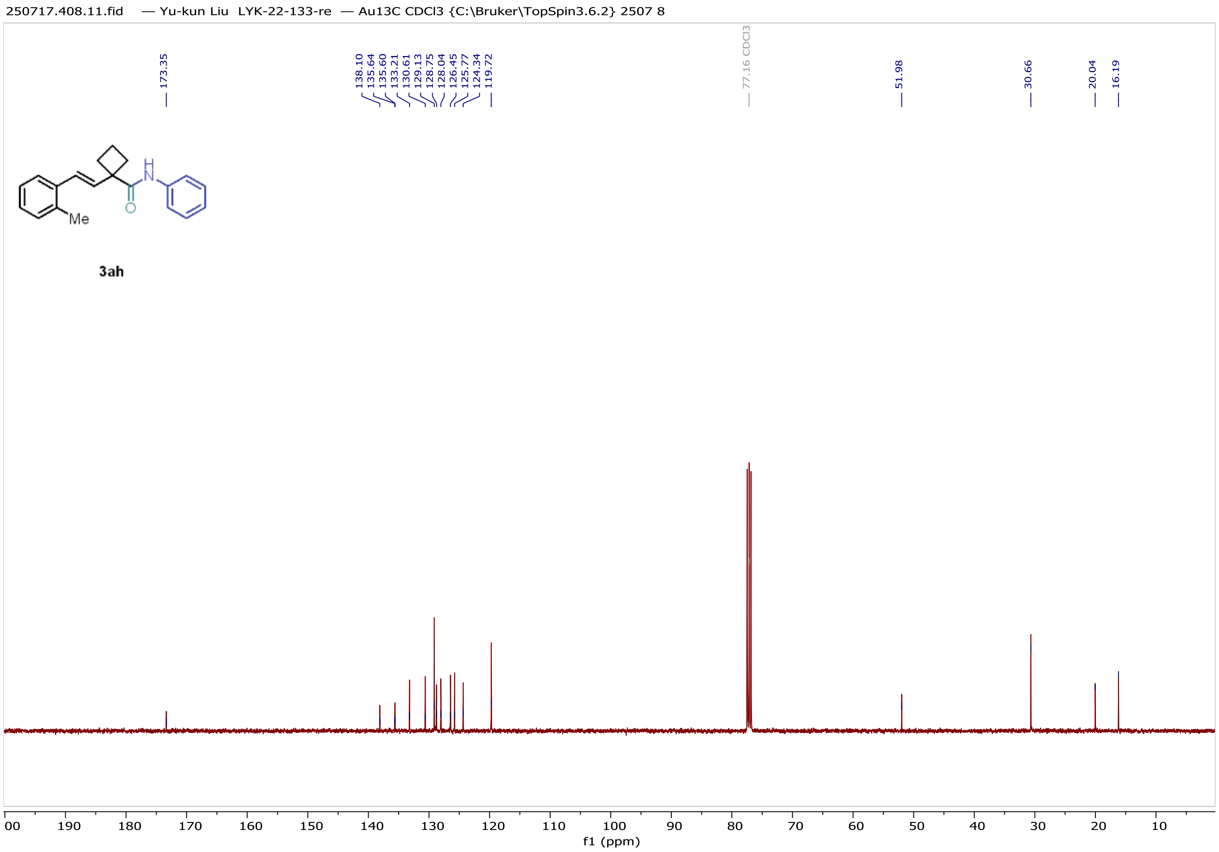

^{1}H NMR spectrum of **3ai** (400 MHz, $CDCl_3$)

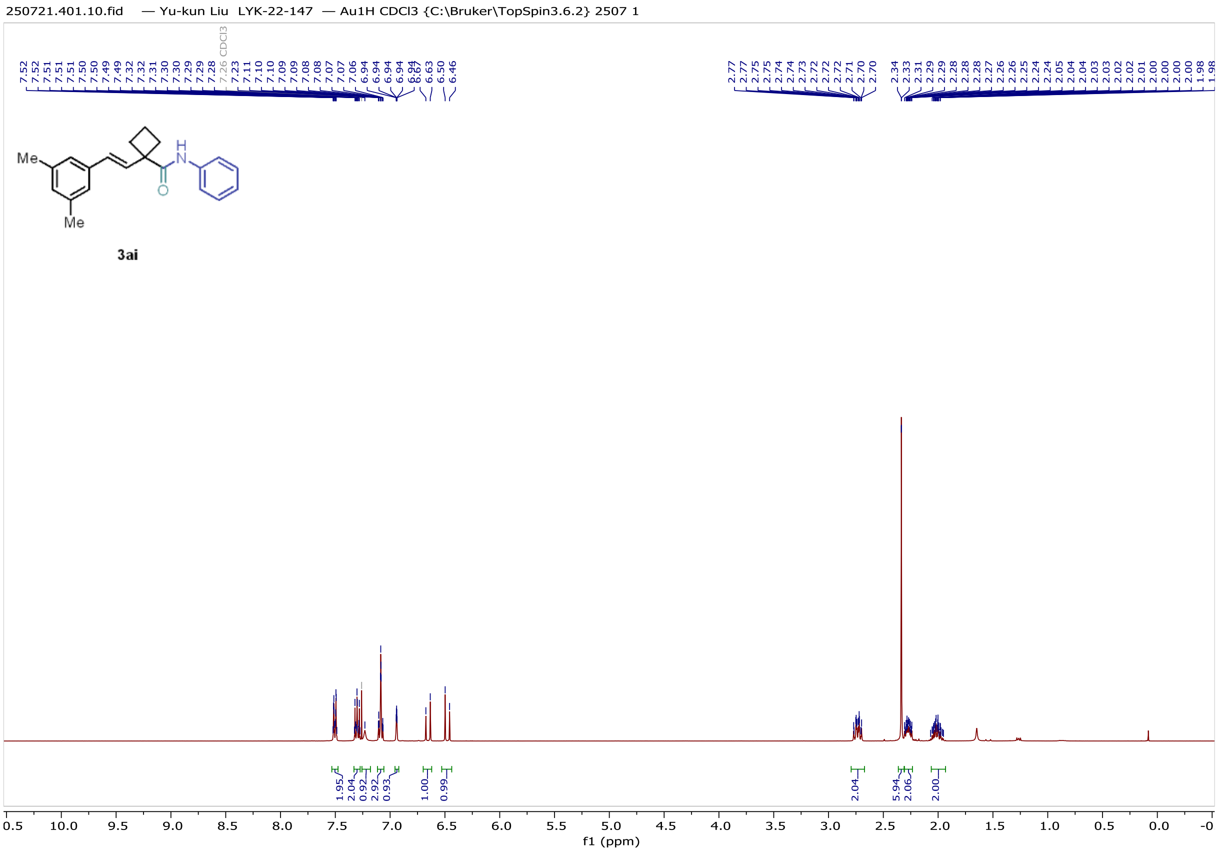

^{1}H NMR spectrum of **3ai** (101 MHz, $CDCl_3$)

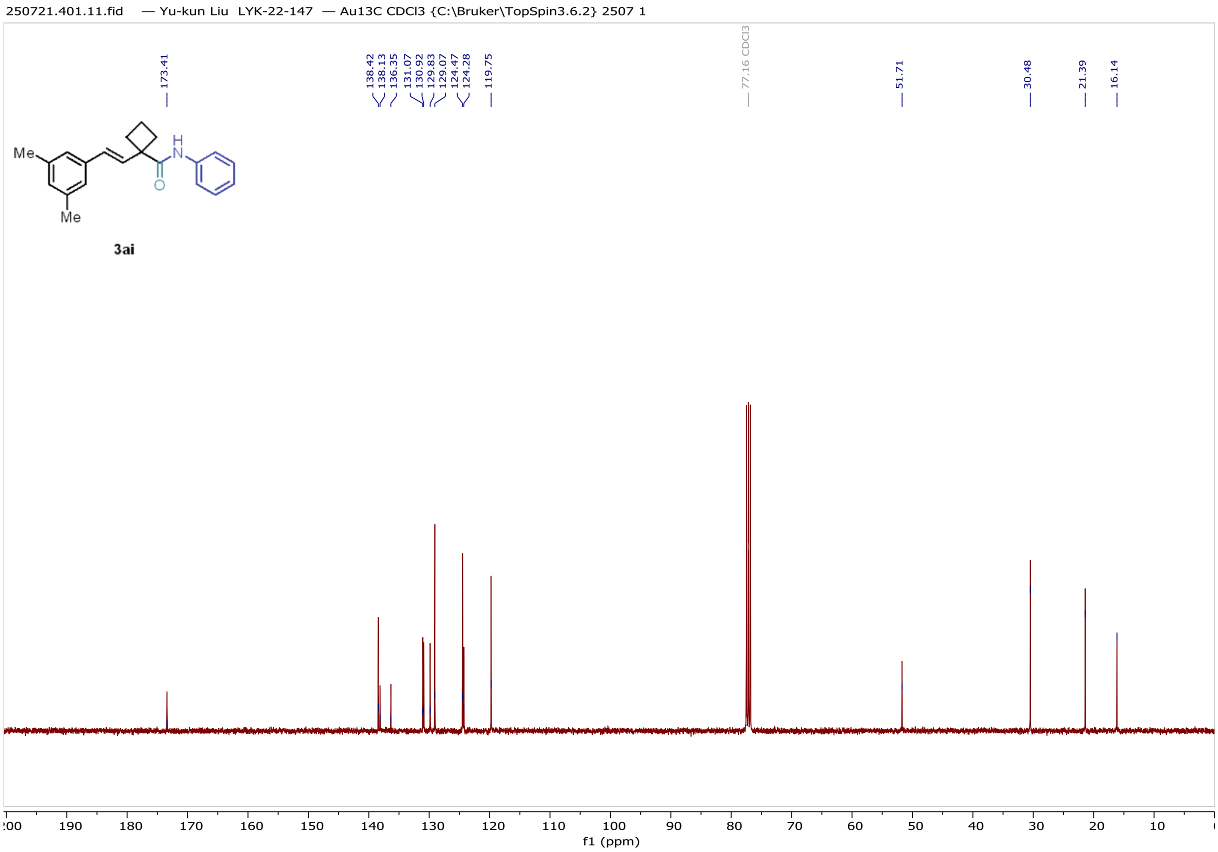

^{1}H NMR spectrum of **3aj** (400 MHz, $CDCl_3$)

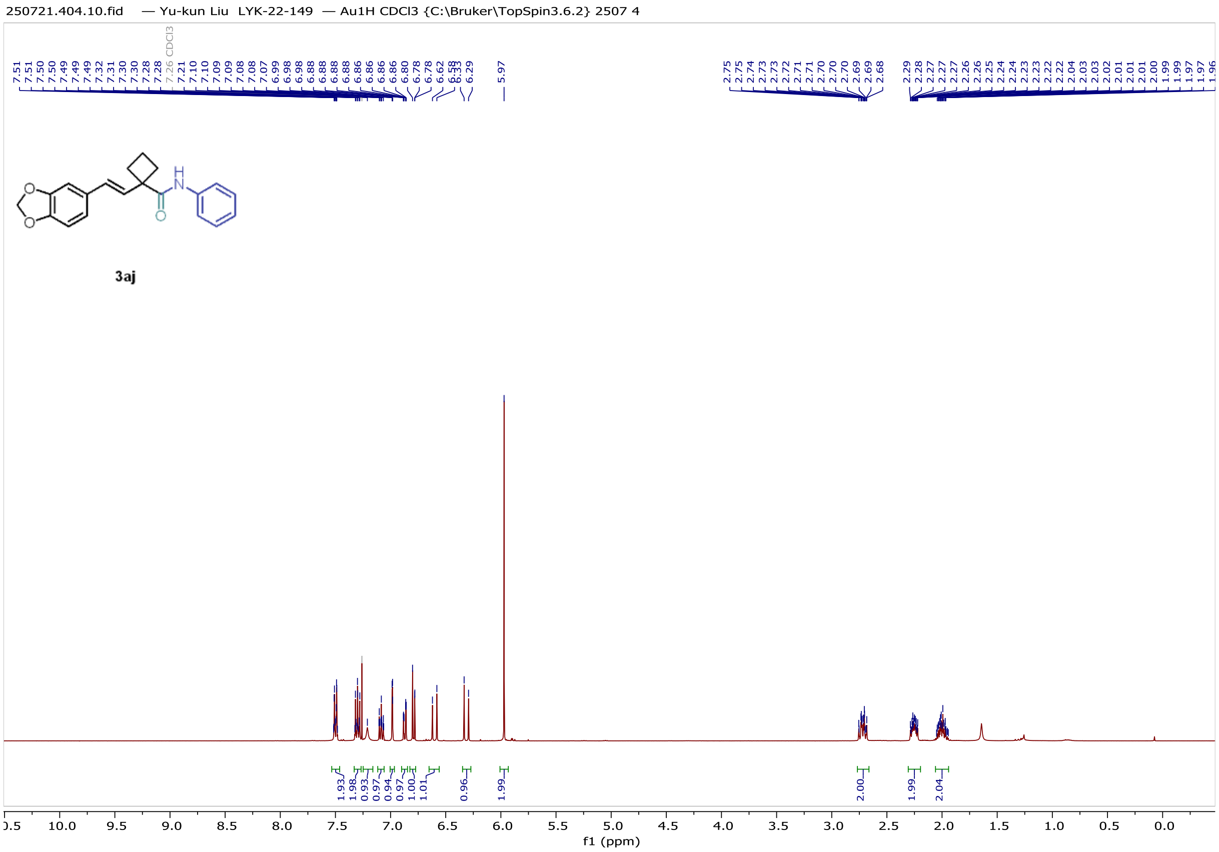

^{13}C NMR spectrum of **3aj** (101 MHz, $CDCl_3$)

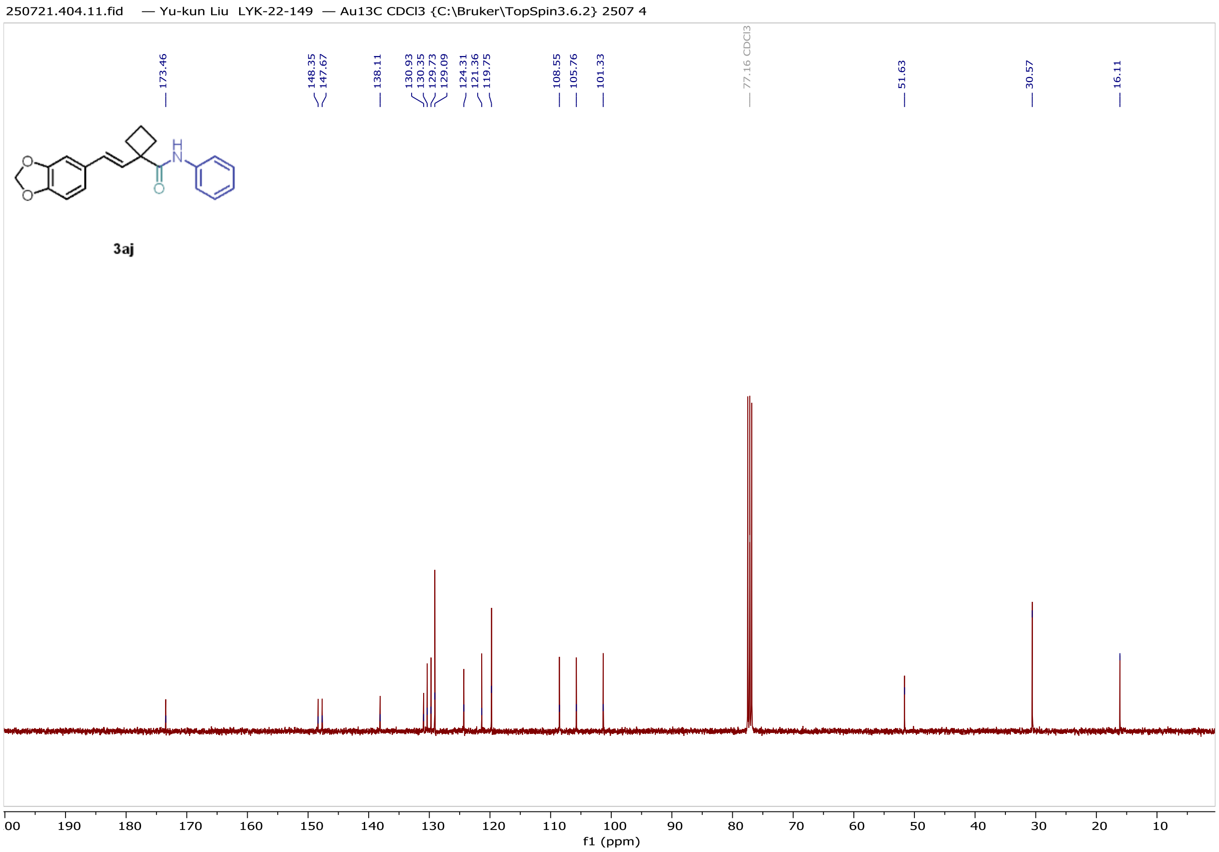

^{1}H NMR spectrum of **4** (300 MHz, $CDCl_3$)

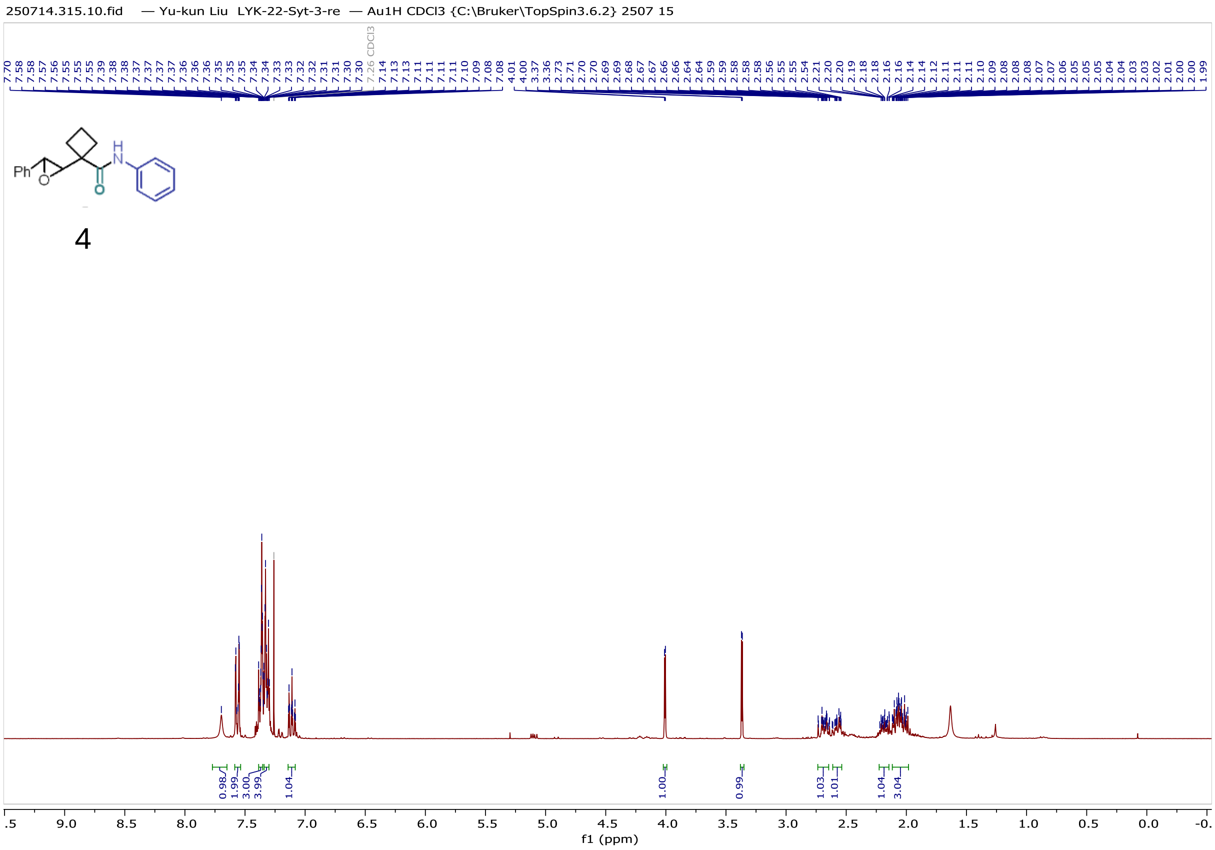

^{13}C NMR spectrum of 4 (300 MHz, $CDCl_3$)

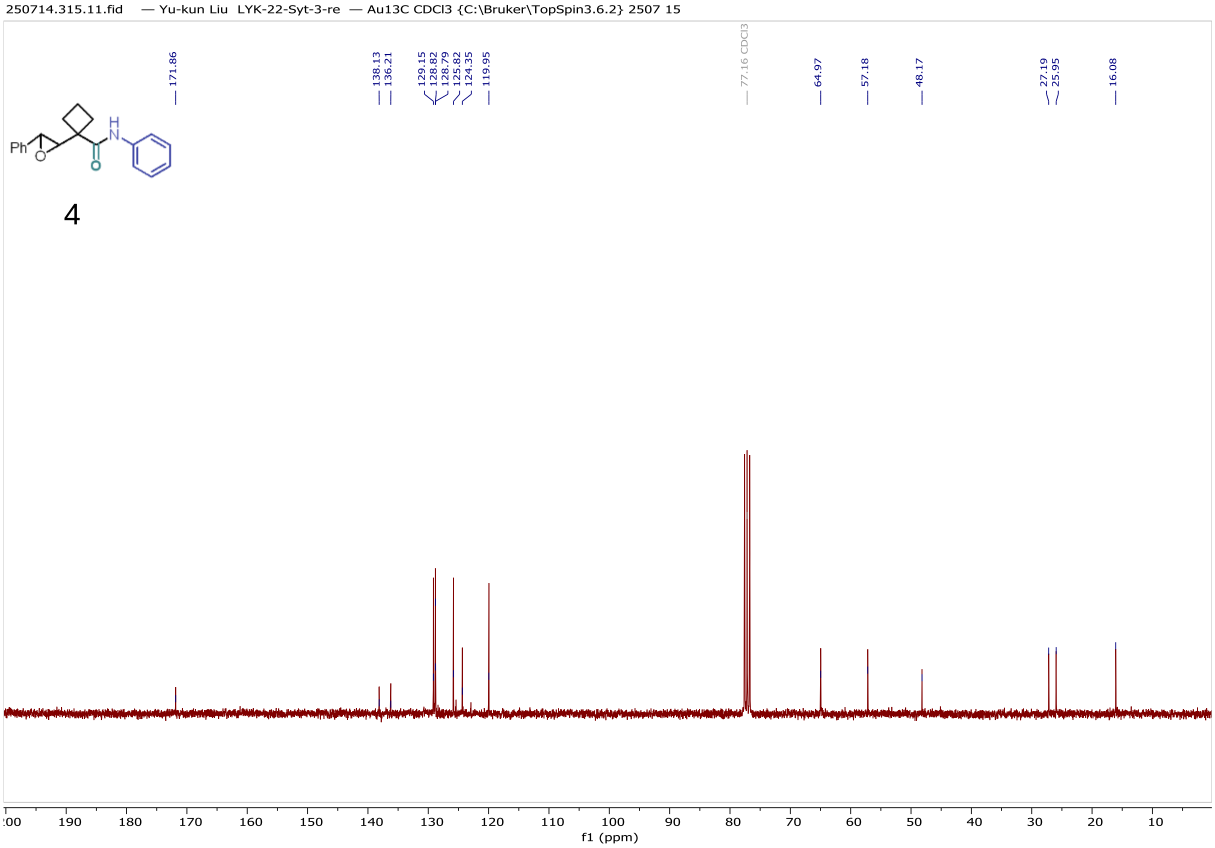

Supplement: Supplementary file 1 [file ol5c03781_si_001.pdf]
